# Physiological artefact control in real-time fMRI neurofeedback – A systematic review and meta-analysis

**DOI:** 10.1162/IMAG.a.1215

**Published:** 2026-04-13

**Authors:** Jingying Zhang, Franziska Weiss, Peter Kirsch, Martin Fungisai Gerchen

**Affiliations:** Department of Clinical Psychology, Central Institute of Mental Health, University of Heidelberg/Medical Faculty Mannheim, Mannheim, Germany; German Center for Mental Health (DZPG), Partner Site Mannheim-Heidelberg-Ulm, Berlin, Germany; Department of Psychology, University of Heidelberg, Heidelberg, Germany; Bernstein Center for Computational Neuroscience Heidelberg/Mannheim, Mannheim, Germany

**Keywords:** fMRI, neurofeedback, physiological artefact, systematic review, meta-analysis

## Abstract

Physiological artefacts are a general challenge in functional magnetic resonance imaging (fMRI) and are of particular relevance in real-time fMRI neurofeedback (rt-fMRI NF), where they can contaminate the training procedure and lead to undesired effects. However, physiological noise correction has not received corresponding attention in rt-fMRI NF and most attempts have mainly focused on offline data correction. Given the relatively rare use of physiological denoising methods in rt-fMRI NF and little knowledge about how these methods relate to reported outcomes, in this paper we review recent practices in physiology correction, and conduct a meta-analysis to summarize how reported outcomes vary across different correction approaches. A total of 227 studies were included. We found that physiology correction methods are still not very common offline (29.1%) and online (31.3%) and that even basic physiology recordings are still quite rarely reported in rt-fMRI studies (18.1%). This suggests that the implementation of physiological noise correction and use of concurrently recorded physiological signals is still in early stages. When summarizing reported outcomes of real-time target signal regulation, we found that studies employing online physiological correction did not systematically report smaller meta-analytic effect sizes than studies without such correction. Among correction methods, *‘imaging signal-based’* approaches were being the most commonly applied online. From a combined empirical and theoretical perspective, we recommend the use of online physiology corrections in rt-fMRI NF studies.

## 1 Introduction

Functional magnetic resonance imaging (fMRI), an indirect measure of neural activity based on blood oxygenation (Logothetis et al., 2001; Ogawa et al., 1990), is traditionally used to observe and understand brain activity and its relationship with behavior and/or cognition. Since the development of real-time fMRI (rt-fMRI) approaches (Cox et al., 1995), it has been shown that fMRI can not only be used for mapping brain activity but also as a tool to induce changes in brain activity by neurofeedback (NF), ultimately aiming at modifying behavioral and cognitive processes by the regulation of brain activity. Rt-fMRI NF is a non-invasive procedure in which the BOLD signal is measured and presented to the participant as a feedback signal in almost real-time. Through volitional control, individuals can learn to regulate their brain activity. Over the years, rt-fMRI NF has shown effects in both enhancing behavioral and cognitive performance in healthy individuals (for example (Dhanis et al., 2024; Paret et al., 2014)) and altering dysfunctional brain activity in patients with mental disorders with medium to large effect sizes (Dudek & Dodell-Feder, 2021; Pindi et al., 2022). Although still far from conventional use, rt-fMRI NF has promising potential to improve the way mental disorders are treated in the future. For realizing this potential, special focus has to be placed on methodological aspects in rt-fMRI NF, such as data correction and denoising.

The BOLD signal is inherently susceptible to various noise sources, including hardware drift, motion, and physiological fluctuations such as respiration and cardiac activity (Birn et al., 2008; Chang & Glover, 2009; Glover et al., 2000; Weiss, Zamoscik, et al., 2020). Generally, cardiac-related fluctuations arise from heart pulsations and spontaneous heart rate variability, and may cause shifts in the exact location of brain structures and cerebrospinal fluid (CSF) compartments (Dagli et al., 1999). Respiratory fluctuations, resulting from breathing-induced head motion, lung volume changes, and chest expansion, can alter the static magnetic field leading to fMRI images distortions (Hu et al., 1995). Equally important, variations in breathing affect arterial CO_2_ level and cerebral blood flow (CBF) and contribute to BOLD signal fluctuations (Birn et al., 2006, 2008). The risk of physiological artefacts strongly depends on the type of data and assessed brain regions. The cardiac fluctuations especially affect regions with large vessels (e.g., Circle of Willis) and CSF (Birn et al., 2006), but the link between respiration and fMRI time series has been described as complex (Bulte & Wartolowska, 2017). Critically, the risk of respirational noise artefacts is even more pronounced in functional-connectivity (FC) studies than in activation-based studies. Birn et al. (2006) have shown that resting-state FC analysis is strongly driven by variations in breathing depth and rate. Distinguishing these signals from the desired signal is particularly difficult (Glover et al., 2000); however, to reliably estimate neural activity, extensive denoising is crucial (Birn et al., 2006; Glover et al., 2000; Misaki & Bodurka, 2021).

Denoising the BOLD signal is even more important in rt-fMRI NF, where it serves as both the training signal (online) and the outcome measure (offline), and the success of training should depend on its validity in real-time (online). Accordingly, physiological denoising in rt-fMRI NF serves two distinct methodological roles, depending on whether it is implemented online or offline. Offline analysis allows for a comprehensive evaluation of rt-fMRI NF efficacy, helps to uncover underlying mechanisms, validates denoising and signal extraction strategies, and informs improvements for subsequent online protocols. Online analysis is essential for generating valid feedback, ensuring that participants train on meaningful signals, and enabling immediate monitoring of data quality and dynamic adjustment of the NF training process (e.g., in closed-loop NF training). Failure to correct physiological artefacts in online data may contaminate the NF procedures and lead to unwanted effects, for example the use of readily available physiological strategies such as changing breathing patterns to regulate the feedback signal (Thibault et al., 2018; Weiskopf et al., 2004; Weiss, Zamoscik, et al., 2020). Thereby, online correction may determine whether regulation reflects neural processes or physiological fluctuations, whereas offline correction may affect the validity of outcome estimation. Efforts have been made to evaluate applicable correction methods, and readily available strategies such as global signal regression (GSR) have been validated for online physiological correction (Misaki & Bodurka, 2021; Weiss et al., 2022). However, so far most attempts of physiology correction in rt-fMRI NF have mainly focused on offline data correction (Misaki et al., 2015; Sulzer, Sitaram, et al., 2013; Weiskopf et al., 2004). Several years ago, Thibault et al. (2018) conducted a critical systematic review to assess the efficacy of rt-fMRI NF and reported on the use of physiology correction. Among the assessed studies, they noted that only 37 out of 99 studies addressed respiratory confounds. Building on this, Heunis et al. (2020) used the same selection criteria and identified another 29 studies, but observed no improvement in the reporting of respiratory correction methods. The 2020 Consensus Report on neurofeedback studies (CRED-nf) checklist (Ros et al., 2020) also underscored the importance of reporting methods used for online data processing and artefact correction, specifically recommending the correction of cardio-respiratory artefacts in fMRI studies.

In practice, motion has long been recognized and routinely addressed in fMRI denoising, physiological artifacts, however, have not received equivalent attention. The dedicated NF toolboxes, such as Turbo-BrainVoyager (Sulzer, Haller, et al., 2013) and OpenNFT (Koush et al., 2017), both include online motion correction as a default component of real-time pipelines but do not include native modules for physiological noise correction. Neuroimaging platforms such as SPM (Penny et al., 2011), AFNI (Cox, 1996), and FSL (Smith et al., 2004) provide flexible frameworks and can incorporate extensions that enable physiological noise modeling (e.g., PhysIO, RETROICOR, PNM, or the AFNI utility *physio_calc.py* integrated into the *afni_proc.py* pipeline). Preprocessing frameworks such as fMRIPrep (Esteban et al., 2019) also generate physiological noise regressors (e.g., CompCor), but they are designed exclusively for offline analyses. For physiological noise correction, several established methods are commonly used across these toolboxes. RETROICOR (Glover et al., 2000) models cardiac- and respiratory phase–related fluctuations using concurrent physiological recordings, then incorporates them as nuisance regressors in a general linear model (GLM) to remove the variance from the BOLD signal. The PhysIO toolbox (Kasper et al., 2017) extends this by modeling respiration volume per time (RVT) and heart rate variability (HRV) to capture additional slow fluctuations. In contrast, CompCor (Behzadi et al., 2007) does not require external physiological signals but instead extracts principal components from white matter (WM) and CSF as nuisance regressors, and is commonly implemented in toolboxes such as CONN (Whitfield-Gabrieli & Nieto-Castanon, 2012) and fMRIPrep. The Physiological Noise Modeling (PNM) module (Smith et al., 2004) provides RETROICOR- and RVT/HRV-based modeling within the FSL framework.

In light of the growing literature on physiological artefacts in fMRI NF in recent years, we expected that this would also be reflected in the increasing number of physiology correction, and also physiology recording (a necessary step for denoising, estimating physiological noise, and evaluating correction effectiveness), over time. In principle, when correction is applied online, participants should be regulating a purer neural signal rather than a signal contaminated with physiological noise; when correction is applied offline, outcome measures derived from post hoc analyses may differ from those obtained from uncorrected training signals but should better reflect the intended neural changes. On the other hand, one possible reason for an infrequent adoption of physiological correction methods in rt-fMRI NF might be concerns that applying correction may overly disturb the neurofeedback signal and thus affect training outcomes.

To evaluate the current status of the application of physiological denoising methods in the field of rt-fMRI NF, and, for the first time, investigate their impact on the regulation of the target signal, this systematic review and meta-analysis investigates the use of correcting physiological noise (respiratory and cardiac) in both offline and online procedures. We describe the frequency of physiology correction and recordings, and report their changes over time. Furthermore, we investigate the effect of physiological noise online correction on target signal regulation and compare the different online correction methods. In line with the conceptual distinction between online and offline physiological correction, the meta-analysis presented in this study focuses on online implementations, as they directly affects the validity of the neurofeedback training signal. Based on these findings, we aim at giving recommendations for future investigations on physiology artefact control.

## 2 Methods

### 2.1 Study selection and inclusion criteria

We searched the terms (neurofeedback) AND ((fMRI) OR (rt-fMRI) OR (rt-fMRI) OR (functional magnetic resonance imag*) OR (functional MRI) OR (real-time fMRI) OR (real time fMRI)) across all Databases and all years in Web of Science and Pubmed on 24.11.2023. Our search criteria excluded preprints, reviews, systematic reviews and meta-analyses and the search was restricted to articles published in English. After automatic duplicate removal in EndNote, N = 101 articles were removed manually. This procedure resulted in N = 729 abstracts, which were individually screened by both first authors (F.W. and J.Z.). Reasons for exclusion at this stage included not being directly related to rt-fMRI NF (N = 300), not being an original study (N = 87), duplicates (N = 10), not involving human subjects (N = 2), and article type (N = 77; e.g., methods paper, data paper, case report, preprint, conference paper, brief report, study protocol, etc.). Based on this screening process, N = 253 articles were identified for retrieval. An additional N = 9 reports were additionally included at this stage to assess eligibility based on full text, as they were included in (Thibault et al., 2018) and met the inclusion criteria, resulting in N = 262. From these, we excluded articles that included secondary analyses (N = 25), were not directly related to rt-fMRI NF (N = 6), or where the article type did not match our criteria (N = 4). After removal of the latter, N = 227 articles were included in the review. A graphical representation of the review protocol is provided in Supplementary Figure S1 in Supplement 1.

### 2.2 Data extraction

From the included articles (N = 227), the following information was extracted: 1) Publication characteristics (title, authors, and year). 2) Was physiology correction applied? Was it applied online or offline? Which signal was taken into account (cardiac and/or respiratory?) 3) Which correction method was used? 4) Was physiological data recorded concurrently? 5) Participant characteristics (age, gender and diagnosis). 6) Target signal regulation (target region, results type, and results at the last run).

The different correction methods were categorized to: ‘*physiological signal-based*’, ‘*imaging signal-based*’, *‘(post-hoc) physiological signal comparison*’, ‘*instruction*’, and ‘*do not report* (*DNR*)’. This category scheme is similar but not equal to those in (Thibault et al., 2018). Studies using more than one method were counted for each applicable category.

1. ‘*Physiological signal-based methods*’ marks studies that used concurrently recorded physiological signals as regressors or model components to correct the BOLD signal and remove physiological artefacts.
2. *‘Imaging signal-based methods’* refers to studies that did not rely on physiological recordings but instead used imaging data. For instance, the signal in a region of interest (ROI) was adjusted (e.g., by subtraction) using the signal from another ROI, a larger brain region such as WM or CSF, or the whole brain. We further subdivided these methods into ‘*regression/subtraction’*, ‘*GSR’*, ‘*independent/principal component analysis (ICA/PCA)’*, and ‘*non-voxel-wise whole-brain* methods’ (i.e., subtraction or adjustment based on whole-brain or network-level signals rather than voxel-wise modeling), depending on the instruments and computational approaches used.
3. ‘*(post-hoc) physiological signal comparison*’ includes studies that examined respiration rate and/or heart rate across groups or conditions to rule out confounding during offline, but this method did not directly correct the fMRI data. It should be noted that some studies (e.g., (Marxen et al., 2016; Pamplona et al., 2020) recorded respiration and heart rate concurrently and processed them, but used these signals only for post-hoc comparisons or correlation analyses, and not as regressors or model components for correcting the BOLD signal. Thus, they were classified as ‘*physiological signal comparison*’ instead of ‘*physiological signal-based correction*’.
4. ‘*Instruction*’ means that participants were instructed at the start of the NF training to keep their breathing steady and to refrain from using breathing as a strategy to alter brain activity. While this does not represent a correction method per se, we listed it as a separate category for online, to reflect researchers’ awareness of both the potential influence of respiration on the BOLD signal and the possibility that participants may use breathing as a strategy to modulate brain activity.
5. We chose ‘*DNR*’ as a label rather than ‘no correction applied’ to illustrate that in some cases a correction might have been applied, even though it was not mentioned in the text.

We extracted group means and standard deviations (SDs) of the target signal regulation when available. When means and SDs were not reported, we obtained t or F values from the text and tables. If results were only presented graphically, we extracted them from figures using WebPlotDigitizer (Rohatg, 2024). These values were then used to compute effect sizes of the group comparison. The experimental group is defined as using real NF, and the control group is characterized by providing sham or yoke NF. Paper were excluded from the meta-analysis if they did not report sufficient data for group comparisons. All 227 studies included in this review are listed in Supplement 1 with bibliographic information. Detailed information on study characteristics, physiological recordings and correction methods, as well as extracted quantitative data, are provided in Supplement 2.

### 2.3 Meta-analysis of the effect of NF on target signal regulation

The meta-analysis was performed in SPSS (Version 29.0. Armonk, NY: IBM Corp). Effect sizes of the group comparison were reported with Cohen’s D (Cohen, 2013). Positive effect sizes in our analyses indicate a change in the predicted direction of the NF training effects. The between-group effect size was based on the training effects at the last NF run. A random-effects model (Cuijpers et al., 2016) was used to pool effect sizes. The heterogeneity was quantified by Q, I^2^ and τ^2^ (Borenstein et al., 2017; Higgins & Thompson, 2002). Q value assessed the homogeneity and its significance represented the pooled true effect vary. I^2^ indicates the extent of heterogeneity, ranging from 0% (no heterogeneity) to 100% (all variation in observed effects can be fully explained by differences in true effects). τ^2^ represents the variance of the true effect. To examine the differences between physiology-corrected and -uncorrected studies, as well as between various correction methods, we conducted subgroup analyses. Funnel plots (Sterne et al., 2001) and Egger’s regression test (Egger et al., 1997) were used to assess potential selective reporting and publication bias. The corresponding results were presented in Supplement 1, organized under the corresponding analysis headings.

## 3 Results

Here, we present the frequency of physiological data recording and physiological artefact correction, the distribution of correction methods, and meta-analytic summaries of reported outcomes under different online analysis conditions and across different online correction methods.

### 3.1 Distribution of physiological data recording and physiological artefact correction

Out of the 227 included studies, we found that only 18.1% (N = 41) of studies made any reference to physiology recording. Overall, 47.1% (N = 107) of all studies were found to have corrected for physiological artefacts in any way. When examining offline and online correction separately, offline correction (N = 66, 29.1%) is slightly less common than online correction (N = 71, 31.3%). Regarding the type of physiological signal, almost all studies corrected both respiration and cardiac artefacts (N = 99), whereas N = 8 corrected respiration alone. No study reported correcting cardiac artefacts without correcting respiration.

**Fig. 1.**
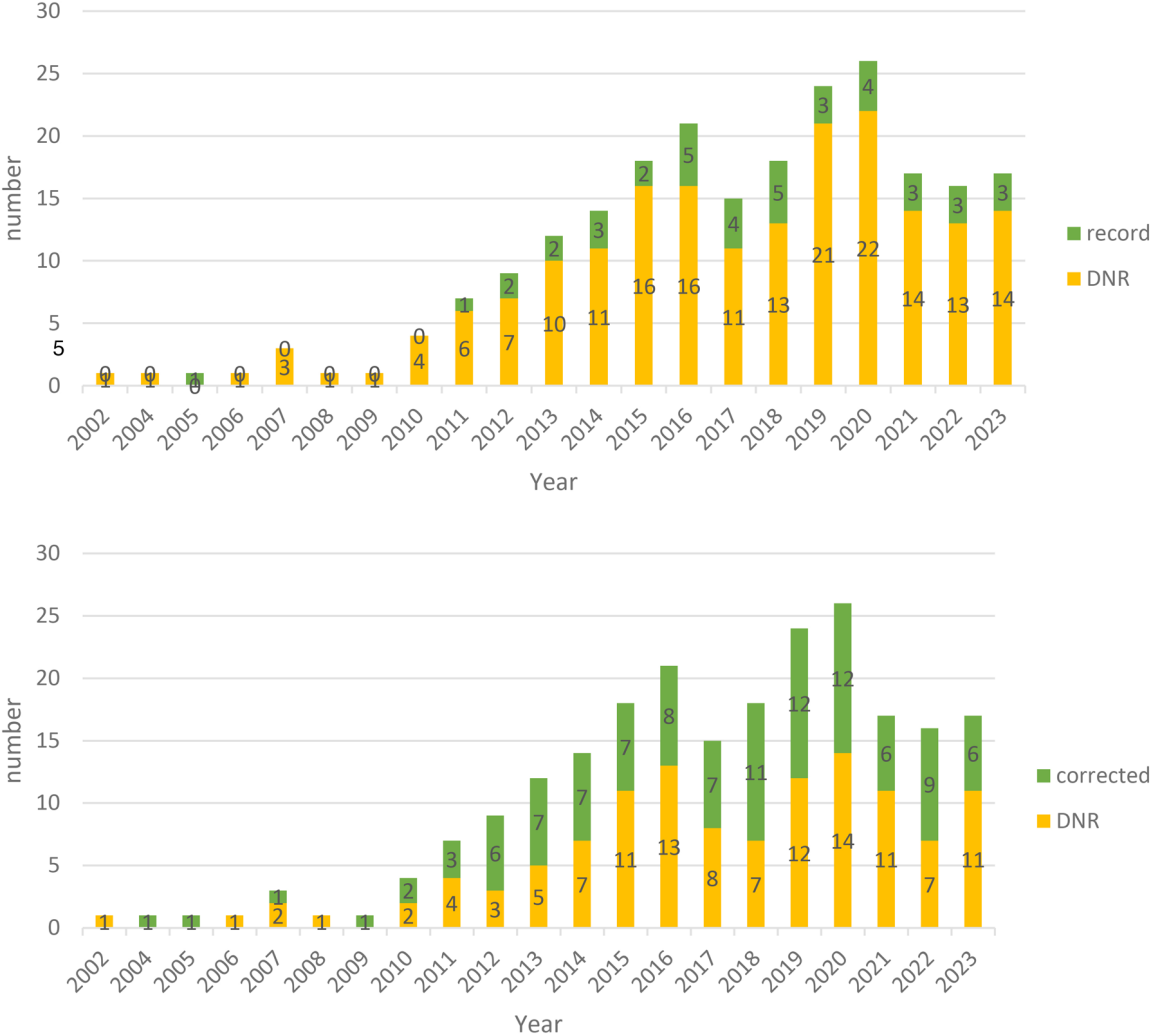
Distribution of recordings and corrections of physiology over years, up: recording, down: correction. The number represents the absolute number of studies that report correction or recording (in green). For correction, studies were counted as corrected if they conducted at least one correction, whether offline or online, for either respiratory or cardiac artefacts. Similarly, studies reported respiratory or/and cardiac recording were counted as record. Otherwise, studies were categorized as do not report (DNR, in yellow).

The proportion of studies recording physiological data has remained relatively stable over the years. With respect to the application of physiology correction, following a period of increased application in 2019 and 2020, its share remained stable (Fig. 1).

### 3.2 Distribution of correction methods

The correction methods for respiratory and cardiac artefacts were similar across studies; thus, we report the results for respiratory artefacts here, while those for cardiac can be found in Table 1 and Supplementary Figure S2.

**Table 1.** Distribution of correction methods.

	Respiratory		Cardiac	
	Offline	Online	Offline	Online
0-DNR	161	156	162	167
1-Physiological signal-based	26	6	25	6
2-Imaging signal-based	42	58	42	58
2.1 Regression/subtraction	32	51	32	51
2.2 GSR	10	7	10	7
2.3 ICA/PCA	10	1	10	1
2.4 Non-voxel-wise whole-brain	1	4	1	4
3-Physiological signal comparison	15	0	15	0
4-Instruction	0	15	0	1

The absolute number of studies using each method is shown separately for respiratory and cardiac artefacts, and for offline and online analyses. Studies that used more than one method were counted for each method.

Of the 227 studies we reviewed, 70.9% (N = 161) of the studies did not report correction for offline, 11.5% (N = 26) of the studies corrected by ‘*physiological signal-based’* methods, 18.5% (N = 42) corrected by ‘*imaging signal-based*’ approach, and 6.6% (N = 15) by means of *‘physiological signal comparison*’. For the subgroups of the ‘*imaging signal-based*’ approaches, 42 studies (18.5%) used regression/subtraction, 10 of which applied GSR, another 10 (4.4%) applied ‘*ICA/PCA’*, and one study (0.4%) used ‘*non-voxel-wise whole-brain* methods’.

Online correction was not reported in 68.7% (N = 156) of studies, with the second most common correction being ‘*imaging signal based*’ (25.6%, N = 58), followed by ‘*instruction*’ (6.6%, N = 15). The remainder used ‘*physiological signal-based*’ methods (2.6%, N = 6). Within the ‘*imaging signal-based*’ methods, ‘*regression/subtraction’* was the most frequently reported (22.5%, N = 51), followed by ‘*GSR’* (3.1%, N = 7) and ‘*non-voxel-wise whole-brain methods’* (1.8%, N = 4), with one study (0.4%) using *‘ICA/PCA’* (Kim et al., 2019).

### 3.3 Effect of online physiological artefacts correction

To assess the impact of online physiological denoising, we meta-analytically compared the target signal regulation effect between the experimental and control groups at the last NFB run. We pooled all target regions together and report the results here; analyses separated by region types (cortical and subcortical) are provided in Supplement 1 under the corresponding analysis sections.

**Fig. 2.**
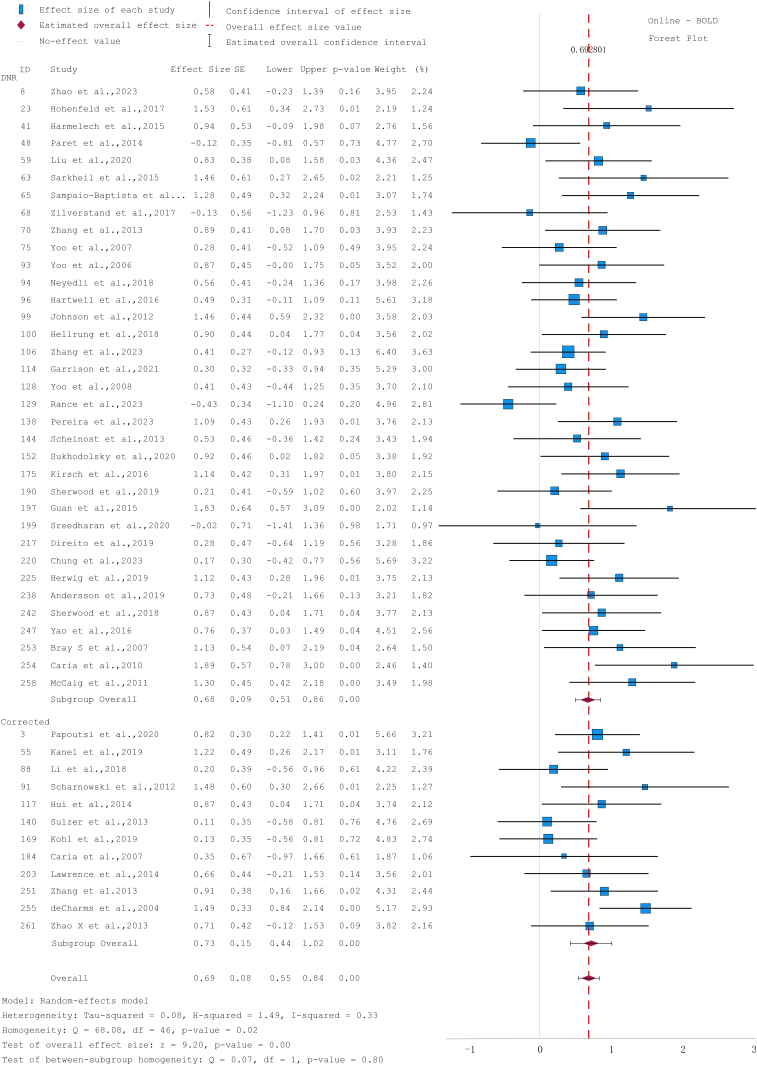

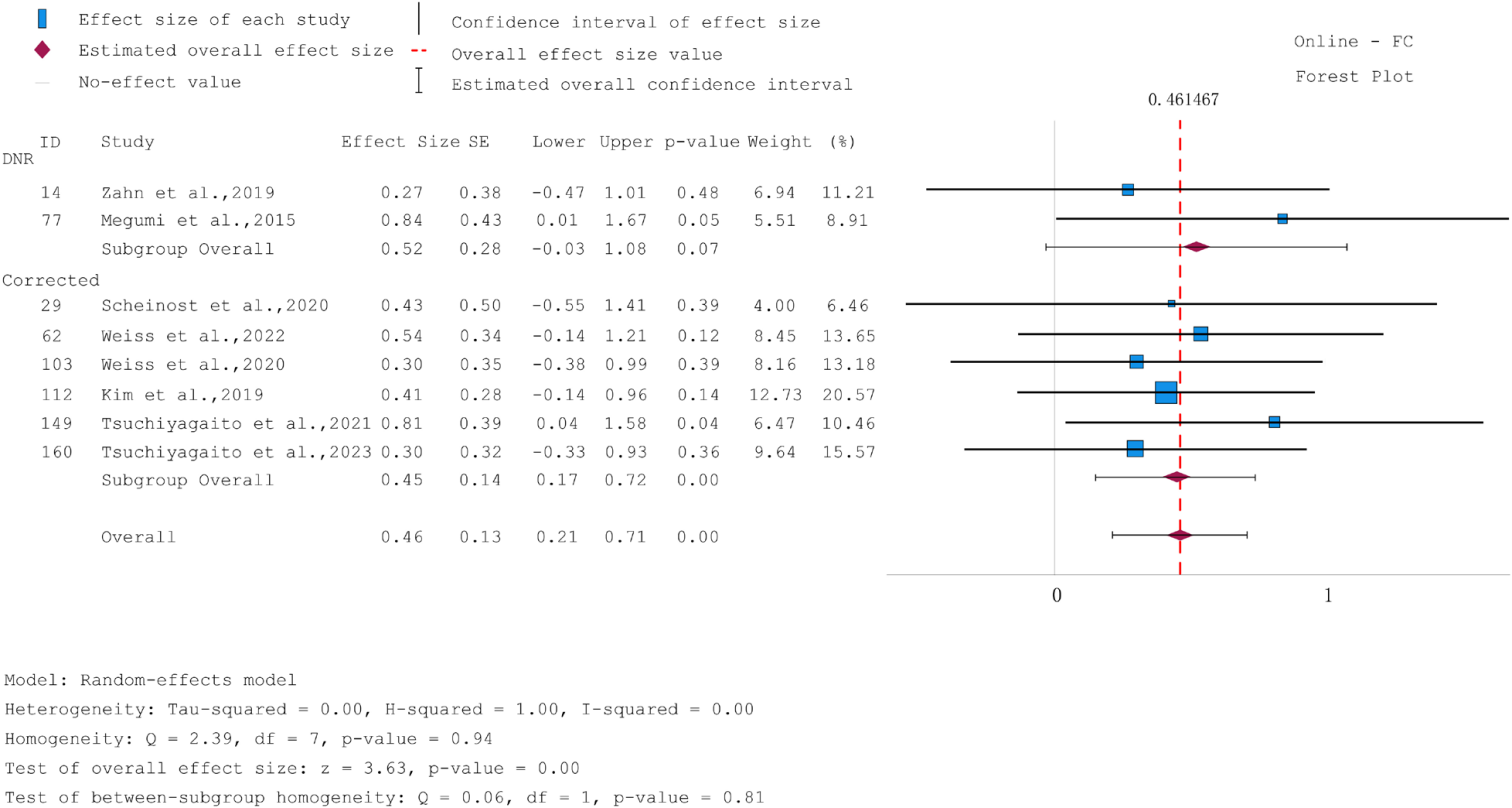
Forest plot of between-group effect sizes examining the impact of online physiological correction on (a) BOLD activation measures and (b) FC measures. Studies were counted as corrected if they conducted online correction for either respiratory or cardiac artefacts. Otherwise, studies were categorized as DNR. The solid grey line indicates zero effect, and the red line represents the overall effect size. Positive values mean that the brain signal was regulated in the expected direction. CI = confidence interval, d = Cohen’s effect size, SE = standard error.

For the BOLD activation measures (N = 47), the overall analysis revealed a significant effect size (d = 0.69, 95% CI [0.55, 0.84], p < 0.001). Subgroup analyses also showed significant medium-to-large effects for both studies without physiological noise correction (d = 0.68, 95% CI [0.51, 0.86], p < 0.001) and studies with correction (d = 0.73, 95% CI [0.44, 1.02], p < 0.001). The between-subgroup homogeneity was not significant (Q=0.07, p=0.80). Heterogeneity across studies was low to moderate (I² = 0.33, τ² = 0.08, Q = 68.08, p = 0.02) (Fig. 2a). Funnel plots and Egger’s regression tests revealed no evidence of publication bias (all ps > 0.05).

With regard to FC measures (N=8), the between-group analysis showed a significant medium effect overall (d = 0.46, 95% CI [0.21, 0.71]) and for studies with correction (0.45, 95% CI [0.17, 0.72]) but not for FC studies without correction (d = 0.52, 95% CI [-0.03, 1.08]) (Fig. 2b). When pooling all measure types together (N=55), the between-group analysis indicated significant medium-to-large effect for both correction (d = 0.62, 95% CI [0.42, 0.82]) and DNR studies (d = 0.67, 95% CI [0.51, 0.83]), as well as for overall (d = 0.65, 95% CI [0.52, 0.77]) (Supplementary Fig. S3).

### 3.4 Effect of different online correction methods

**Fig. 3.**
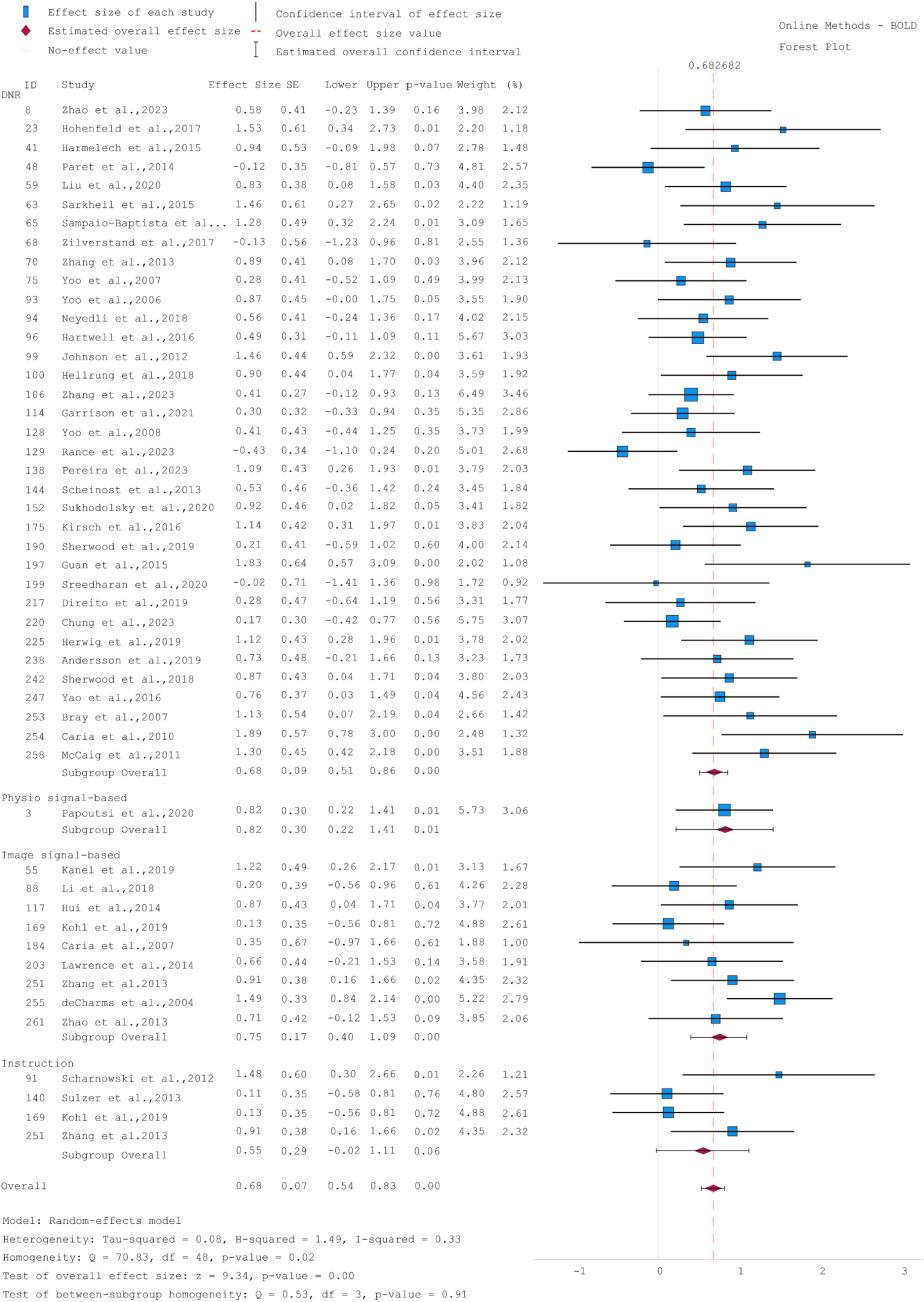
Forest plot of between-group effect sizes examining the impact of different correction methods on BOLD activation measures in online analyses. Studies were labeled as DNR if no correction was applied. The solid grey line indicates zero effect, and the red line represents the overall effect size. Positive values mean that the brain signal was regulated in the expected direction. CI = confidence interval, d = Cohen’s effect size, SE = standard error.

We grouped different online correction methods. Here, we present the results for BOLD activation measures. Studies without online physiological correction showed a medium significant effect (d = 0.68, 95% CI [0.51, 0.86]). Studies using ‘*image signal-based*’ correction methods revealed a slight higher effect (d = 0.75, 95% CI [0.40, 1.09]). Since only one study applied ‘*physio signal-based*’ method, and fewer than five studies used ‘*instruction*’, these results are not presented here. The heterogeneity analysis indicated moderate between-study variability (I^2^ = 0.33; τ^2^ =0.08; Q=70.83, p = 0.02) (Fig. 3). The funnel plot suggests a relatively balanced inclusion of studies, and Egger’s regression test indicated non-significant asymmetry (ps > 0.05).

## 4 Discussion

This systematic review and meta-analysis investigated the frequency of physiology recording and correction in rt-fMRI NF research, the use of the most common physiological correction methods, and the impact of physiology correction methods on target signal regulation.

### 4.1 The physiological recording and correction

Our results show that recording of physiological signals is still quite rarely reported in NF studies (18.1%). While recording physiology is not synonymous with correcting for physiology, physiological recordings should, thus, always be acquired as a standard routine. In practice, the costs of physiology recording are relatively low in terms of time. Simple measures such as pulse oximetry and respiratory belts provide sufficient information for most denoising purposes, and are far less invasive and time-consuming than full electrocardiography (ECG). Therefore, physiological data should be collected anyways to obtain information for denoising, estimate the influence of physiological noise in the data, and evaluate the effectiveness of denoising procedures. Almost certainly, the actual rate of physiological recordings was higher, but likely several studies did not report about it when they did not make use of the data. To note, although the recorded physiological signals can in principle be used for online correction, they are still relatively uncommon in practice and are more frequently applied in offline analyses for validation, control, or post-hoc modeling.

In general, nearly half of the studies report some way of physiology correction (47.1%) when both online and offline are counted, which is a relatively high proportion. However, it was found that physiology correction methods are still not very common in rt-fMRI NF research when offline (29.1%) and online correction (31.3%) are considered separately. Based on our assessment of changing over time, the rate of physiology recordings and corrections has remained static over recent years, subsequently stabilizing. Despite the potential for enhancement in the rate of correction applied, a significant proportion of papers included in this review recognized the absence of physiological correction as a limitation. Thus, the awareness of this issue may be higher than the numbers obtained in the study. There is clearly growing interest in this topic among the NF community (Heunis et al., 2020; Misaki & Bodurka, 2021; Weiss, Zamoscik, et al., 2020), the importance of physiology correction is constantly emphasized in the literature and at conferences, and the potential consequences of training on aspects other than brain activity are taken seriously.

In the present analyses, we separated online and offline correction. This is of particular significance given that, in contrast to a typical fMRI study, in rt-fMRI NF the online signal holds increased relevance as it is the value that is fed back to the participant trained in the scanner. As such, offline correction would not suffice because an inherent biased online signal might not be removable afterward. It is noteworthy that, when leaving out *‘instruction’* (counted as online only) and ‘post hoc comparison’ (counted as offline only)—which does not represent a genuine correction—online and offline correction interestingly appeared to be equally prevalent. For rt-fMRI NF approaches, the implementation of online correction is more complicated compared to offline analyses, as online processing is performed under real-time conditions with a limited number of samples available, particularly early in a scan. Although image reconstruction, denoising, target signal derivation, and feedback presentation needs to be carried out in less than the repetition time (TR), recent implementations have demonstrated that such computations can be performed within 0.5 seconds (Misaki et al., 2022). However, the limited sample size available during online analysis constrains the reliable estimation of low-frequency physiological fluctuations, which can reduce the effectiveness of approaches such as RVT-based correction (Misaki & Bodurka, 2021). Still our results revealed that online correction has become as prevalent as offline correction, indicating its growing feasibility and adoption in rt-fMRI NF studies.

We additionally considered cardiac and respiratory correction separately, and found that respiration correction appears to be slightly more common than cardiac correction. This is not unexpected, as this issue has also been discussed more frequently in the literature (Birn et al., 2006; Thibault et al., 2018; Weiss et al., 2022; Weiss, Zamoscik, et al., 2020). When comparing the influence of respiration versus cardiac activity on second-level BOLD activation, the influence of the former is more pronounced (Weiss, Zamoscik, et al., 2020). Interestingly, although these signals differ in physiological mechanism, we observed a similar distribution of denoising methods for both types. Notably, some physiological correction methods in our *‘imaging signal-based methods’* category do not distinguish between cardiac and respiratory correction targets. Therefore, the focus should be more on the method used and less on whether the correction aims at cardiac or respiratory correction.

### 4.2 The impact of physiological correction

We conducted a meta-analysis of the group comparison effect (real NF vs. sham/yoke NF group) for the last NF run, and a subgroup analysis to compare the impact of applying online physiological correction versus no correction. Across studies, reported effect sizes were comparable between those employing online physiological correction and those using uncorrected online signals. This is an important result, as a prevailing concern in the field is that overly stringent correction may make self-regulation more difficult or reduce training effects, for example, when GSR is applied on FC NF studies (Weiss, Aslan, et al., 2020). Here, we show that this concern is unfounded by providing evidence that studies employing online physiological correction do not systematically report smaller effects than uncorrected studies. In fact, the discrepancy in effect sizes between physiology correction and no correction in the last NF run was small, with both being statistically significant. Despite the statistically significant Q tests, the corresponding I² values indicated low to moderate heterogeneity across our results. Given the large sample size (>50 studies), the Q test may be overly sensitive to differences across studies; thus, the observed heterogeneity was considered acceptable.

The lack of difference in effect sizes between online physiological corrected and uncorrected studies may raise the question of how physiological correction influences reported outcomes. However, comparable effect sizes should not be interpreted as evidence that physiological correction has no effect or is unnecessary. First, there are examples like our previous study (Weiss, Aslan, et al., 2020) where it could be clearly demonstrated that significant group differences in the target signal, in this case fronto-striatal FC, disappeared after applying offline physiological artefact correction. Further, an interpretation that participants regulate the same neural signal regardless of correction seems unlikely to hold. Rather, despite targeting different underlying signals—in uncorrected studies participants may regulate physiological signals (e.g., respiration, heart rate), while in corrected studies they learn to regulate the actual neural signal—both regulation processes appear to yield comparable effect. Assuming that the effect of a study should be directly linked to the actual regulation of the target process, we would predict that a neutrally more valid online signal corrected for physiological artefacts should be associated with better clinical outcomes at the same level of target signal regulation. Unfortunately, the number of studies included in our meta-analysis that report sufficient data is rather limited, and this number would be further reduced if we were to include only studies report psychological or clinical measures. Therefore, this hypothesis needs to be tested in future studies when a larger database for such comparisons is available.

While physiological noise correction can be considered crucial, it also should be noted that physiological fluctuations are not necessarily mere noise or artefacts. Respiratory rhythms and heart rate variability are closely related to vigilance and arousal, which may constitute the training targets in certain NF tasks. Thus, while physiological correction can reduce non-neural noise and facilitate NF training, it may also remove signals that are relevant for some NF protocols. The decision to apply correction, and to use which method, should therefore depend on the specific NF target and the desired outcome.

### 4.3 Different correction methods

We have differentiated between five forms of physiology correction methods. By categorizing different correction methods and summarizing their effect sizes, we aimed at characterizing how different correction methods are represented in the existing rt-fMRI NF literature. The two methods ‘*instruction*’ and ‘*physiological signal comparison*’ were used too infrequently, which precluded any meaningful conclusions regarding their efficacy. While it has been proposed that participants should be told beforehand to be more aware of their breathing when performing a task, as sometimes respiration patterns are influenced by the task at hand (Bulte & Wartolowska, 2017; Farthing et al., 2007; Huijbers et al., 2014), and task-correlated breathing patterns may be difficult to regress out (Bulte & Wartolowska, 2017). NF studies should therefore instruct participants at the beginning of the training that respiratory changes should not be used as an NF strategy.

With regard to the remaining three categories, it can be concluded that in terms of online correction, *‘imaging signal-based methods’* were the most frequently applied and were found to have comparable effect sizes as ‘*DNR*’, and both were significant. In general, *‘imaging signal-based methods’* correction is a reliable choice that is not overly complex and straightforward to implement. While due to the limited numbers of available studies, we were not able to conduct separate meta-analyses for its sub-categories. Importantly, comparable effect sizes across correction approaches do not imply that different methods affect the same underlying signals. While it is possible to combine different correction methods, it must be noted that overfitting increases the risk of more substantial errors when compared with a basic analysis (Bulte & Wartolowska, 2017).

While our meta-analysis did not yield sufficiently distinctive patterns to allow for a clear ranking of correction methods, examining the methods themselves may still offer reference for future applications (see detail in Supplementary Note). In summary, ‘*physiological signal–based methods’* (such as RETROICOR, PhysIO) are feasible and preferable from a methodological standpoint but require synchronized physiological recordings and additional computational resources which makes thems more demanding to implement online. In contrast, online *‘imaging signal–based approaches’* are more straightforward to implement in real-time pipelines and have been applied more frequently, but it should be acknowledged that physiological data are not necessarily artefacts in specific NF targets or outcome measures. Among these approaches, ‘*regression or subtraction’* of signals from WM, CSF, or control ROIs is simple and feasible for real-time use, but it may remove neural variance; ‘*GSR’* can reduce widespread fluctuations and is particularly relevant for connectivity analyses, yet it remains controversial for introducing artificial correlations and its application for ROI-based or MVPA NF still needs to be tested*; ‘ICA/PCA’* approaches such as CompCor offer data-driven flexibility but may conflate signal and noise; and ‘*whole-brain subtraction’* is straightforward to implement but provides only a crude correction. *‘Post-hoc comparisons’* are useful for checking potential confounds but do not actually denoise the BOLD signal. *‘Instruction’*-based strategies, while not a correction method per se, represent the simplest and least costly way to raise awareness of physiological influences and to prevent NF become a form of ‘expensive breathing training’, though they ultimately rely on participant compliance.

### 4.4 Limitations

A limitation of our literature review is the quantification of extracted features based on whether they were explicitly mentioned in the original study. Therefore, we cannot guarantee that those studies that did not report having used correction methods, indeed, did not use them. It is possible that this was simply not described. As many included studies did not report raw statistics (e.g., means and SDs), we extracted data from figures using WebPlotDigitizer (Rohatg, 2024), which may introduce minor estimation errors despite careful calibration. Additionally, we pooled data across diverse participant populations (e.g., healthy individuals and clinical groups) and target types to ensure sufficient sample size, as the limited number of studies precluded reliable subgroup analyses. However, this may have introduced heterogeneity. We reported the target region types separately in Supplement 1 and provide our raw data in Supplement 2 to facilitate further investigation on this topic. But still, since physiological correction methods are not commonly applied in real-time fMRI studies, the number of studies within each subgroup (based on correction methods) remains quite limited. This substantially constrains the interpretability and generalizability of the subgroup analyses—particularly for categories such as *‘instruction’*, *‘post-hoc comparison’*, and sub-categories for *‘image signal-based’*, each of which includes less than five studies. Such small sample sizes hinder the ability to draw meaningful or robust conclusions from these specific subgroups. Our findings should, therefore, be interpreted as preliminary trends rather than definitive evidence. Lastly, it is important to note that this review investigated the impact of physiological denoising specifically on target brain signal regulation because sufficient data is already available to address this question. What remains open from our analyses is whether the application of physiological correction methods has any impact on the clinical outcomes of rt-fMRI NF studies.

## 5 Conclusion

Our systematic review has shown that physiology correction in rt-fMRI NF studies is far from being standard routine despite its importance for data and training quality. Among the identified approaches, online ‘*imaging signal–based’* correction methods are the most commonly implemented. In addition to these correction methods, it should be considered imperative to acquire simultaneous physiology recordings. Emphasis on this topic should lead to the promotion of rigorous quality control procedures, ultimately enabling neurofeedback applications to exploit their full potential in regulating neural processes.

## Supplementary Material

Supplementary Material 1

Supplementary Material 2

## Data Availability

All data used in this meta-analysis are openly available and can be found in the Supplement 2.
