# Supplementary material for: Physiological artefact control in real-time fMRI neurofeedback – A systematic review and meta-analysis
Source: Imaging Neurosci (Camb). 2026 Apr 13;4:IMAG.a.1215. doi: 10.1162/IMAG.a.1215 (PMC13081740; doi:10.1162/IMAG.a.1215)
Supplement: Supplementary Material 1 [file IMAG.a.1215_supp1.pdf]

# Physiological artefact control in real-time fMRI neurofeedback – a systematic review and meta-analysis

Jingying Zhang[1,2]*, Franziska Weiss[1]*, Peter Kirsch[1,2,3,4], Martin Fungisai Gerchen[1,2,3,4]

[1]Department of Clinical Psychology, Central Institute of Mental Health, University of Heidelberg/Medical Faculty Mannheim, Mannheim, Germany

[2]German Center for Mental Health (DZPG), partner site Mannheim-Heidelberg-Ulm

[3]Department of Psychology, University of Heidelberg, Heidelberg, Germany

[4]Bernstein Center for Computational Neuroscience Heidelberg/Mannheim, Mannheim, Germany

*Equal contribution

Corresponding Author: Jingying Zhang (jingying.zhang@zi-mannheim.de)

**Supplementary Information**

Contents of the Supplementary Materials:

Supplement 1: supplementary figures, notes, results, and all included studies with bibliographic information

Supplement 2: information (basic characteristics, correction methods and extracted results) of all included studies

# Contents

PRISMA flow diagram (Figure S1) .......... 3
Distribution of correction methods (Figure S2) .......... 4
Supplementary Note: Characteristics, Advantages, and Limitations of Physiological Noise Correction Methods .......... 5
Supplementary Results: Group Comparison at the Last Run .......... 7
1. Effect of online physiological artefacts correction .......... 7
1.1 data type: pooled BOLD and FC .......... 7
1.1.1 all target regions (Figure S3) .......... 7
1.1.2 target regions: cortical .......... 9
1.1.3 target regions: subcortical .......... 11
1.2 data type: BOLD .......... 12
1.2.1 all target regions .......... 12
1.2.2 target regions: cortical .......... 15
1.2.3 target regions: subcortical .......... 17
1.3 data type: FC .......... 18
1.3.1 all target regions .......... 18
2. Effect of different online correction methods .......... 21
2.1 data type: BOLD .......... 21
2.1.1 all target regions .......... 21
2.1.2 target regions: cortical .......... 23
2.1.3 target regions: subcortical .......... 25
Included studies .......... 27

## PRISMA flow diagram (Figure S1)

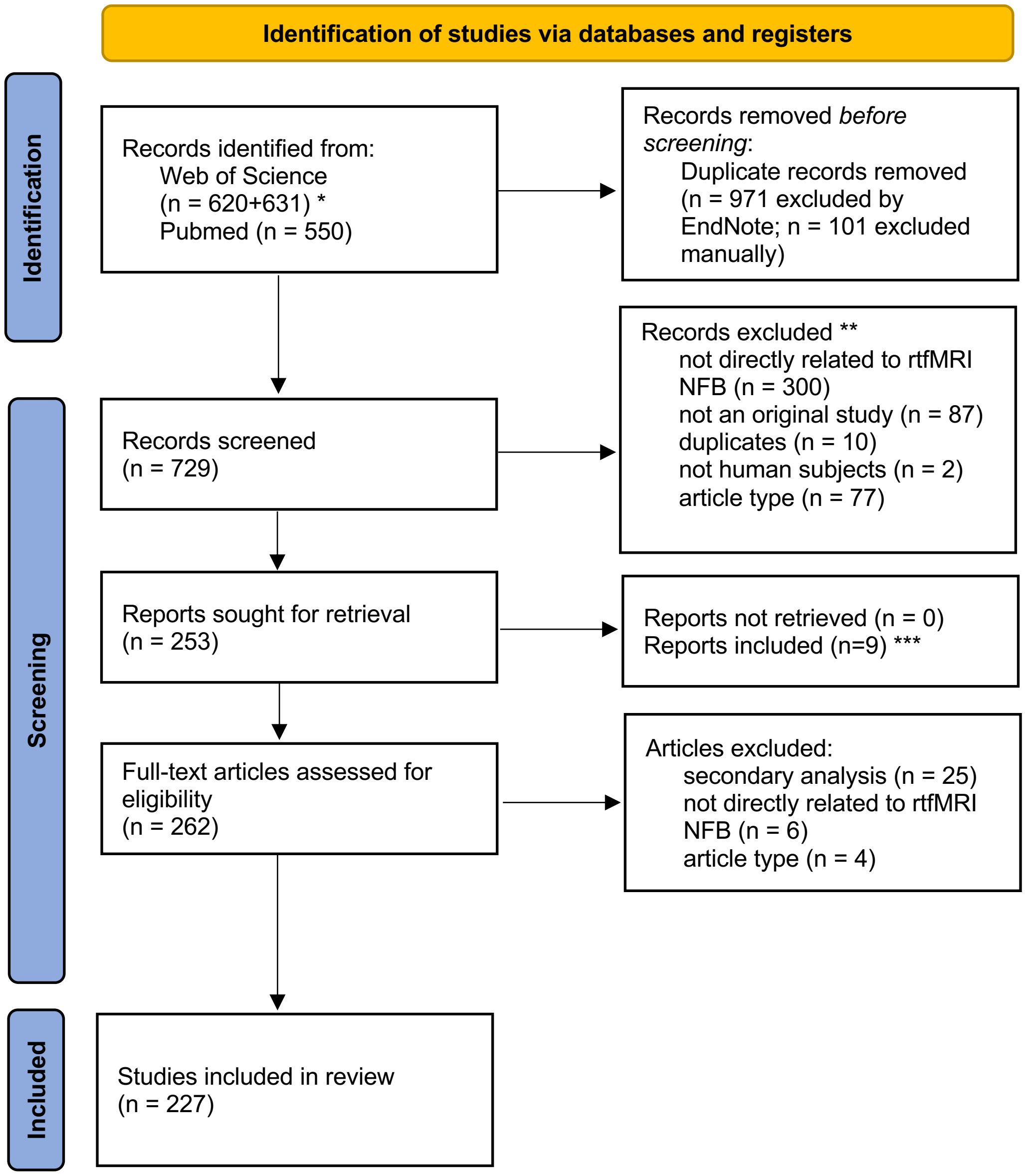


* two authors searched Web of Science and retrieved 620 and 631 records respectively, all of which were imported for screening

** not directly related to rtfMRI NFB: studies using or combining fMRI with other modalities, such as EEG/fNIRS/MEG/VR; studies not conducted in real-time or not providing neurofeedback;

not an original study: reviews, secondary analysis, editorials, annotation, corrections, perspective, opinion, commentary;

article type: methods paper, data paper, case report, preprint, conference paper, brief report, protocol.

*** 9 articles were identified from references during the full-text screening process (8 were from Thibault et al.2018).

*From:* Page MJ, McKenzie JE, Bossuyt PM, Boutron I, Hoffmann TC, Mulrow CD, et al. The PRISMA 2020 statement: an updated guideline for reporting systematic reviews. BMJ 2021;372:n71. doi: 10.1136/bmj.n71

Figure S1. PRISMA flow diagram

## Distribution of correction methods (Figure S2)

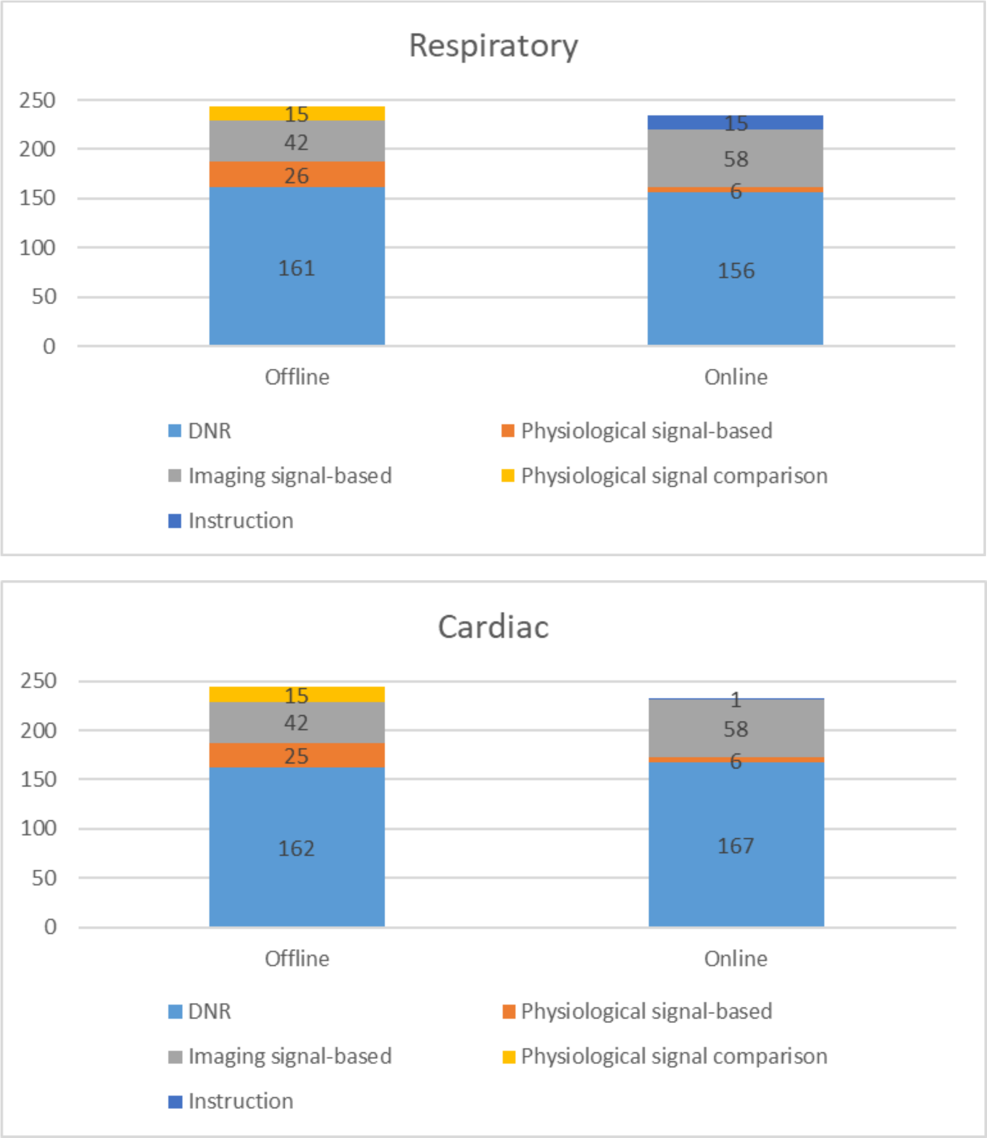


Figure S2. Distribution of correction methods, up: respiratory artefact correction, down: cardiac artefact correction, left: offline, right: online. The absolute number of studies using each method is shown in the respective baskets. Studies that used more than one method were counted for each method.

## **Supplementary Note**: Characteristics, Advantages, and Limitations of Physiological Noise Correction Methods

- 0. Do Not Report (DNR)

This category marks studies where no physiological correction was reported. While it is possible that some studies applied corrections but did not describe them, which limits transparency and reproducibility.

- 1. Physiological Signal–Based methods

These methods (e.g., RETROICOR, PhysIO) use concurrently recorded physiological signals to model and regress out corresponding fluctuations in the BOLD signal.

Advantages: Physiologically grounded; effective in removing structured artefacts.

Limitations: Require synchronized high-quality recordings and additional computational resources, which may complicate real-time implementation.

- 2. Imaging Signal–Based methods

These approaches do not rely on external physiological recordings but instead use imaging data to estimate nuisance variance.

  - 2.1 Regression/Subtraction: signals from WM, CSF, or control ROIs are regressed out or subtracted.

    Advantages: Simple and feasible for real-time use.

    Limitations: Risk of removing genuine neural variance.

  - 2.2 Global Signal Regression (GSR): reducing global sources of noise from respiration or motion-related fluctuations by removing the average BOLD time series across the whole brain (either computed as the mean across all voxels or voxel-wise regression against the global signal) from each voxel's time series.

    Advantages: Effective in reducing global physiological fluctuations, especially in connectivity analyses.

    Limitations: Controversial, as it can introduce artificial anti-correlations and distort network metrics; unclear effect on ROI-based or MVPA-based NF.

  - 2.3 ICA/PCA (e.g., CompCor): extracts independent or principal components (often from WM or CSF) to capture structured noise.

    Advantages: Data-driven; flexible; widely used in offline pipelines (e.g., fMRIPrep, CONN).

    Limitations: Requires sufficient data for decomposition; components may not represent pure noise and can include neural signal.

- 2.4 Whole-Brain Subtraction: subtracts or normalizes ROI signals against the mean signal of the whole brain, but without performing voxel-wise regression.

  Advantages: Straightforward and easy to implement online.

  Limitations: Crude correction; non-specific to physiological artefacts.

- 3. Post-hoc Physiological Comparisons

These method includes studies that examined respiration rate and/or heart rate across groups or conditions to rule out confounding, but did not directly correct the fMRI data.

Advantages: Useful to rule out confounds without altering the NF pipeline.

Limitations: Does not correct the BOLD signal; residual artefacts remain in the NF measure.

- 4. Instruction-Based Strategies

Participants are explicitly instructed not to use breathing strategies to alter NF signals.

Advantages: Simple, inexpensive, easily integrated into training protocols; raises awareness of physiological confounds and helps prevent NF turning into a form of expensive breathing regulation training.

Limitations: Does not directly remove artefacts; relies on participant compliance and cannot fully prevent physiological confounds.

## **Supplementary Results**: Group Comparison at the Last Run

### 1. Effect of online physiological artefacts correction

### 1.1 data type: pooled BOLD and FC

#### 1.1.1 all target regions (Figure S3)

N=55

Effect size of each study
Estimated overall effect size
No-effect value
Confidence interval of effect size
Overall effect size value
Estimated overall confidence interval

| Online | ID | Study | Effect Size | Std. Error | Lower | Upper | p-value | Weight | Weight (%) |
|---|---|---|---|---|---|---|---|---|---|
| DNR | 8 | Zhao et al.,2023 | 0.58 | 0.41 | -0.23 | 1.39 | 0.16 | 4.45 | 1.84 |
| | 14 | Zahn et al.,2019 | 0.27 | 0.38 | -0.47 | 1.01 | 0.48 | 5.00 | 2.06 |
| | 23 | Hohenfeld et al.,2017 | 1.53 | 0.61 | 0.34 | 2.73 | 0.01 | 2.34 | 0.97 |
| | 41 | Harmelech et al.,2015 | 0.94 | 0.53 | -0.09 | 1.98 | 0.07 | 3.00 | 1.24 |
| | 48 | Paret et al.,2014 | -0.12 | 0.35 | -0.81 | 0.57 | 0.73 | 5.52 | 2.28 |
| | 59 | Liu et al.,2020 | 0.83 | 0.38 | 0.08 | 1.58 | 0.03 | 4.98 | 2.06 |
| | 63 | Sarkheil et al.,2015 | 1.46 | 0.61 | 0.27 | 2.65 | 0.02 | 2.36 | 0.97 |
| | 65 | Sampaio-Baptista et al... | 1.28 | 0.49 | 0.32 | 2.24 | 0.01 | 3.37 | 1.39 |
| | 68 | Zilverstand et al.,2017 | -0.13 | 0.56 | -1.23 | 0.96 | 0.81 | 2.73 | 1.13 |
| | 70 | Zhang et al.,2013 | 0.89 | 0.41 | 0.08 | 1.70 | 0.03 | 4.43 | 1.83 |
| | 75 | Yoo et al.,2007 | 0.28 | 0.41 | -0.52 | 1.09 | 0.49 | 4.46 | 1.84 |
| | 77 | Megumi et al.,2015 | 0.84 | 0.43 | 0.01 | 1.67 | 0.05 | 4.22 | 1.74 |
| | 93 | Yoo et al.,2006 | 0.87 | 0.45 | -0.00 | 1.75 | 0.05 | 3.92 | 1.62 |
| | 94 | Neyedli et al.,2018 | 0.56 | 0.41 | -0.24 | 1.36 | 0.17 | 4.50 | 1.86 |
| | 96 | Hartwell et al.,2016 | 0.49 | 0.31 | -0.11 | 1.09 | 0.11 | 6.68 | 2.76 |
| | 99 | Johnson et al.,2012 | 1.46 | 0.44 | 0.59 | 2.32 | 0.00 | 3.99 | 1.65 |
| | 100 | Hellrung et al.,2018 | 0.90 | 0.44 | 0.04 | 1.77 | 0.04 | 3.97 | 1.64 |
| | 106 | Zhang et al.,2023 | 0.41 | 0.27 | -0.12 | 0.93 | 0.13 | 7.84 | 3.24 |
| | 114 | Garrison et al.,2021 | 0.30 | 0.32 | -0.33 | 0.94 | 0.35 | 6.24 | 2.58 |
| | 128 | Yoo et al.,2008 | 0.41 | 0.43 | -0.44 | 1.25 | 0.35 | 4.14 | 1.71 |
| | 129 | Rance et al.,2023 | -0.43 | 0.34 | -1.10 | 0.24 | 0.20 | 5.78 | 2.39 |
| | 138 | Pereira et al.,2023 | 1.09 | 0.43 | 0.26 | 1.93 | 0.01 | 4.22 | 1.74 |
| | 144 | Scheinost et al.,2013 | 0.53 | 0.46 | -0.36 | 1.42 | 0.24 | 3.80 | 1.57 |
| | 152 | Sukhodolsky et al.,2020 | 0.92 | 0.46 | 0.02 | 1.82 | 0.05 | 3.75 | 1.55 |
| | 175 | Kirsch et al.,2016 | 1.14 | 0.42 | 0.31 | 1.97 | 0.01 | 4.26 | 1.76 |
| | 190 | Sherwood et al.,2019 | 0.21 | 0.41 | -0.59 | 1.02 | 0.60 | 4.48 | 1.85 |
| | 197 | Guan et al.,2015 | 1.83 | 0.64 | 0.57 | 3.09 | 0.00 | 2.14 | 0.88 |
| | 199 | Sreedharan et al.,2020 | -0.02 | 0.71 | -1.41 | 1.36 | 0.98 | 1.80 | 0.74 |
| | 217 | Direito et al.,2019 | 0.28 | 0.47 | -0.64 | 1.19 | 0.56 | 3.63 | 1.50 |
| | 220 | Chung et al.,2023 | 0.17 | 0.30 | -0.42 | 0.77 | 0.56 | 6.80 | 2.80 |
| | 225 | Herwig et al.,2019 | 1.12 | 0.43 | 0.28 | 1.96 | 0.01 | 4.21 | 1.74 |
| | 238 | Andersson et al.,2019 | 0.73 | 0.48 | -0.21 | 1.66 | 0.13 | 3.54 | 1.46 |
| | 242 | Sherwood et al.,2018 | 0.87 | 0.43 | 0.04 | 1.71 | 0.04 | 4.22 | 1.74 |
| | 247 | Yao et al.,2016 | 0.76 | 0.37 | 0.03 | 1.49 | 0.04 | 5.19 | 2.14 |
| | 253 | Bray S et al.,2007 | 1.13 | 0.54 | 0.07 | 2.19 | 0.04 | 2.86 | 1.18 |
| | 254 | Caria et al.,2010 | 1.89 | 0.57 | 0.78 | 3.00 | 0.00 | 2.65 | 1.09 |
| | 258 | McCaig et al.,2011 | 1.30 | 0.45 | 0.42 | 2.18 | 0.00 | 3.88 | 1.60 |
| | | Subgroup Overall | 0.67 | 0.08 | 0.51 | 0.83 | 0.00 | | |
| corrected | 3 | Papoutsi et al.,2020 | 0.82 | 0.30 | 0.22 | 1.41 | 0.01 | 6.76 | 2.79 |
| | 29 | Scheinost et al.,2020 | 0.43 | 0.50 | -0.55 | 1.41 | 0.39 | 3.27 | 1.35 |
| | 55 | Kanel et al.,2019 | 1.22 | 0.49 | 0.26 | 2.17 | 0.01 | 3.41 | 1.41 |
| | 62 | Weiss et al.,2022 | 0.54 | 0.34 | -0.14 | 1.21 | 0.12 | 5.74 | 2.37 |
| | 88 | Li et al.,2018 | 0.20 | 0.39 | -0.56 | 0.96 | 0.61 | 4.81 | 1.98 |
| | 91 | Scharnowski et al.,2012 | 1.48 | 0.60 | 0.30 | 2.66 | 0.01 | 2.40 | 0.99 |
| | 103 | Weiss et al.,2020 | 0.30 | 0.35 | -0.38 | 0.99 | 0.39 | 5.60 | 2.31 |
| | 112 | Kim et al.,2019 | 0.41 | 0.28 | -0.14 | 0.96 | 0.14 | 7.44 | 3.07 |
| | 117 | Hui et al.,2014 | 0.87 | 0.43 | 0.04 | 1.71 | 0.04 | 4.19 | 1.73 |
| | 140 | Sulzer et al.,2013 | 0.11 | 0.35 | -0.58 | 0.81 | 0.76 | 5.51 | 2.27 |
| | 149 | Tsuchiyagaito et al.,2021 | 0.81 | 0.39 | 0.04 | 1.58 | 0.04 | 4.75 | 1.96 |
| | 160 | Tsuchiyagaito et al.,2023 | 0.30 | 0.32 | -0.33 | 0.93 | 0.36 | 6.26 | 2.59 |
| | 169 | Kohl et al.,2019 | 0.13 | 0.35 | -0.56 | 0.81 | 0.72 | 5.61 | 2.31 |
| | 184 | Caria et al.,2007 | 0.35 | 0.67 | -0.97 | 1.66 | 0.61 | 1.98 | 0.82 |
| | 203 | Lawrence et al.,2014 | 0.66 | 0.44 | -0.21 | 1.53 | 0.14 | 3.96 | 1.63 |
| | 251 | Zhang et al.2013 | 0.91 | 0.38 | 0.16 | 1.66 | 0.02 | 4.92 | 2.03 |
| | 255 | deCharms et al.,2004 | 1.49 | 0.33 | 0.84 | 2.14 | 0.00 | 6.07 | 2.50 |
| | 261 | Zhao X et al.,2013 | 0.71 | 0.42 | -0.12 | 1.53 | 0.09 | 4.29 | 1.77 |
| | | Subgroup Overall | 0.62 | 0.10 | 0.42 | 0.82 | 0.00 | | |
| Overall | | | 0.65 | 0.06 | 0.52 | 0.77 | 0.00 | | |

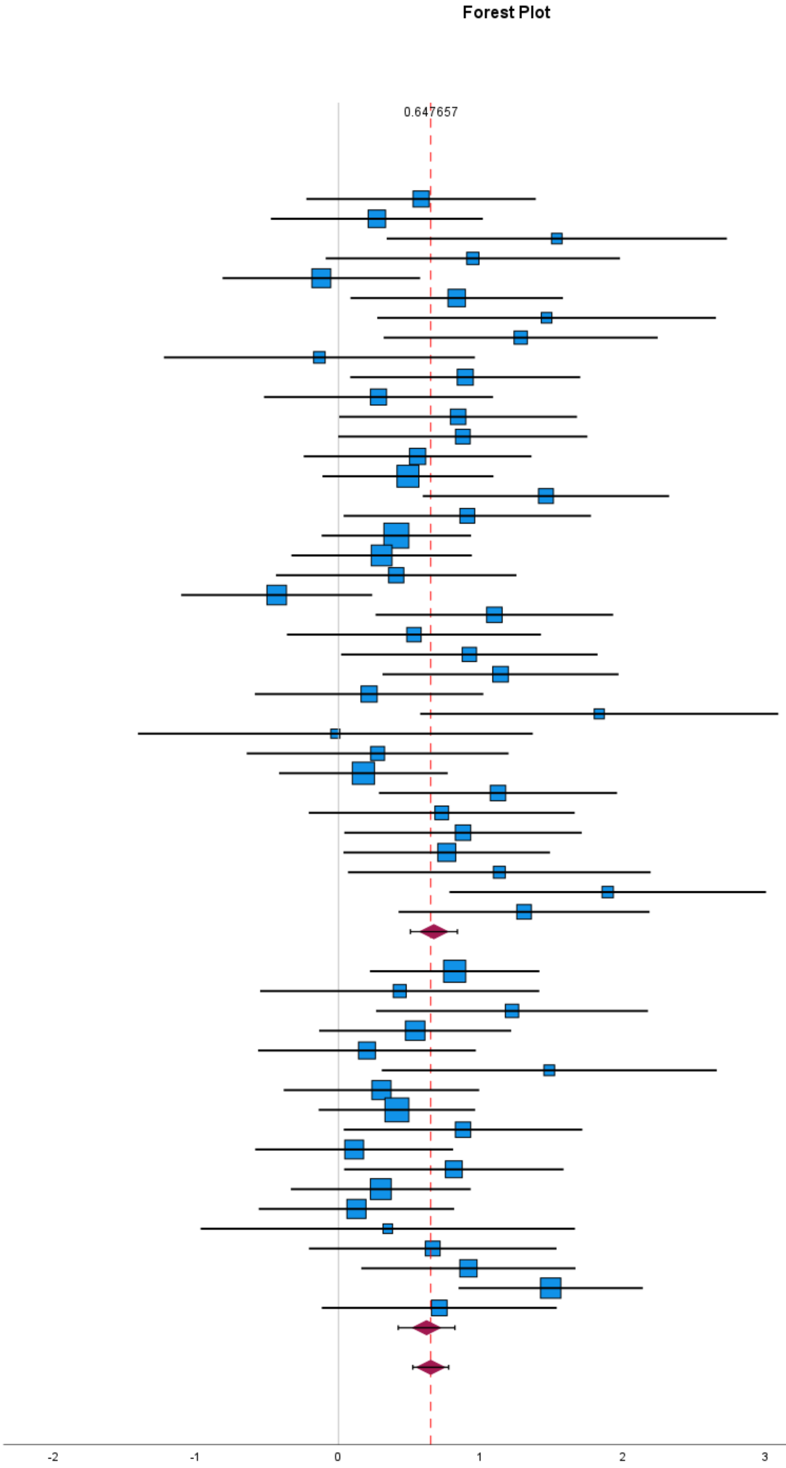


Model: Random-effects model
Heterogeneity: Tau-squared = 0.06, H-squared = 1.34, I-squared = 0.25
Homogeneity: Q = 72.52, df = 54, p-value = 0.05
Test of overall effect size: z = 10.08, p-value = 0.00
Test of between-subgroup homogeneity: Q = 0.15, df = 1, p-value = 0.70

Figure S3. Forest plot of reported between-group effect sizes pooling BOLD activation and FC measures

## Effect Size Estimates for Subgroup Analysis

| | Effect Size | Std. Error | Z | Sig. (2-tailed) | 95% Confidence Interval Lower | 95% Confidence Interval Upper |
|---|---|---|---|---|---|---|
| DNR | .670 | .0842 | 7.962 | <.001 | .505 | .835 |
| corrected | .618 | .1015 | 6.094 | <.001 | .419 | .817 |
| Overall | .648 | .0642 | 10.082 | <.001 | .522 | .774 |

## Egger's Regression-Based Test[a]

| | Parameter | Coefficient | Std. Error | t | Sig. (2-tailed) | 95% Confidence Interval Lower | 95% Confidence Interval Upper |
|---|---|---|---|---|---|---|---|
| DNR | (Intercept) | -.631 | .3370 | -1.874 | .069 | -1.316 | .053 |
| | SE[b] | 3.093 | .8047 | 3.844 | <.001 | 1.460 | 4.727 |
| corrected | (Intercept) | .193 | .4922 | .392 | .700 | -.851 | 1.236 |
| | SE[b] | 1.126 | 1.2735 | .884 | .390 | -1.574 | 3.826 |
| Overall | (Intercept) | -.314 | .2779 | -1.129 | .264 | -.871 | .244 |
| | SE[b] | 2.376 | .6802 | 3.493 | <.001 | 1.012 | 3.741 |

a. Random-effects meta-regression
b. Standard error of effect size

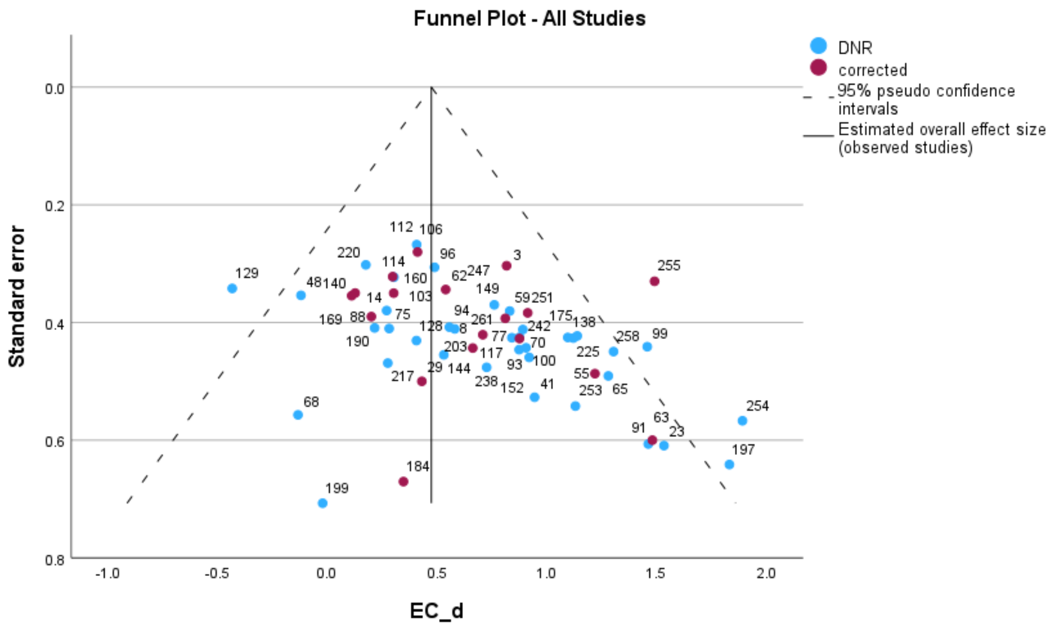

### 1.1.2 target regions: cortical

n=45

**Effect Size Estimates for Subgroup Analysis**

| | Effect Size | Std. Error | Z | Sig. (2-tailed) | 95% Confidence Interval Lower | 95% Confidence Interval Upper |
|---|---|---|---|---|---|---|
| DNR | .689 | .0953 | 7.227 | <.001 | .502 | .876 |
| corrected | .682 | .1115 | 6.121 | <.001 | .464 | .901 |
| Overall | .683 | .0717 | 9.525 | <.001 | .542 | .823 |

**Egger's Regression-Based Test[a]**

| | Parameter | Coefficient | Std. Error | t | Sig. (2-tailed) | 95% Confidence Interval Lower | 95% Confidence Interval Upper |
|---|---|---|---|---|---|---|---|
| DNR | (Intercept) | -.509 | .3768 | -1.351 | .187 | -1.281 | .263 |
| | SE[b] | 2.781 | .8750 | 3.178 | .004 | .988 | 4.573 |
| corrected | (Intercept) | .272 | .4998 | .545 | .595 | -.807 | 1.352 |
| | SE[b] | 1.091 | 1.2972 | .841 | .415 | -1.711 | 3.894 |
| Overall | (Intercept) | -.153 | .2954 | -.517 | .608 | -.748 | .443 |
| | SE[b] | 2.041 | .7123 | 2.865 | .006 | .604 | 3.477 |

a. Random-effects meta-regression

b. Standard error of effect size

**Forest Plot**

Effect size of each study
Estimated overall effect size
No-effect value
Confidence interval of effect size
Overall effect size value
Estimated overall confidence interval

| Online | ID | Study | Effect Size | Std. Error | Lower | Upper | p-value | Weight | Weight (%) |
|---|---|---|---|---|---|---|---|---|---|
| DNR | 14 | Zahn et al.,2019 | 0.27 | 0.38 | -0.47 | 1.01 | 0.48 | 5.02 | 2.58 |
| | 23 | Hohenfeld et al.,2017 | 1.53 | 0.61 | 0.34 | 2.73 | 0.01 | 2.35 | 1.20 |
| | 41 | Harmelech et al.,2015 | 0.94 | 0.53 | -0.09 | 1.98 | 0.07 | 3.01 | 1.54 |
| | 63 | Sarkheil et al.,2015 | 1.46 | 0.61 | 0.27 | 2.65 | 0.02 | 2.36 | 1.21 |
| | 65 | Sampaio-Baptista et al... | 1.28 | 0.49 | 0.32 | 2.24 | 0.01 | 3.38 | 1.74 |
| | 68 | Zilverstand et al.,2017 | -0.13 | 0.56 | -1.23 | 0.96 | 0.81 | 2.74 | 1.41 |
| | 70 | Zhang et al.,2013 | 0.89 | 0.41 | 0.08 | 1.70 | 0.03 | 4.45 | 2.29 |
| | 75 | Yoo et al.,2007 | 0.28 | 0.41 | -0.52 | 1.09 | 0.49 | 4.48 | 2.30 |
| | 77 | Megumi et al.,2015 | 0.84 | 0.43 | 0.01 | 1.67 | 0.05 | 4.23 | 2.17 |
| | 93 | Yoo et al.,2006 | 0.87 | 0.45 | -0.00 | 1.75 | 0.05 | 3.94 | 2.02 |
| | 94 | Neyedli et al.,2018 | 0.56 | 0.41 | -0.24 | 1.36 | 0.17 | 4.52 | 2.32 |
| | 96 | Hartwell et al.,2016 | 0.49 | 0.31 | -0.11 | 1.09 | 0.11 | 6.72 | 3.45 |
| | 99 | Johnson et al.,2012 | 1.46 | 0.44 | 0.59 | 2.32 | 0.00 | 4.01 | 2.06 |
| | 106 | Zhang et al.,2023 | 0.41 | 0.27 | -0.12 | 0.93 | 0.13 | 7.90 | 4.06 |
| | 114 | Garrison et al.,2021 | 0.30 | 0.32 | -0.33 | 0.94 | 0.35 | 6.28 | 3.22 |
| | 128 | Yoo et al.,2008 | 0.41 | 0.43 | -0.44 | 1.25 | 0.35 | 4.16 | 2.14 |
| | 129 | Rance et al.,2023 | -0.43 | 0.34 | -1.10 | 0.24 | 0.20 | 5.81 | 2.99 |
| | 138 | Pereira et al.,2023 | 1.09 | 0.43 | 0.26 | 1.93 | 0.01 | 4.24 | 2.18 |
| | 144 | Scheinost et al.,2013 | 0.53 | 0.46 | -0.36 | 1.42 | 0.24 | 3.82 | 1.96 |
| | 152 | Sukhodolsky et al.,2020 | 0.92 | 0.46 | 0.02 | 1.82 | 0.05 | 3.76 | 1.93 |
| | 190 | Sherwood et al.,2019 | 0.21 | 0.41 | -0.59 | 1.02 | 0.60 | 4.50 | 2.31 |
| | 197 | Guan et al.,2015 | 1.83 | 0.64 | 0.57 | 3.09 | 0.00 | 2.14 | 1.10 |
| | 199 | Sreedharan et al.,2020 | -0.02 | 0.71 | -1.41 | 1.36 | 0.98 | 1.80 | 0.93 |
| | 217 | Direito et al.,2019 | 0.28 | 0.47 | -0.64 | 1.19 | 0.56 | 3.64 | 1.87 |
| | 238 | Andersson et al.,2019 | 0.73 | 0.48 | -0.21 | 1.66 | 0.13 | 3.55 | 1.82 |
| | 242 | Sherwood et al.,2018 | 0.87 | 0.43 | 0.04 | 1.71 | 0.04 | 4.24 | 2.18 |
| | 247 | Yao et al.,2016 | 0.76 | 0.37 | 0.03 | 1.49 | 0.04 | 5.21 | 2.68 |
| | 253 | Bray S et al.,2007 | 1.13 | 0.54 | 0.07 | 2.19 | 0.04 | 2.87 | 1.47 |
| | 254 | Caria et al.,2010 | 1.89 | 0.57 | 0.78 | 3.00 | 0.00 | 2.66 | 1.36 |
| | 258 | McCaig et al.,2011 | 1.30 | 0.45 | 0.42 | 2.18 | 0.00 | 3.89 | 2.00 |
| | | Subgroup Overall | 0.69 | 0.10 | 0.50 | 0.88 | 0.00 | | |
| corrected | 3 | Papoutsi et al.,2020 | 0.82 | 0.30 | 0.22 | 1.41 | 0.01 | 6.80 | 3.49 |
| | 29 | Scheinost et al.,2020 | 0.43 | 0.50 | -0.55 | 1.41 | 0.39 | 3.28 | 1.68 |
| | 55 | Kanel et al.,2019 | 1.22 | 0.49 | 0.26 | 2.17 | 0.01 | 3.42 | 1.76 |
| | 91 | Scharnowski et al.,2012 | 1.48 | 0.60 | 0.30 | 2.66 | 0.01 | 2.41 | 1.24 |
| | 112 | Kim et al.,2019 | 0.41 | 0.28 | -0.14 | 0.96 | 0.14 | 7.49 | 3.85 |
| | 117 | Hui et al.,2014 | 0.87 | 0.43 | 0.04 | 1.71 | 0.04 | 4.21 | 2.16 |
| | 149 | Tsuchiyagaito et al.,2021 | 0.81 | 0.39 | 0.04 | 1.58 | 0.04 | 4.78 | 2.45 |
| | 160 | Tsuchiyagaito et al.,2023 | 0.30 | 0.32 | -0.33 | 0.93 | 0.36 | 6.30 | 3.24 |
| | 169 | Kohl et al.,2019 | 0.13 | 0.35 | -0.56 | 0.81 | 0.72 | 5.64 | 2.89 |
| | 184 | Caria et al.,2007 | 0.35 | 0.67 | -0.97 | 1.66 | 0.61 | 1.98 | 1.02 |
| | 251 | Zhang et al.2013 | 0.91 | 0.38 | 0.16 | 1.66 | 0.02 | 4.95 | 2.54 |
| | 255 | deCharms et al.,2004 | 1.49 | 0.33 | 0.84 | 2.14 | 0.00 | 6.10 | 3.13 |
| | 261 | Zhao X et al.,2013 | 0.71 | 0.42 | -0.12 | 1.53 | 0.09 | 4.31 | 2.21 |
| | 62 | Weiss et al.,2022 | 0.54 | 0.34 | -0.14 | 1.21 | 0.12 | 5.77 | 2.96 |
| | 103 | Weiss et al.,2020 | 0.30 | 0.35 | -0.38 | 0.99 | 0.39 | 5.63 | 2.89 |
| | | Subgroup Overall | 0.68 | 0.11 | 0.46 | 0.90 | 0.00 | | |
| Overall | | | 0.68 | 0.07 | 0.54 | 0.82 | 0.00 | | |

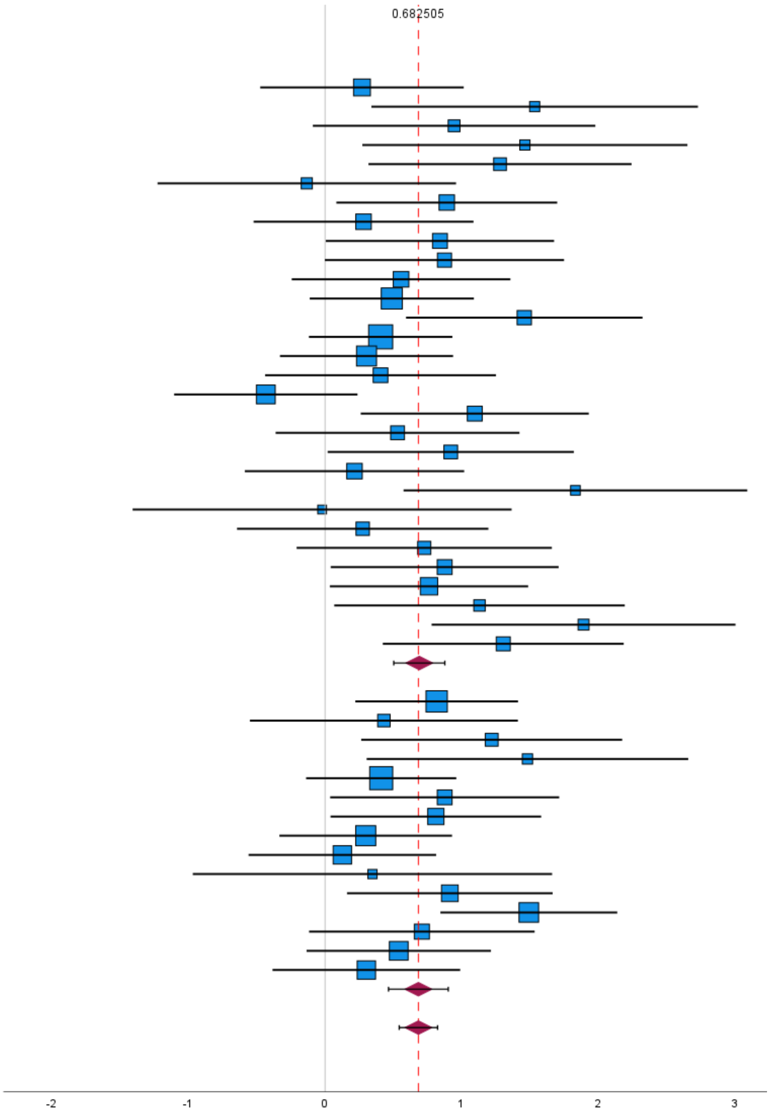


Model: Random-effects model
Heterogeneity: Tau-squared = 0.05, H-squared = 1.33, I-squared = 0.25
Homogeneity: Q = 58.69, df = 44, p-value = 0.07
Test of overall effect size: z = 9.52, p-value = 0.00
Test of between-subgroup homogeneity: Q = 0.00, df = 1, p-value = 0.96

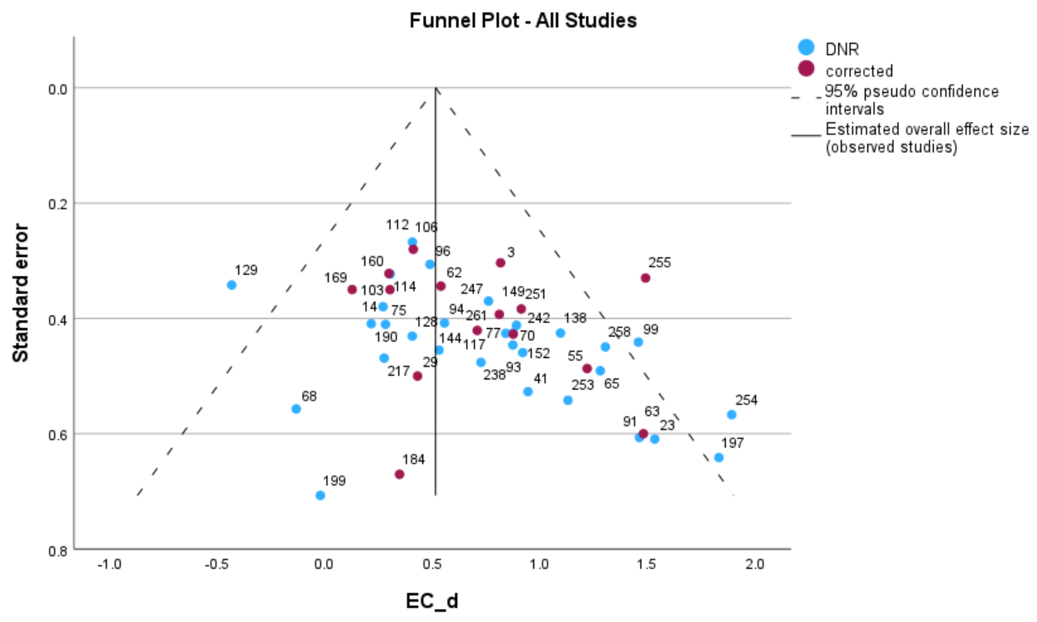


### 1.1.3 target regions: subcortical

n=12

**Effect Size Estimates for Subgroup Analysis**

| | Effect Size | Std. Error | Z | Sig. (2-tailed) | 95% Confidence Interval Lower | Upper |
|---|---|---|---|---|---|---|
| DNR | .610 | .1907 | 3.198 | .001 | .236 | .983 |
| corrected | .347 | .1661 | 2.089 | .037 | .021 | .673 |
| Overall | .480 | .1157 | 4.149 | <.001 | .253 | .707 |

**Egger's Regression-Based Test[a]**

| | Parameter | Coefficient | Std. Error | t | Sig. (2-tailed) | 95% Confidence Interval Lower | Upper |
|---|---|---|---|---|---|---|---|
| DNR | (Intercept) | -2.173 | 1.1032 | -1.970 | .106 | -5.009 | .663 |
| | SE[b] | 7.238 | 2.8876 | 2.506 | .054 | -.185 | 14.660 |
| corrected | (Intercept) | -.492 | 1.8372 | -.268 | .806 | -6.339 | 5.355 |
| | SE[b] | 2.267 | 4.9452 | .458 | .678 | -13.471 | 18.004 |
| Overall | (Intercept) | -1.838 | .9369 | -1.961 | .078 | -3.925 | .250 |
| | SE[b] | 6.162 | 2.4814 | 2.483 | .032 | .633 | 11.691 |

a. Random-effects meta-regression

b. Standard error of effect size

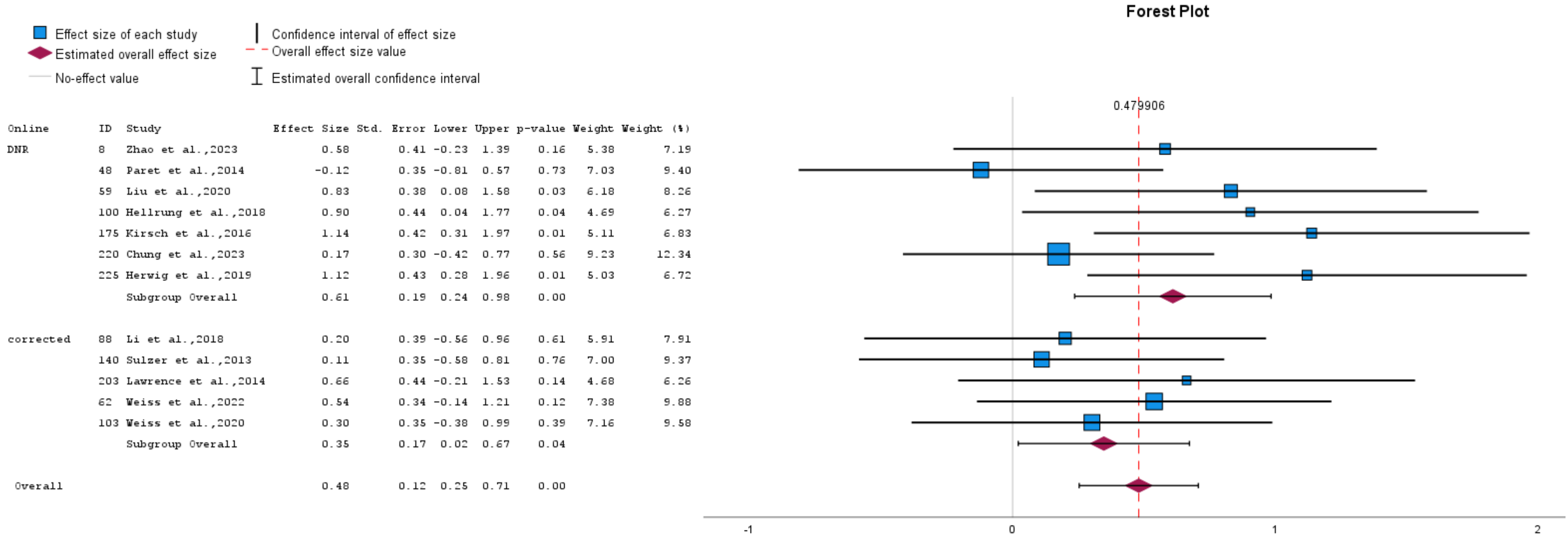


| Online | ID | Study | Effect Size | Std. Error | Lower | Upper | p-value | Weight | Weight (%) |
|---|---|---|---|---|---|---|---|---|---|
| DNR | 8 | Zhao et al.,2023 | 0.58 | 0.41 | -0.23 | 1.39 | 0.16 | 5.38 | 7.19 |
| | 48 | Paret et al.,2014 | -0.12 | 0.35 | -0.81 | 0.57 | 0.73 | 7.03 | 9.40 |
| | 59 | Liu et al.,2020 | 0.83 | 0.38 | 0.08 | 1.58 | 0.03 | 6.18 | 8.26 |
| | 100 | Hellrung et al.,2018 | 0.90 | 0.44 | 0.04 | 1.77 | 0.04 | 4.69 | 6.27 |
| | 175 | Kirsch et al.,2016 | 1.14 | 0.42 | 0.31 | 1.97 | 0.01 | 5.11 | 6.83 |
| | 220 | Chung et al.,2023 | 0.17 | 0.30 | -0.42 | 0.77 | 0.56 | 9.23 | 12.34 |
| | 225 | Herwig et al.,2019 | 1.12 | 0.43 | 0.28 | 1.96 | 0.01 | 5.03 | 6.72 |
| | | Subgroup Overall | 0.61 | 0.19 | 0.24 | 0.98 | 0.00 | | |
| corrected | 88 | Li et al.,2018 | 0.20 | 0.39 | -0.56 | 0.96 | 0.61 | 5.91 | 7.91 |
| | 140 | Sulzer et al.,2013 | 0.11 | 0.35 | -0.58 | 0.81 | 0.76 | 7.00 | 9.37 |
| | 203 | Lawrence et al.,2014 | 0.66 | 0.44 | -0.21 | 1.53 | 0.14 | 4.68 | 6.26 |
| | 62 | Weiss et al.,2022 | 0.54 | 0.34 | -0.14 | 1.21 | 0.12 | 7.38 | 9.88 |
| | 103 | Weiss et al.,2020 | 0.30 | 0.35 | -0.38 | 0.99 | 0.39 | 7.16 | 9.58 |
| | | Subgroup Overall | 0.35 | 0.17 | 0.02 | 0.67 | 0.04 | | |
| Overall | | | 0.48 | 0.12 | 0.25 | 0.71 | 0.00 | | |

Model: Random-effects model
Heterogeneity: Tau-squared = 0.02, H-squared = 1.12, I-squared = 0.11
Homogeneity: Q = 12.45, df = 11, p-value = 0.33
Test of overall effect size: z = 4.15, p-value = 0.00
Test of between-subgroup homogeneity: Q = 1.08, df = 1, p-value = 0.30

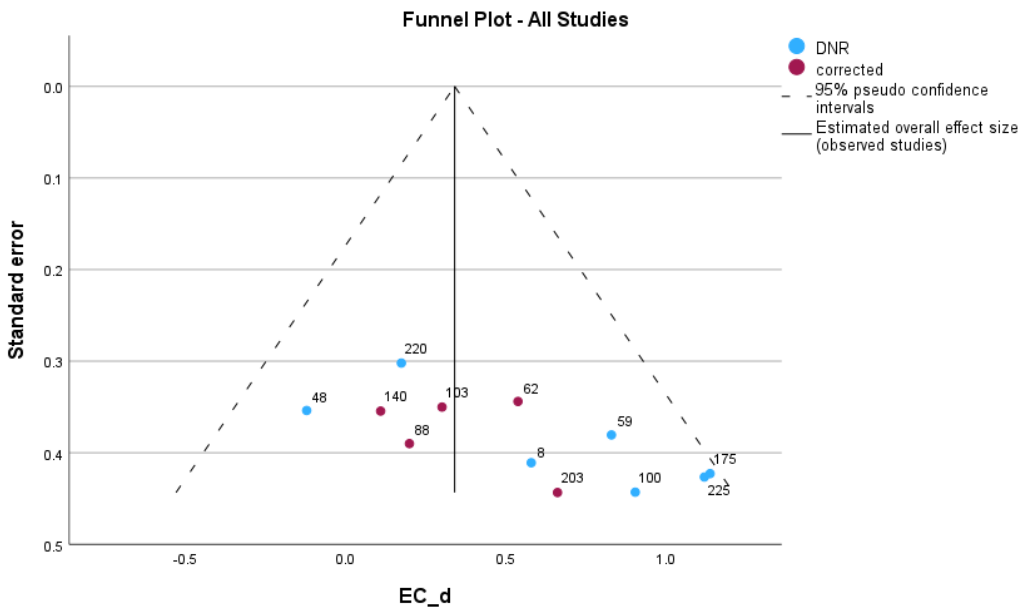


## 1.2 data type: BOLD

### 1.2.1 all target regions

n=47

**Effect Size Estimates for Subgroup Analysis**

| | Effect Size | Std. Error | Z | Sig. (2-tailed) | 95% Confidence Interval Lower | 95% Confidence Interval Upper |
|---|---|---|---|---|---|---|
| DNR | .682 | .0886 | 7.698 | <.001 | .508 | .855 |
| corrected | .726 | .1475 | 4.924 | <.001 | .437 | 1.015 |

| Overall | .693 | .0753 | 9.204 | <.001 | .545 | .840 |
|---|---|---|---|---|---|---|

**Egger's Regression-Based Test[a]**

| | Parameter | Coefficient | Std. Error | t | Sig. (2-tailed) | 95% Confidence Interval | |
|---|---|---|---|---|---|---|---|
| | | | | | | Lower | Upper |
| DNR | (Intercept) | -.605 | .3476 | -1.741 | .091 | -1.312 | .102 |
| | SE[b] | 3.043 | .8248 | 3.689 | <.001 | 1.364 | 4.721 |
| corrected | (Intercept) | .448 | .7085 | .633 | .541 | -1.131 | 2.027 |
| | SE[b] | .690 | 1.7151 | .403 | .696 | -3.131 | 4.512 |
| Overall | (Intercept) | -.289 | .3290 | -.879 | .384 | -.952 | .373 |
| | SE[b] | 2.355 | .7796 | 3.021 | .004 | .785 | 3.925 |

a. Random-effects meta-regression

b. Standard error of effect size

■ Effect size of each study | Confidence interval of effect size
◆ Estimated overall effect size -- Overall effect size value
— No-effect value I Estimated overall confidence interval

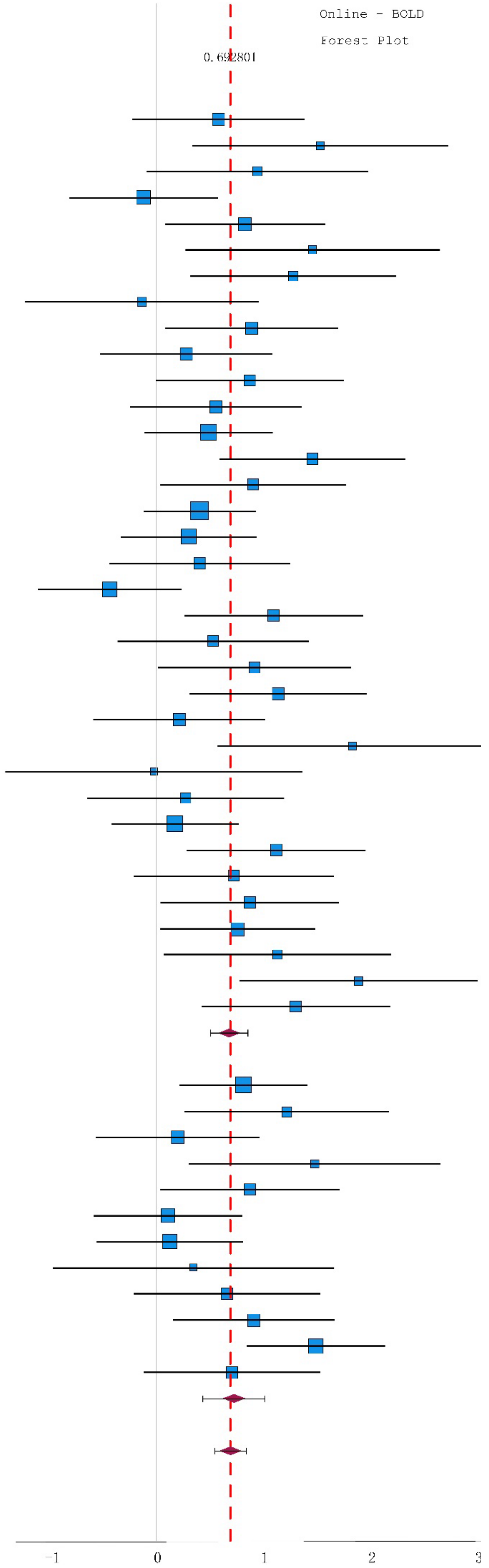


| ID | Study | Effect Size | SE | Lower | Upper | p-value | Weight | (%) |
|---|---|---|---|---|---|---|---|---|
| DNR | | | | | | | | |
| 8 | Zhao et al.,2023 | 0.58 | 0.41 | -0.23 | 1.39 | 0.16 | 3.95 | 2.24 |
| 23 | Hohenfeld et al.,2017 | 1.53 | 0.61 | 0.34 | 2.73 | 0.01 | 2.19 | 1.24 |
| 41 | Harmelech et al.,2015 | 0.94 | 0.53 | -0.09 | 1.98 | 0.07 | 2.76 | 1.56 |
| 48 | Paret et al.,2014 | -0.12 | 0.35 | -0.81 | 0.57 | 0.73 | 4.77 | 2.70 |
| 59 | Liu et al.,2020 | 0.83 | 0.38 | 0.08 | 1.58 | 0.03 | 4.36 | 2.47 |
| 63 | Sarkheil et al.,2015 | 1.46 | 0.61 | 0.27 | 2.65 | 0.02 | 2.21 | 1.25 |
| 65 | Sampaio-Baptista et al... | 1.28 | 0.49 | 0.32 | 2.24 | 0.01 | 3.07 | 1.74 |
| 68 | Zilverstand et al.,2017 | -0.13 | 0.56 | -1.23 | 0.96 | 0.81 | 2.53 | 1.43 |
| 70 | Zhang et al.,2013 | 0.89 | 0.41 | 0.08 | 1.70 | 0.03 | 3.93 | 2.23 |
| 75 | Yoo et al.,2007 | 0.28 | 0.41 | -0.52 | 1.09 | 0.49 | 3.95 | 2.24 |
| 93 | Yoo et al.,2006 | 0.87 | 0.45 | -0.00 | 1.75 | 0.05 | 3.52 | 2.00 |
| 94 | Neyedli et al.,2018 | 0.56 | 0.41 | -0.24 | 1.36 | 0.17 | 3.98 | 2.26 |
| 96 | Hartwell et al.,2016 | 0.49 | 0.31 | -0.11 | 1.09 | 0.11 | 5.61 | 3.18 |
| 99 | Johnson et al.,2012 | 1.46 | 0.44 | 0.59 | 2.32 | 0.00 | 3.58 | 2.03 |
| 100 | Hellrung et al.,2018 | 0.90 | 0.44 | 0.04 | 1.77 | 0.04 | 3.56 | 2.02 |
| 106 | Zhang et al.,2023 | 0.41 | 0.27 | -0.12 | 0.93 | 0.13 | 6.40 | 3.63 |
| 114 | Garrison et al.,2021 | 0.30 | 0.32 | -0.33 | 0.94 | 0.35 | 5.29 | 3.00 |
| 128 | Yoo et al.,2008 | 0.41 | 0.43 | -0.44 | 1.25 | 0.35 | 3.70 | 2.10 |
| 129 | Rance et al.,2023 | -0.43 | 0.34 | -1.10 | 0.24 | 0.20 | 4.96 | 2.81 |
| 138 | Pereira et al.,2023 | 1.09 | 0.43 | 0.26 | 1.93 | 0.01 | 3.76 | 2.13 |
| 144 | Scheinost et al.,2013 | 0.53 | 0.46 | -0.36 | 1.42 | 0.24 | 3.43 | 1.94 |
| 152 | Sukhodolsky et al.,2020 | 0.92 | 0.46 | 0.02 | 1.82 | 0.05 | 3.38 | 1.92 |
| 175 | Kirsch et al.,2016 | 1.14 | 0.42 | 0.31 | 1.97 | 0.01 | 3.80 | 2.15 |
| 190 | Sherwood et al.,2019 | 0.21 | 0.41 | -0.59 | 1.02 | 0.60 | 3.97 | 2.25 |
| 197 | Guan et al.,2015 | 1.83 | 0.64 | 0.57 | 3.09 | 0.00 | 2.02 | 1.14 |
| 199 | Sreedharan et al.,2020 | -0.02 | 0.71 | -1.41 | 1.36 | 0.98 | 1.71 | 0.97 |
| 217 | Direito et al.,2019 | 0.28 | 0.47 | -0.64 | 1.19 | 0.56 | 3.28 | 1.86 |
| 220 | Chung et al.,2023 | 0.17 | 0.30 | -0.42 | 0.77 | 0.56 | 5.69 | 3.22 |
| 225 | Herwig et al.,2019 | 1.12 | 0.43 | 0.28 | 1.96 | 0.01 | 3.75 | 2.13 |
| 238 | Andersson et al.,2019 | 0.73 | 0.48 | -0.21 | 1.66 | 0.13 | 3.21 | 1.82 |
| 242 | Sherwood et al.,2018 | 0.87 | 0.43 | 0.04 | 1.71 | 0.04 | 3.77 | 2.13 |
| 247 | Yao et al.,2016 | 0.76 | 0.37 | 0.03 | 1.49 | 0.04 | 4.51 | 2.56 |
| 253 | Bray S et al.,2007 | 1.13 | 0.54 | 0.07 | 2.19 | 0.04 | 2.64 | 1.50 |
| 254 | Caria et al.,2010 | 1.89 | 0.57 | 0.78 | 3.00 | 0.00 | 2.46 | 1.40 |
| 258 | McCaig et al.,2011 | 1.30 | 0.45 | 0.42 | 2.18 | 0.00 | 3.49 | 1.98 |
| | Subgroup Overall | 0.68 | 0.09 | 0.51 | 0.86 | 0.00 | | |
| Corrected | | | | | | | | |
| 3 | Papoutsi et al.,2020 | 0.82 | 0.30 | 0.22 | 1.41 | 0.01 | 5.66 | 3.21 |
| 55 | Kanel et al.,2019 | 1.22 | 0.49 | 0.26 | 2.17 | 0.01 | 3.11 | 1.76 |
| 88 | Li et al.,2018 | 0.20 | 0.39 | -0.56 | 0.96 | 0.61 | 4.22 | 2.39 |
| 91 | Scharnowski et al.,2012 | 1.48 | 0.60 | 0.30 | 2.66 | 0.01 | 2.25 | 1.27 |
| 117 | Hui et al.,2014 | 0.87 | 0.43 | 0.04 | 1.71 | 0.04 | 3.74 | 2.12 |
| 140 | Sulzer et al.,2013 | 0.11 | 0.35 | -0.58 | 0.81 | 0.76 | 4.76 | 2.69 |
| 169 | Kohl et al.,2019 | 0.13 | 0.35 | -0.56 | 0.81 | 0.72 | 4.83 | 2.74 |
| 184 | Caria et al.,2007 | 0.35 | 0.67 | -0.97 | 1.66 | 0.61 | 1.87 | 1.06 |
| 203 | Lawrence et al.,2014 | 0.66 | 0.44 | -0.21 | 1.53 | 0.14 | 3.56 | 2.01 |
| 251 | Zhang et al.2013 | 0.91 | 0.38 | 0.16 | 1.66 | 0.02 | 4.31 | 2.44 |
| 255 | deCharms et al.,2004 | 1.49 | 0.33 | 0.84 | 2.14 | 0.00 | 5.17 | 2.93 |
| 261 | Zhao X et al.,2013 | 0.71 | 0.42 | -0.12 | 1.53 | 0.09 | 3.82 | 2.16 |
| | Subgroup Overall | 0.73 | 0.15 | 0.44 | 1.02 | 0.00 | | |
| | Overall | 0.69 | 0.08 | 0.55 | 0.84 | 0.00 | | |

Model: Random-effects model
Heterogeneity: Tau-squared = 0.08, H-squared = 1.49, I-squared = 0.33
Homogeneity: Q = 68.08, df = 46, p-value = 0.02
Test of overall effect size: z = 9.20, p-value = 0.00
Test of between-subgroup homogeneity: Q = 0.07, df = 1, p-value = 0.80

### 1.2.2 target regions: cortical

n=37

**Effect Size Estimates for Subgroup Analysis**

| | Effect Size | Std. Error | Z | Sig. (2-tailed) | 95% Confidence Interval | |
|---|---|---|---|---|---|---|
| | | | | | Lower | Upper |
| DNR | .706 | .1018 | 6.931 | <.001 | .506 | .905 |
| corrected | .880 | .1629 | 5.403 | <.001 | .561 | 1.199 |
| Overall | .750 | .0869 | 8.635 | <.001 | .580 | .920 |

**Egger's Regression-Based Test[a]**

| | Parameter | Coefficient | Std. Error | t | Sig. (2-tailed) | 95% Confidence Interval | |
|---|---|---|---|---|---|---|---|
| | | | | | | Lower | Upper |
| DNR | (Intercept) | -.476 | .3947 | -1.206 | .239 | -1.287 | .335 |
| | SE[b] | 2.718 | .9069 | 2.996 | .006 | .853 | 4.582 |
| corrected | (Intercept) | .797 | .7131 | 1.118 | .301 | -.889 | 2.483 |
| | SE[b] | .205 | 1.7072 | .120 | .908 | -3.831 | 4.242 |
| Overall | (Intercept) | -.055 | .3662 | -.150 | .882 | -.798 | .688 |
| | SE[b] | 1.886 | .8431 | 2.237 | .032 | .174 | 3.597 |

a. Random-effects meta-regression

b. Standard error of effect size

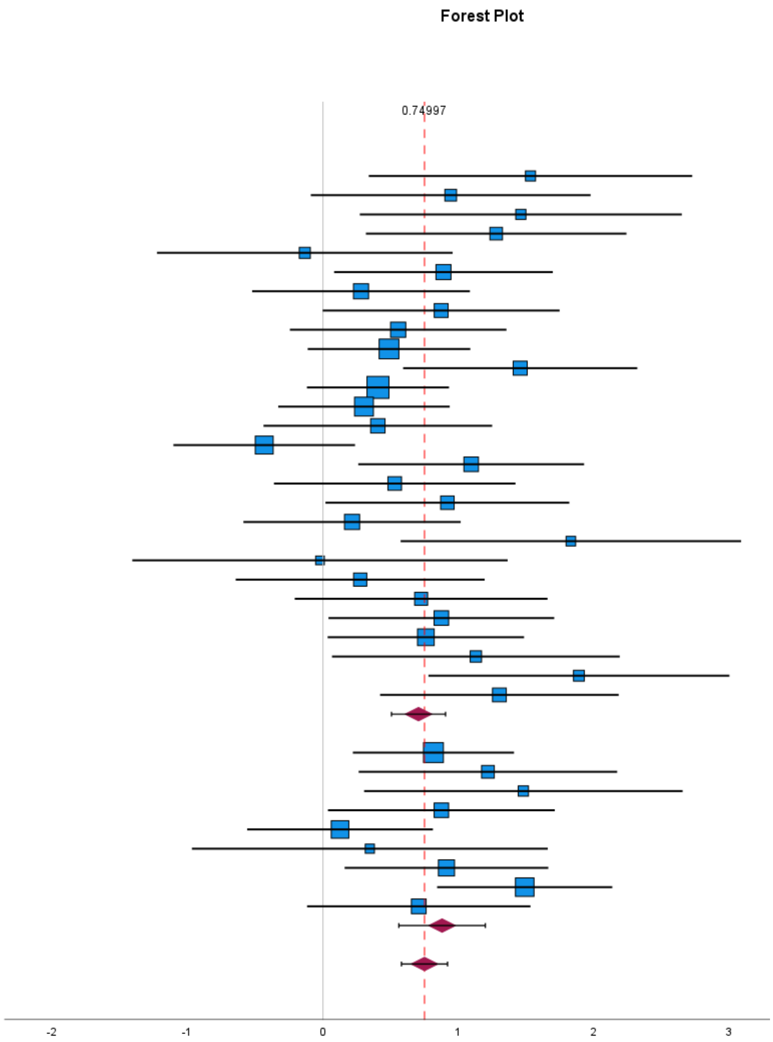


| Online | ID | Study | Effect Size | Std. Error | Lower | Upper | p-value | Weight | Weight (%) |
|---|---|---|---|---|---|---|---|---|---|
| DNR | 23 | Hohenfeld et al.,2017 | 1.53 | 0.61 | 0.34 | 2.73 | 0.01 | 2.18 | 1.64 |
| | 41 | Harmelech et al.,2015 | 0.94 | 0.53 | -0.09 | 1.98 | 0.07 | 2.74 | 2.06 |
| | 63 | Sarkheil et al.,2015 | 1.46 | 0.61 | 0.27 | 2.65 | 0.02 | 2.20 | 1.66 |
| | 65 | Sampaio-Baptista et al... | 1.28 | 0.49 | 0.32 | 2.24 | 0.01 | 3.04 | 2.30 |
| | 68 | Zilverstand et al.,2017 | -0.13 | 0.56 | -1.23 | 0.96 | 0.81 | 2.51 | 1.90 |
| | 70 | Zhang et al.,2013 | 0.89 | 0.41 | 0.08 | 1.70 | 0.03 | 3.89 | 2.93 |
| | 75 | Yoo et al.,2007 | 0.28 | 0.41 | -0.52 | 1.09 | 0.49 | 3.91 | 2.95 |
| | 93 | Yoo et al.,2006 | 0.87 | 0.45 | -0.00 | 1.75 | 0.05 | 3.49 | 2.63 |
| | 94 | Neyedli et al.,2018 | 0.56 | 0.41 | -0.24 | 1.36 | 0.17 | 3.94 | 2.97 |
| | 96 | Hartwell et al.,2016 | 0.49 | 0.31 | -0.11 | 1.09 | 0.11 | 5.51 | 4.16 |
| | 99 | Johnson et al.,2012 | 1.46 | 0.44 | 0.59 | 2.32 | 0.00 | 3.54 | 2.67 |
| | 106 | Zhang et al.,2023 | 0.41 | 0.27 | -0.12 | 0.93 | 0.13 | 6.28 | 4.74 |
| | 114 | Garrison et al.,2021 | 0.30 | 0.32 | -0.33 | 0.94 | 0.35 | 5.21 | 3.93 |
| | 128 | Yoo et al.,2008 | 0.41 | 0.43 | -0.44 | 1.25 | 0.35 | 3.66 | 2.76 |
| | 129 | Rance et al.,2023 | -0.43 | 0.34 | -1.10 | 0.24 | 0.20 | 4.89 | 3.69 |
| | 138 | Pereira et al.,2023 | 1.09 | 0.43 | 0.26 | 1.93 | 0.01 | 3.72 | 2.81 |
| | 144 | Scheinost et al.,2013 | 0.53 | 0.46 | -0.36 | 1.42 | 0.24 | 3.39 | 2.56 |
| | 152 | Sukhodolsky et al.,2020 | 0.92 | 0.46 | 0.02 | 1.82 | 0.05 | 3.35 | 2.53 |
| | 190 | Sherwood et al.,2019 | 0.21 | 0.41 | -0.59 | 1.02 | 0.60 | 3.92 | 2.96 |
| | 197 | Guan et al.,2015 | 1.83 | 0.64 | 0.57 | 3.09 | 0.00 | 2.00 | 1.51 |
| | 199 | Sreedharan et al.,2020 | -0.02 | 0.71 | -1.41 | 1.36 | 0.98 | 1.70 | 1.28 |
| | 217 | Direito et al.,2019 | 0.28 | 0.47 | -0.64 | 1.19 | 0.56 | 3.25 | 2.45 |
| | 238 | Andersson et al.,2019 | 0.73 | 0.48 | -0.21 | 1.66 | 0.13 | 3.18 | 2.40 |
| | 242 | Sherwood et al.,2018 | 0.87 | 0.43 | 0.04 | 1.71 | 0.04 | 3.73 | 2.81 |
| | 247 | Yao et al.,2016 | 0.76 | 0.37 | 0.03 | 1.49 | 0.04 | 4.46 | 3.36 |
| | 253 | Bray S et al.,2007 | 1.13 | 0.54 | 0.07 | 2.19 | 0.04 | 2.62 | 1.98 |
| | 254 | Caria et al.,2010 | 1.89 | 0.57 | 0.78 | 3.00 | 0.00 | 2.44 | 1.84 |
| | 258 | McCaig et al.,2011 | 1.30 | 0.45 | 0.42 | 2.18 | 0.00 | 3.45 | 2.61 |
| | | Subgroup Overall | 0.71 | 0.10 | 0.51 | 0.91 | 0.00 | | |
| corrected | 3 | Papoutsi et al.,2020 | 0.82 | 0.30 | 0.22 | 1.41 | 0.01 | 5.57 | 4.20 |
| | 55 | Kanel et al.,2019 | 1.22 | 0.49 | 0.26 | 2.17 | 0.01 | 3.08 | 2.32 |
| | 91 | Scharnowski et al.,2012 | 1.48 | 0.60 | 0.30 | 2.66 | 0.01 | 2.23 | 1.69 |
| | 117 | Hui et al.,2014 | 0.87 | 0.43 | 0.04 | 1.71 | 0.04 | 3.70 | 2.79 |
| | 169 | Kohl et al.,2019 | 0.13 | 0.35 | -0.56 | 0.81 | 0.72 | 4.76 | 3.59 |
| | 184 | Caria et al.,2007 | 0.35 | 0.67 | -0.97 | 1.66 | 0.61 | 1.86 | 1.40 |
| | 251 | Zhang et al.2013 | 0.91 | 0.38 | 0.16 | 1.66 | 0.02 | 4.26 | 3.21 |
| | 255 | deCharms et al.,2004 | 1.49 | 0.33 | 0.84 | 2.14 | 0.00 | 5.09 | 3.84 |
| | 261 | Zhao X et al.,2013 | 0.71 | 0.42 | -0.12 | 1.53 | 0.09 | 3.78 | 2.85 |
| | | Subgroup Overall | 0.88 | 0.16 | 0.56 | 1.20 | 0.00 | | |
| Overall | | | 0.75 | 0.09 | 0.58 | 0.92 | 0.00 | | |

Model: Random-effects model
Heterogeneity: Tau-squared = 0.09, H-squared = 1.49, I-squared = 0.33
Homogeneity: Q = 53.09, df = 36, p-value = 0.03
Test of overall effect size: z = 8.63, p-value = 0.00
Test of between-subgroup homogeneity: Q = 0.82, df = 1, p-value = 0.36

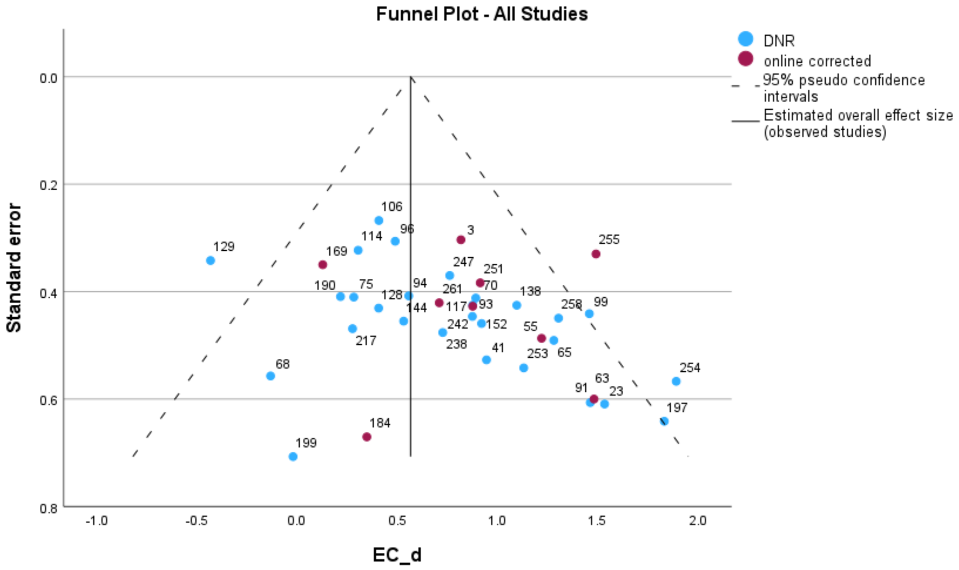

### 1.2.3 target regions: subcortical

n=10

**Effect Size Estimates for Subgroup Analysis**

| | Effect Size | Std. Error | Z | Sig. (2-tailed) | 95% Confidence Interval Lower | 95% Confidence Interval Upper |
|---|---|---|---|---|---|---|
| DNR | .610 | .1907 | 3.198 | .001 | .236 | .983 |
| corrected | .283 | .2258 | 1.255 | .209 | -.159 | .726 |
| Overall | .507 | .1437 | 3.530 | <.001 | .226 | .789 |

**Egger's Regression-Based Test[a]**

| | Parameter | Coefficient | Std. Error | t | Sig. (2-tailed) | 95% Confidence Interval Lower | 95% Confidence Interval Upper |
|---|---|---|---|---|---|---|---|
| DNR | (Intercept) | -2.173 | 1.1032 | -1.970 | .106 | -5.009 | .663 |
| | SE[b] | 7.238 | 2.8876 | 2.506 | .054 | -.185 | 14.660 |
| corrected | (Intercept) | -2.126 | 2.4968 | -.851 | .551 | -33.851 | 29.599 |
| | SE[b] | 6.186 | 6.3847 | .969 | .510 | -74.940 | 87.311 |
| Overall | (Intercept) | -2.070 | 1.0066 | -2.056 | .074 | -4.391 | .251 |
| | SE[b] | 6.691 | 2.6166 | 2.557 | .034 | .658 | 12.725 |

a. Random-effects meta-regression

b. Standard error of effect size

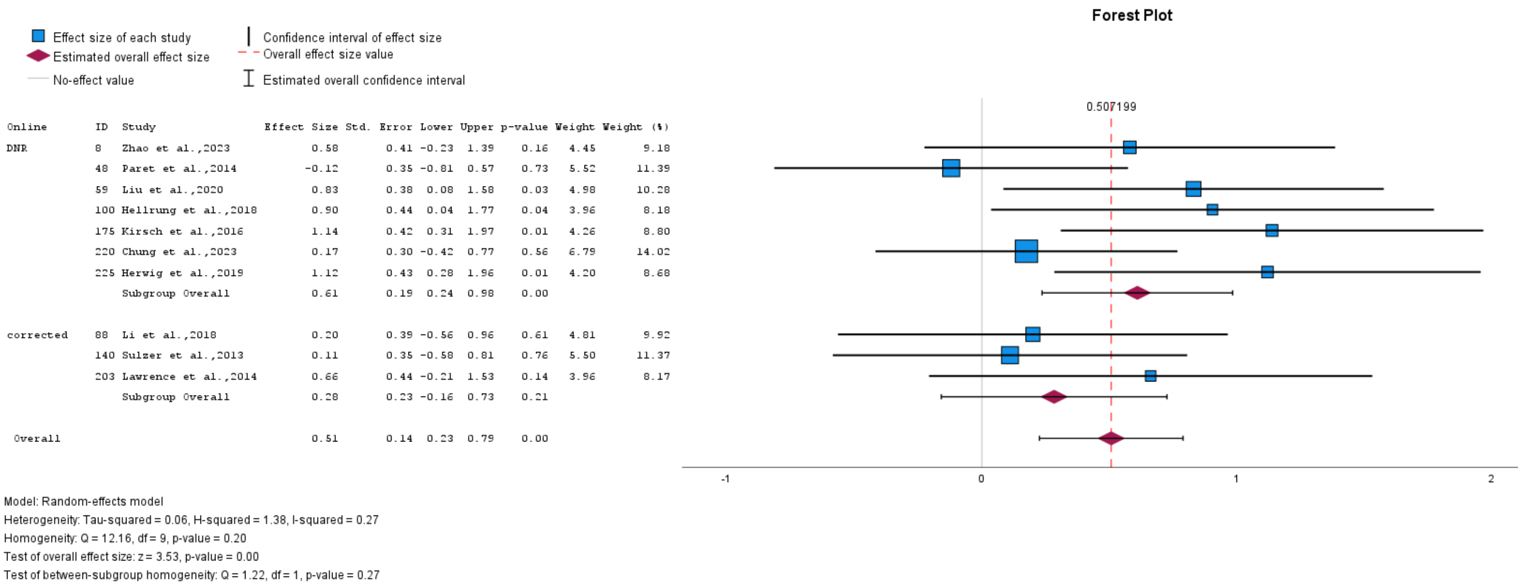


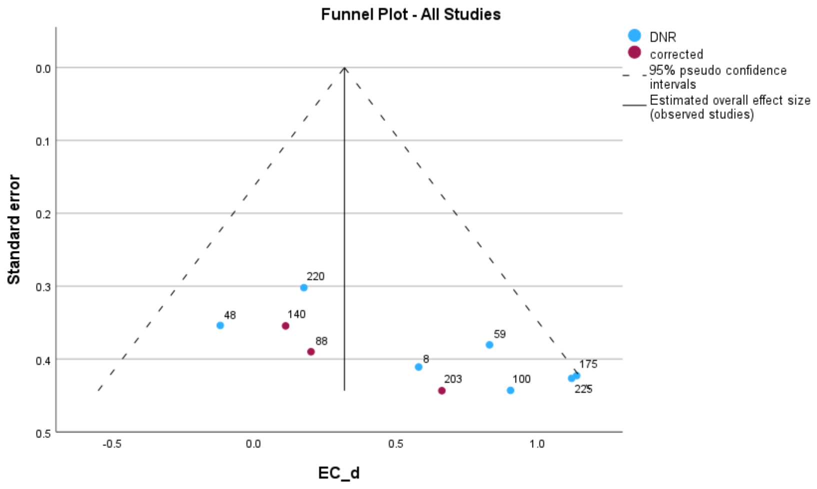


## 1.3 data type: FC

### 1.3.1 all target regions

**Effect Size Estimates for Subgroup Analysis**

| | Effect Size | Std. Error | Z | Sig. (2-tailed) | 95% Confidence Interval | |
|---|---|---|---|---|---|---|
| | | | | | Lower | Upper |
| DNR | .522 | .2834 | 1.843 | .065 | -.033 | 1.078 |
| corrected | .446 | .1422 | 3.138 | .002 | .167 | .725 |
| Overall | .461 | .1271 | 3.631 | <.001 | .212 | .711 |

**Egger's Regression-Based Test[a,b]**

| Parameter | Coefficient | Std. Error | t | Sig. (2-tailed) | 95% Confidence Interval | |
|---|---|---|---|---|---|---|
| | | | | | Lower | Upper |

| | | | | | | | |
|---|---|---|---|---|---|---|---|
| corrected | (Intercept) | .125 | .8460 | .147 | .890 | -2.224 | 2.474 |
| | SE[c] | .936 | 2.4287 | .385 | .720 | -5.807 | 7.679 |
| Overall | (Intercept) | -.020 | .7880 | -.026 | .980 | -1.948 | 1.908 |
| | SE[c] | 1.358 | 2.1919 | .619 | .558 | -4.006 | 6.721 |

a. Random-effects meta-regression
b. Regression Based Test cannot be computed for subgroup(s) Online = DNR.
c. Standard error of effect size

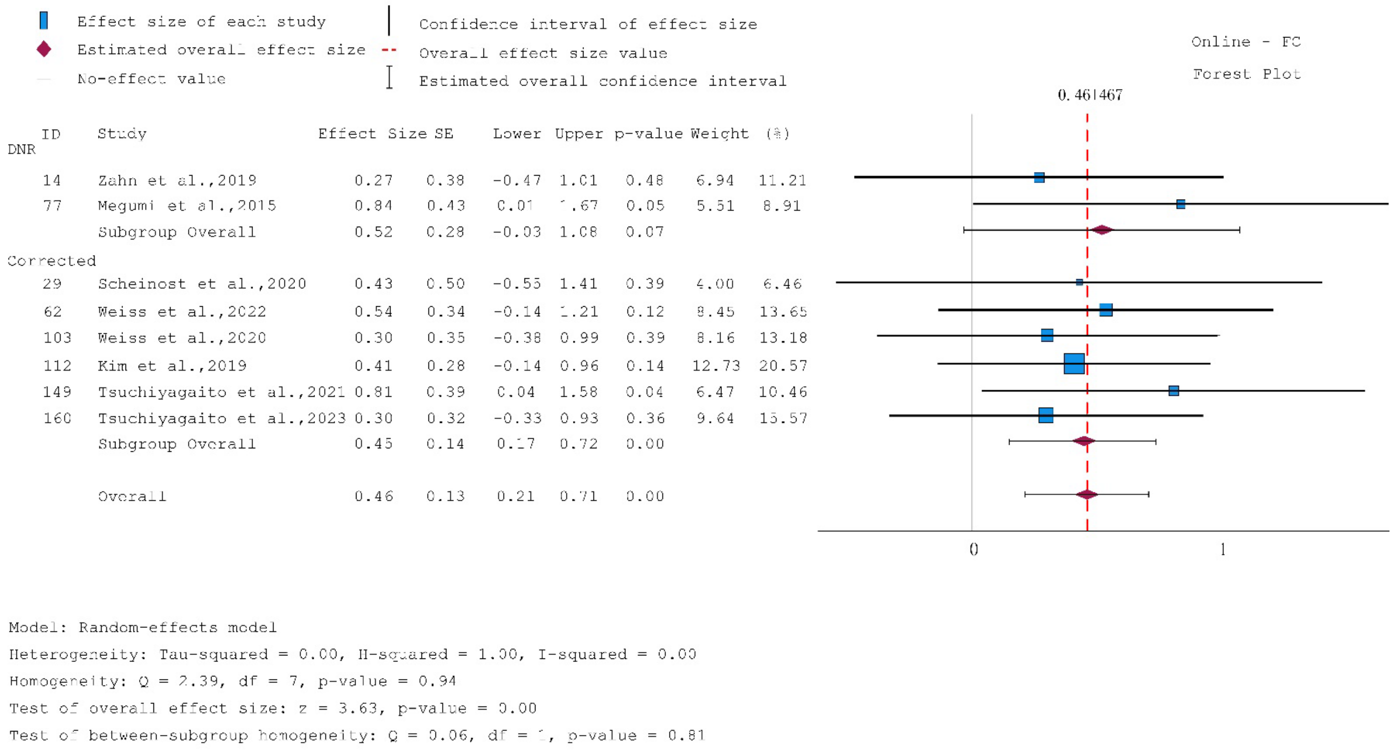

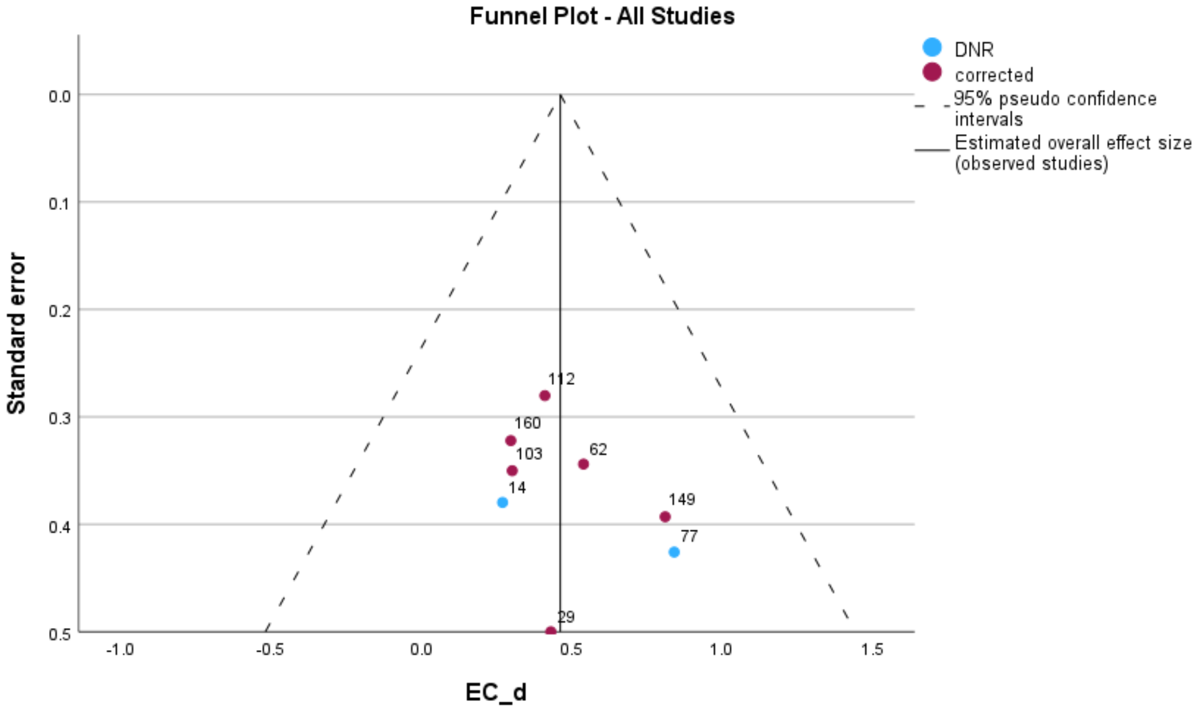
Funnel Plot - All Studies
DNR
corrected
95% pseudo confidence intervals
Estimated overall effect size (observed studies)
Standard error
0.0
0.1
0.2
0.3
0.4
0.5
-1.0
-0.5
0.0
0.5
1.0
1.5
EC_d
112
160
103
62
14
149
77
29

## 2. Effect of different online correction methods

### 2.1 data type: BOLD

#### 2.1.1 all target regions

n=49

**Effect Size Estimates for Subgroup Analysis**

| | Effect Size | Std. Error | Z | Sig. (2-tailed) | 95% Confidence Interval Lower | 95% Confidence Interval Upper |
|---|---|---|---|---|---|---|
| DNR | .682 | .0886 | 7.698 | <.001 | .508 | .855 |
| Physio-signal based | .816 | .3036 | 2.688 | .007 | .221 | 1.411 |
| Image-signal based | .746 | .1742 | 4.281 | <.001 | .404 | 1.087 |
| Instruction | .546 | .2891 | 1.888 | .059 | -.021 | 1.112 |
| Overall | .683 | .0731 | 9.342 | <.001 | .539 | .826 |

**Egger's Regression-Based Test[a,b]**

| | Parameter | Coefficient | Std. Error | t | Sig. (2-tailed) | 95% Confidence Interval Lower | 95% Confidence Interval Upper |
|---|---|---|---|---|---|---|---|
| DNR | (Intercept) | -.605 | .3476 | -1.741 | .091 | -1.312 | .102 |
| | SE[c] | 3.043 | .8248 | 3.689 | <.001 | 1.364 | 4.721 |
| Image signal-based | (Intercept) | 1.113 | .9645 | 1.154 | .286 | -1.168 | 3.394 |
| | SE[c] | -.892 | 2.2940 | -.389 | .709 | -6.316 | 4.533 |
| Instruction | (Intercept) | -1.610 | 1.0338 | -1.557 | .260 | -6.058 | 2.838 |
| | SE[c] | 5.384 | 2.6200 | 2.055 | .176 | -5.889 | 16.657 |
| Overall | (Intercept) | -.320 | .3222 | -.993 | .326 | -.968 | .328 |
| | SE[c] | 2.420 | .7689 | 3.147 | .003 | .873 | 3.967 |

a. Random-effects meta-regression

b. Regression Based Test cannot be computed for subgroup(s) Online = Physio signal-based.

c. Standard error of effect size

Effect size of each study | Confidence interval of effect size
Estimated overall effect size | Overall effect size value
No-effect value | Estimated overall confidence interval

Online Methods - BOLD

Forest Plot

| ID | Study | Effect Size | SE | Lower | Upper | p-value | Weight | (%) |
|---|---|---|---|---|---|---|---|---|
| DNR | | | | | | | | |
| 8 | Zhao et al.,2023 | 0.58 | 0.41 | -0.23 | 1.39 | 0.16 | 3.98 | 2.12 |
| 23 | Hohenfeld et al.,2017 | 1.53 | 0.61 | 0.34 | 2.73 | 0.01 | 2.20 | 1.18 |
| 41 | Karmelech et al.,2015 | 0.94 | 0.53 | -0.09 | 1.98 | 0.07 | 2.78 | 1.48 |
| 48 | Paret et al.,2014 | -0.12 | 0.35 | -0.81 | 0.57 | 0.73 | 4.81 | 2.57 |
| 59 | Liu et al.,2020 | 0.83 | 0.38 | 0.08 | 1.58 | 0.03 | 4.40 | 2.35 |
| 63 | Sarkheil et al.,2015 | 1.46 | 0.61 | 0.27 | 2.65 | 0.02 | 2.22 | 1.19 |
| 65 | Sampaio-Baptista et al... | 1.28 | 0.49 | 0.32 | 2.24 | 0.01 | 3.09 | 1.65 |
| 68 | Zilverstand et al.,2017 | -0.13 | 0.56 | -1.23 | 0.96 | 0.81 | 2.55 | 1.36 |
| 70 | Zhang et al.,2013 | 0.89 | 0.41 | 0.08 | 1.70 | 0.03 | 3.96 | 2.12 |
| 75 | Yoo et al.,2007 | 0.28 | 0.41 | -0.52 | 1.09 | 0.49 | 3.99 | 2.13 |
| 93 | Yoo et al.,2006 | 0.87 | 0.45 | -0.00 | 1.75 | 0.05 | 3.55 | 1.90 |
| 94 | Nevedli et al.,2018 | 0.56 | 0.41 | -0.24 | 1.36 | 0.17 | 4.02 | 2.15 |
| 96 | Hartwell et al.,2016 | 0.49 | 0.31 | -0.11 | 1.09 | 0.11 | 5.67 | 3.03 |
| 99 | Johnson et al.,2012 | 1.46 | 0.44 | 0.59 | 2.32 | 0.00 | 3.61 | 1.93 |
| 100 | Hellrung et al.,2018 | 0.90 | 0.44 | 0.04 | 1.77 | 0.04 | 3.59 | 1.92 |
| 106 | Zhang et al.,2023 | 0.41 | 0.27 | -0.12 | 0.93 | 0.13 | 6.49 | 3.46 |
| 114 | Garrison et al.,2021 | 0.30 | 0.32 | -0.33 | 0.94 | 0.35 | 5.35 | 2.86 |
| 128 | Yoo et al.,2008 | 0.41 | 0.43 | -0.44 | 1.25 | 0.35 | 3.73 | 1.99 |
| 129 | Rance et al.,2023 | -0.43 | 0.34 | -1.10 | 0.24 | 0.20 | 5.01 | 2.68 |
| 138 | Pereira et al.,2023 | 1.09 | 0.43 | 0.26 | 1.93 | 0.01 | 3.79 | 2.03 |
| 144 | Scheinost et al.,2013 | 0.53 | 0.46 | -0.36 | 1.42 | 0.24 | 3.45 | 1.84 |
| 152 | Sukhodolsky et al.,2020 | 0.92 | 0.46 | 0.02 | 1.82 | 0.05 | 3.41 | 1.82 |
| 175 | Kirsch et al.,2016 | 1.14 | 0.42 | 0.31 | 1.97 | 0.01 | 3.83 | 2.04 |
| 190 | Sherwood et al.,2019 | 0.21 | 0.41 | -0.59 | 1.02 | 0.60 | 4.00 | 2.14 |
| 197 | Guan et al.,2015 | 1.83 | 0.64 | 0.57 | 3.09 | 0.00 | 2.02 | 1.08 |
| 199 | Sreedharan et al.,2020 | -0.02 | 0.71 | -1.41 | 1.36 | 0.98 | 1.72 | 0.92 |
| 217 | Direito et al.,2019 | 0.28 | 0.47 | -0.64 | 1.19 | 0.56 | 3.31 | 1.77 |
| 220 | Chung et al.,2023 | 0.17 | 0.30 | -0.42 | 0.77 | 0.56 | 5.75 | 3.07 |
| 225 | Herwig et al.,2019 | 1.12 | 0.43 | 0.28 | 1.96 | 0.01 | 3.78 | 2.02 |
| 238 | Andersson et al.,2019 | 0.73 | 0.48 | -0.21 | 1.66 | 0.13 | 3.23 | 1.73 |
| 242 | Sherwood et al.,2018 | 0.87 | 0.43 | 0.04 | 1.71 | 0.04 | 3.80 | 2.03 |
| 247 | Yao et al.,2016 | 0.76 | 0.37 | 0.03 | 1.49 | 0.04 | 4.56 | 2.43 |
| 253 | Bray et al.,2007 | 1.13 | 0.54 | 0.07 | 2.19 | 0.04 | 2.66 | 1.42 |
| 254 | Caria et al.,2010 | 1.89 | 0.57 | 0.78 | 3.00 | 0.00 | 2.48 | 1.32 |
| 258 | McCaig et al.,2011 | 1.30 | 0.45 | 0.42 | 2.18 | 0.00 | 3.51 | 1.88 |
| | Subgroup Overall | 0.68 | 0.09 | 0.51 | 0.86 | 0.00 | | |
| Physio signal-based | | | | | | | | |
| 3 | Papoutsi et al.,2020 | 0.82 | 0.30 | 0.22 | 1.41 | 0.01 | 5.73 | 3.06 |
| | Subgroup Overall | 0.82 | 0.30 | 0.22 | 1.41 | 0.01 | | |
| Image signal-based | | | | | | | | |
| 55 | Kanel et al.,2019 | 1.22 | 0.49 | 0.26 | 2.17 | 0.01 | 3.13 | 1.67 |
| 88 | Li et al.,2018 | 0.20 | 0.39 | -0.56 | 0.96 | 0.61 | 4.26 | 2.28 |
| 117 | Hui et al.,2014 | 0.87 | 0.43 | 0.04 | 1.71 | 0.04 | 3.77 | 2.01 |
| 169 | Kohl et al.,2019 | 0.13 | 0.35 | -0.56 | 0.81 | 0.72 | 4.88 | 2.61 |
| 184 | Caria et al.,2007 | 0.35 | 0.67 | -0.97 | 1.66 | 0.61 | 1.88 | 1.00 |
| 203 | Lawrence et al.,2014 | 0.66 | 0.44 | -0.21 | 1.53 | 0.14 | 3.58 | 1.91 |
| 251 | Zhang et al.2013 | 0.91 | 0.38 | 0.16 | 1.66 | 0.02 | 4.35 | 2.32 |
| 255 | deCharms et al.,2004 | 1.49 | 0.33 | 0.84 | 2.14 | 0.00 | 5.22 | 2.79 |
| 261 | Zhao et al.,2013 | 0.71 | 0.42 | -0.12 | 1.53 | 0.09 | 3.85 | 2.06 |
| | Subgroup Overall | 0.75 | 0.17 | 0.40 | 1.09 | 0.00 | | |
| Instruction | | | | | | | | |
| 91 | Scharnowski et al.,2012 | 1.48 | 0.60 | 0.30 | 2.66 | 0.01 | 2.26 | 1.21 |
| 140 | Sulzer et al.,2013 | 0.11 | 0.35 | -0.58 | 0.81 | 0.76 | 4.80 | 2.57 |
| 169 | Kohl et al.,2019 | 0.13 | 0.35 | -0.56 | 0.81 | 0.72 | 4.88 | 2.61 |
| 251 | Zhang et al.2013 | 0.91 | 0.38 | 0.16 | 1.66 | 0.02 | 4.35 | 2.32 |
| | Subgroup Overall | 0.55 | 0.29 | -0.02 | 1.11 | 0.06 | | |
| Overall | | 0.68 | 0.07 | 0.54 | 0.83 | 0.00 | | |

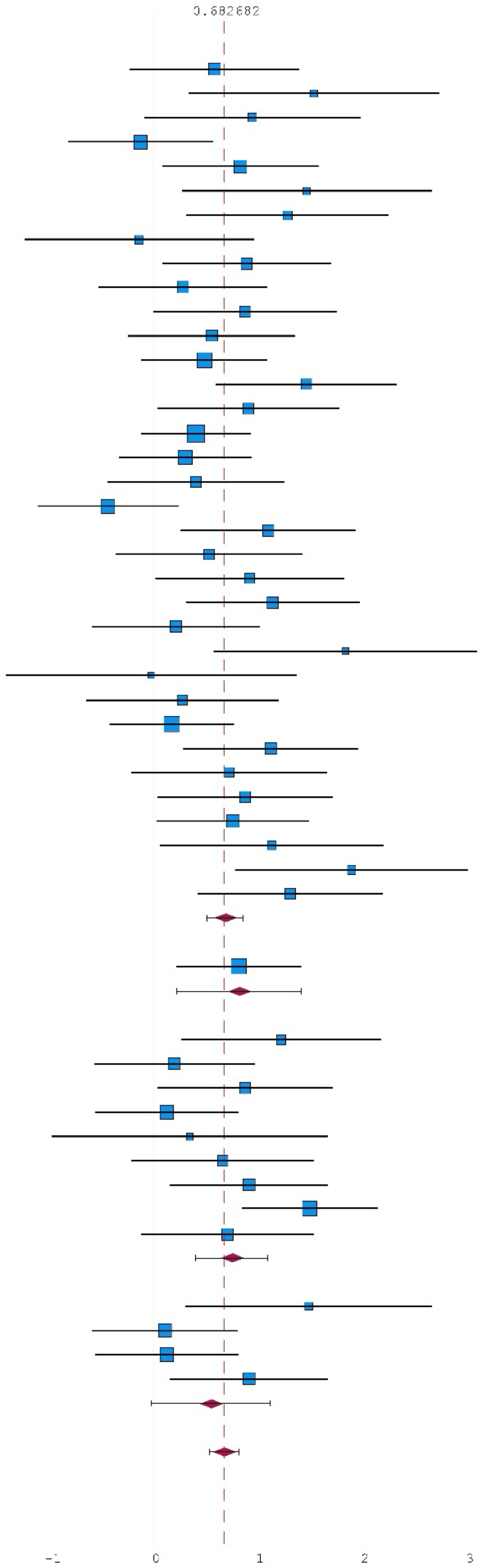


Model: Random-effects model

Heterogeneity: Tau-squared = 0.08, H-squared = 1.49, I-squared = 0.33

Homogeneity: Q = 70.83, df = 48, p-value = 0.02

Test of overall effect size: z = 9.34, p-value = 0.00

Test of between-subgroup homogeneity: Q = 0.53, df = 3, p-value = 0.91

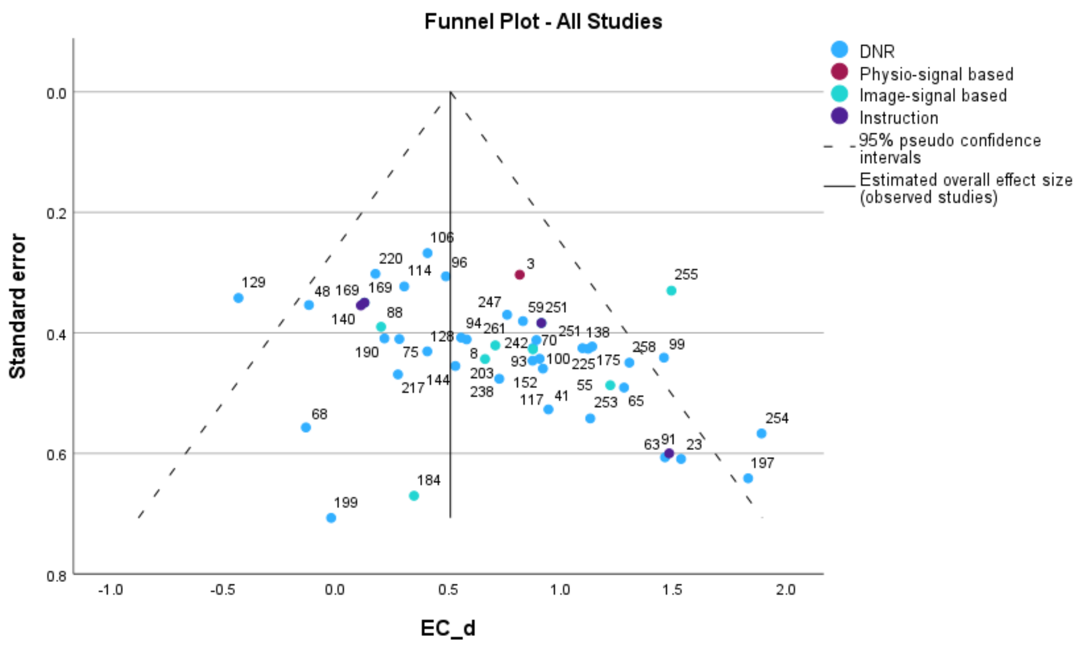


### 2.1.2 target regions: cortical

n=39

**Effect Size Estimates for Subgroup Analysis**

| | Effect Size | Std. Error | Z | Sig. (2-tailed) | 95% Confidence Interval Lower | 95% Confidence Interval Upper |
|---|---|---|---|---|---|---|
| DNR | .706 | .1018 | 6.931 | <.001 | .506 | .905 |
| Physio signal-based | .816 | .3036 | 2.688 | .007 | .221 | 1.411 |
| Image signal-based | .842 | .2023 | 4.162 | <.001 | .445 | 1.238 |
| Instruction | .738 | .3780 | 1.952 | .051 | -.003 | 1.479 |
| Overall | .734 | .0838 | 8.753 | <.001 | .569 | .898 |

**Egger's Regression-Based Test[a,b]**

| | Parameter | Coefficient | Std. Error | t | Sig. (2-tailed) | 95% Confidence Interval Lower | 95% Confidence Interval Upper |
|---|---|---|---|---|---|---|---|
| DNR | (Intercept) | -.476 | .3947 | -1.206 | .239 | -1.287 | .335 |
| | SE[c] | 2.718 | .9069 | 2.996 | .006 | .853 | 4.582 |
| Image signal-based | (Intercept) | 1.306 | 1.0074 | 1.297 | .251 | -1.283 | 3.896 |
| | SE[c] | -1.126 | 2.3804 | -.473 | .656 | -7.245 | 4.993 |

| | | | | | | | |
|---|---|---|---|---|---|---|---|
| Instruction | (Intercept) | -1.250 | 1.2882 | -.970 | .510 | -17.617 | 15.118 |
| | SE[c] | 4.708 | 3.0606 | 1.538 | .367 | -34.180 | 43.597 |
| Overall | (Intercept) | -.106 | .3556 | -.298 | .768 | -.826 | .615 |
| | SE[c] | 1.985 | .8274 | 2.400 | .022 | .309 | 3.662 |

a. Random-effects meta-regression

b. Regression Based Test cannot be computed for subgroup(s) Online = Physio signal-based.

c. Standard error of effect size

Forest Plot

Effect size of each study
Estimated overall effect size
No-effect value
Confidence interval of effect size
Overall effect size value
Estimated overall confidence interval

| Online | ID | Study | Effect Size | Std. Error | Lower | Upper | p-value | Weight | Weight (%) |
|---|---|---|---|---|---|---|---|---|---|
| DNR | 23 | Hohenfeld et al.,2017 | 1.53 | 0.61 | 0.34 | 2.73 | 0.01 | 2.19 | 1.54 |
| | 41 | Harmelech et al.,2015 | 0.94 | 0.53 | -0.09 | 1.98 | 0.07 | 2.75 | 1.93 |
| | 63 | Sarkheil et al.,2015 | 1.46 | 0.61 | 0.27 | 2.65 | 0.02 | 2.20 | 1.55 |
| | 65 | Sampaio-Baptista et al... | 1.28 | 0.49 | 0.32 | 2.24 | 0.01 | 3.06 | 2.15 |
| | 68 | Zilverstand et al.,2017 | -0.13 | 0.56 | -1.23 | 0.96 | 0.81 | 2.52 | 1.77 |
| | 70 | Zhang et al.,2013 | 0.89 | 0.41 | 0.08 | 1.70 | 0.03 | 3.91 | 2.75 |
| | 75 | Yoo et al.,2007 | 0.28 | 0.41 | -0.52 | 1.09 | 0.49 | 3.93 | 2.76 |
| | 93 | Yoo et al.,2006 | 0.87 | 0.45 | -0.00 | 1.75 | 0.05 | 3.51 | 2.46 |
| | 94 | Neyedli et al.,2018 | 0.56 | 0.41 | -0.24 | 1.36 | 0.17 | 3.96 | 2.78 |
| | 96 | Hartwell et al.,2016 | 0.49 | 0.31 | -0.11 | 1.09 | 0.11 | 5.56 | 3.90 |
| | 99 | Johnson et al.,2012 | 1.46 | 0.44 | 0.59 | 2.32 | 0.00 | 3.56 | 2.50 |
| | 106 | Zhang et al.,2023 | 0.41 | 0.27 | -0.12 | 0.93 | 0.13 | 6.34 | 4.45 |
| | 114 | Garrison et al.,2021 | 0.30 | 0.32 | -0.33 | 0.94 | 0.35 | 5.25 | 3.69 |
| | 128 | Yoo et al.,2008 | 0.41 | 0.43 | -0.44 | 1.25 | 0.35 | 3.68 | 2.59 |
| | 129 | Rance et al.,2023 | -0.43 | 0.34 | -1.10 | 0.24 | 0.20 | 4.92 | 3.46 |
| | 138 | Pereira et al.,2023 | 1.09 | 0.43 | 0.26 | 1.93 | 0.01 | 3.74 | 2.63 |
| | 144 | Scheinost et al.,2013 | 0.53 | 0.46 | -0.36 | 1.42 | 0.24 | 3.41 | 2.40 |
| | 152 | Sukhodolsky et al.,2020 | 0.92 | 0.46 | 0.02 | 1.82 | 0.05 | 3.37 | 2.36 |
| | 190 | Sherwood et al.,2019 | 0.21 | 0.41 | -0.59 | 1.02 | 0.60 | 3.94 | 2.77 |
| | 197 | Guan et al.,2015 | 1.83 | 0.64 | 0.57 | 3.09 | 0.00 | 2.01 | 1.41 |
| | 199 | Sreedharan et al.,2020 | -0.02 | 0.71 | -1.41 | 1.36 | 0.98 | 1.71 | 1.20 |
| | 217 | Direito et al.,2019 | 0.28 | 0.47 | -0.64 | 1.19 | 0.56 | 3.27 | 2.29 |
| | 238 | Andersson et al.,2019 | 0.73 | 0.48 | -0.21 | 1.66 | 0.13 | 3.20 | 2.24 |
| | 242 | Sherwood et al.,2018 | 0.87 | 0.43 | 0.04 | 1.71 | 0.04 | 3.75 | 2.63 |
| | 247 | Yao et al.,2016 | 0.76 | 0.37 | 0.03 | 1.49 | 0.04 | 4.48 | 3.15 |
| | 253 | Bray S et al.,2007 | 1.13 | 0.54 | 0.07 | 2.19 | 0.04 | 2.63 | 1.85 |
| | 254 | Caria et al.,2010 | 1.89 | 0.57 | 0.78 | 3.00 | 0.00 | 2.45 | 1.72 |
| | 258 | McCaig et al.,2011 | 1.30 | 0.45 | 0.42 | 2.18 | 0.00 | 3.47 | 2.44 |
| | | Subgroup Overall | 0.71 | 0.10 | 0.51 | 0.91 | 0.00 | | |
| Physio signal-based | 3 | Papoutsi et al.,2020 | 0.82 | 0.30 | 0.22 | 1.41 | 0.01 | 5.61 | 3.94 |
| | | Subgroup Overall | 0.82 | 0.30 | 0.22 | 1.41 | 0.01 | | |
| Image signal-based | 55 | Kanel et al.,2019 | 1.22 | 0.49 | 0.26 | 2.17 | 0.01 | 3.09 | 2.17 |
| | 117 | Hui et al.,2014 | 0.87 | 0.43 | 0.04 | 1.71 | 0.04 | 3.72 | 2.61 |
| | 169 | Kohl et al.,2019 | 0.13 | 0.35 | -0.56 | 0.81 | 0.72 | 4.79 | 3.37 |
| | 184 | Caria et al.,2007 | 0.35 | 0.67 | -0.97 | 1.66 | 0.61 | 1.87 | 1.31 |
| | 251 | Zhang et al.2013 | 0.91 | 0.38 | 0.16 | 1.66 | 0.02 | 4.29 | 3.01 |
| | 255 | deCharms et al.,2004 | 1.49 | 0.33 | 0.84 | 2.14 | 0.00 | 5.13 | 3.60 |
| | 261 | Zhao X et al.,2013 | 0.71 | 0.42 | -0.12 | 1.53 | 0.09 | 3.80 | 2.67 |
| | | Subgroup Overall | 0.84 | 0.20 | 0.45 | 1.24 | 0.00 | | |
| Instruction | 91 | Scharnowski et al.,2012 | 1.48 | 0.60 | 0.30 | 2.66 | 0.01 | 2.24 | 1.57 |
| | 169 | Kohl et al.,2019 | 0.13 | 0.35 | -0.56 | 0.81 | 0.72 | 4.79 | 3.37 |
| | 251 | Zhang et al.2013 | 0.91 | 0.38 | 0.16 | 1.66 | 0.02 | 4.29 | 3.01 |
| | | Subgroup Overall | 0.74 | 0.38 | -0.00 | 1.48 | 0.05 | | |
| Overall | | | 0.73 | 0.08 | 0.57 | 0.90 | 0.00 | | |

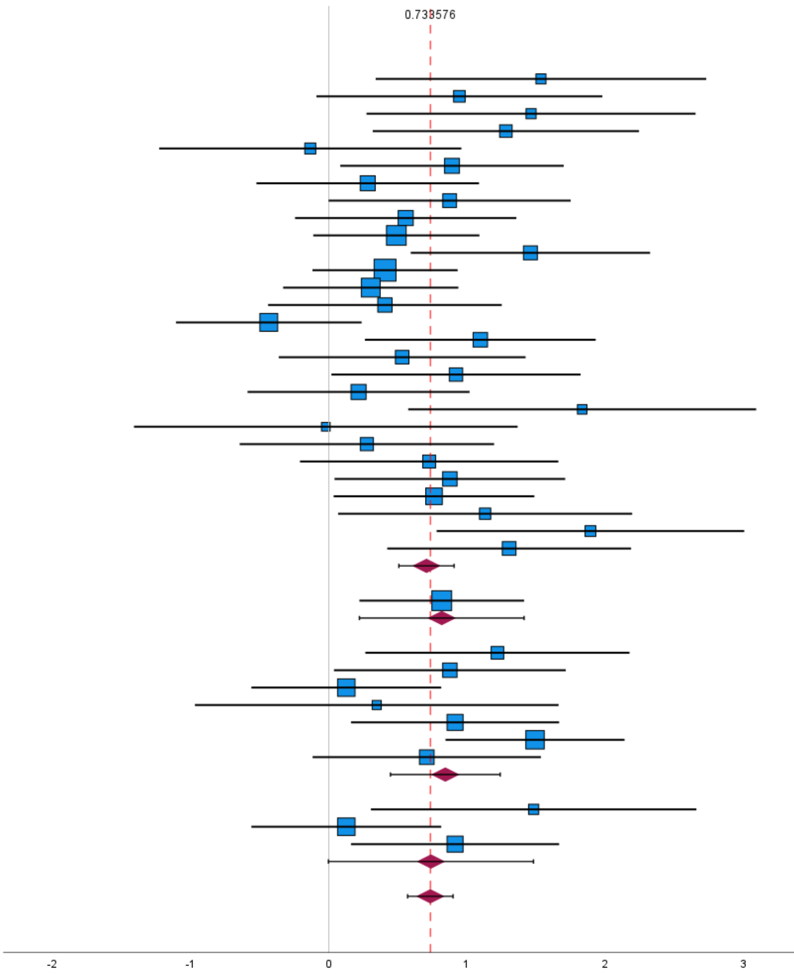


Model: Random-effects model
Heterogeneity: Tau-squared = 0.09, H-squared = 1.49, I-squared = 0.33
Homogeneity: Q = 56.17, df = 38, p-value = 0.03
Test of overall effect size: z = 8.75, p-value = 0.00
Test of between-subgroup homogeneity: Q = 0.43, df = 3, p-value = 0.93

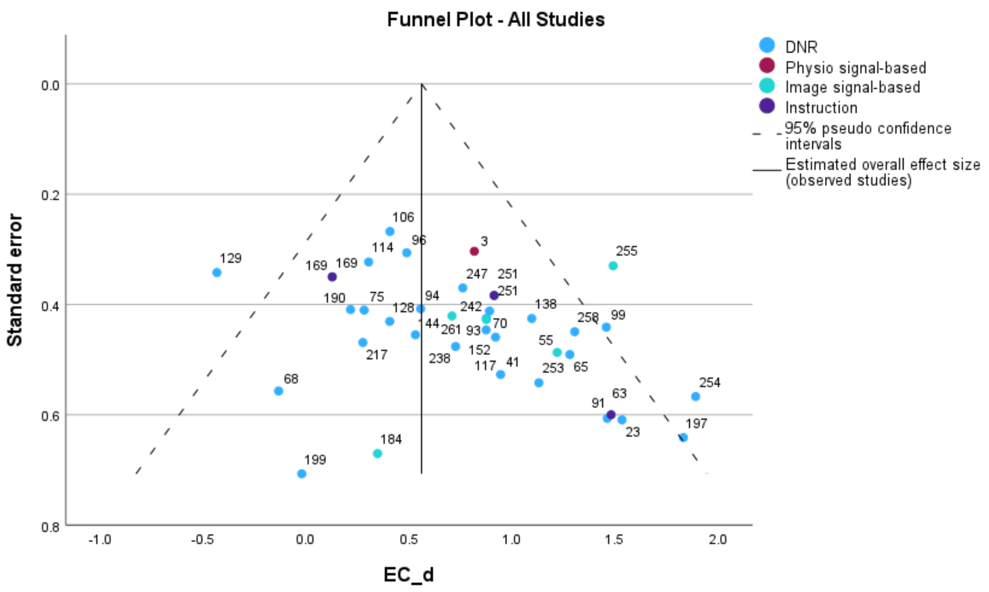


### 2.1.3 target regions: subcortical

n=10

**Effect Size Estimates for Subgroup Analysis**

| | Effect Size | Std. Error | Z | Sig. (2-tailed) | 95% Confidence Interval Lower | 95% Confidence Interval Upper |
|---|---|---|---|---|---|---|
| DNR | .610 | .1907 | 3.198 | .001 | .236 | .983 |
| Image signal-based | .401 | .2928 | 1.371 | .170 | -.173 | .975 |
| Instruction | .110 | .3545 | .311 | .756 | -.585 | .805 |
| Overall | .507 | .1437 | 3.530 | <.001 | .226 | .789 |

**Egger's Regression-Based Test[a,b]**

| | Parameter | Coefficient | Std. Error | t | Sig. (2-tailed) | 95% Confidence Interval Lower | 95% Confidence Interval Upper |
|---|---|---|---|---|---|---|---|
| DNR | (Intercept) | -2.173 | 1.1032 | -1.970 | .106 | -5.009 | .663 |
| | SE[c] | 7.238 | 2.8876 | 2.506 | .054 | -.185 | 14.660 |
| Overall | (Intercept) | -2.070 | 1.0066 | -2.056 | .074 | -4.391 | .251 |
| | SE[c] | 6.691 | 2.6166 | 2.557 | .034 | .658 | 12.725 |

a. Random-effects meta-regression

b. Regression Based Test cannot be computed for subgroup(s) Online = Image signal-based, Instruction.

## c. Standard error of effect size

Forest Plot

Effect size of each study
Estimated overall effect size
No-effect value
Confidence interval of effect size
Overall effect size value
Estimated overall confidence interval

| Online | ID | Study | Effect Size | Std. Error | Lower | Upper | p-value | Weight | Weight (%) |
|---|---|---|---|---|---|---|---|---|---|
| DNR | 8 | Zhao et al.,2023 | 0.58 | 0.41 | -0.23 | 1.39 | 0.16 | 4.45 | 9.18 |
| | 48 | Paret et al.,2014 | -0.12 | 0.35 | -0.81 | 0.57 | 0.73 | 5.52 | 11.39 |
| | 59 | Liu et al.,2020 | 0.83 | 0.38 | 0.08 | 1.58 | 0.03 | 4.98 | 10.28 |
| | 100 | Hellrung et al.,2018 | 0.90 | 0.44 | 0.04 | 1.77 | 0.04 | 3.96 | 8.18 |
| | 175 | Kirsch et al.,2016 | 1.14 | 0.42 | 0.31 | 1.97 | 0.01 | 4.26 | 8.80 |
| | 220 | Chung et al.,2023 | 0.17 | 0.30 | -0.42 | 0.77 | 0.56 | 6.79 | 14.02 |
| | 225 | Herwig et al.,2019 | 1.12 | 0.43 | 0.28 | 1.96 | 0.01 | 4.20 | 8.68 |
| | | Subgroup Overall | 0.61 | 0.19 | 0.24 | 0.98 | 0.00 | | |
| Image signal-based | 88 | Li et al.,2018 | 0.20 | 0.39 | -0.56 | 0.96 | 0.61 | 4.81 | 9.92 |
| | 203 | Lawrence et al.,2014 | 0.66 | 0.44 | -0.21 | 1.53 | 0.14 | 3.96 | 8.17 |
| | | Subgroup Overall | 0.40 | 0.29 | -0.17 | 0.98 | 0.17 | | |
| Instruction | 140 | Sulzer et al.,2013 | 0.11 | 0.35 | -0.58 | 0.81 | 0.76 | 5.50 | 11.37 |
| | | Subgroup Overall | 0.11 | 0.35 | -0.58 | 0.81 | 0.76 | | |
| Overall | | | 0.51 | 0.14 | 0.23 | 0.79 | 0.00 | | |

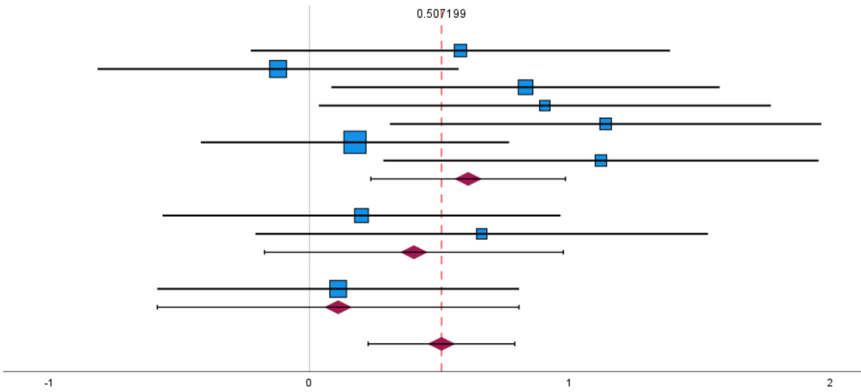


Model: Random-effects model
Heterogeneity: Tau-squared = 0.06, H-squared = 1.38, I-squared = 0.27
Homogeneity: Q = 12.16, df = 9, p-value = 0.20
Test of overall effect size: z = 3.53, p-value = 0.00
Test of between-subgroup homogeneity: Q = 1.62, df = 2, p-value = 0.44

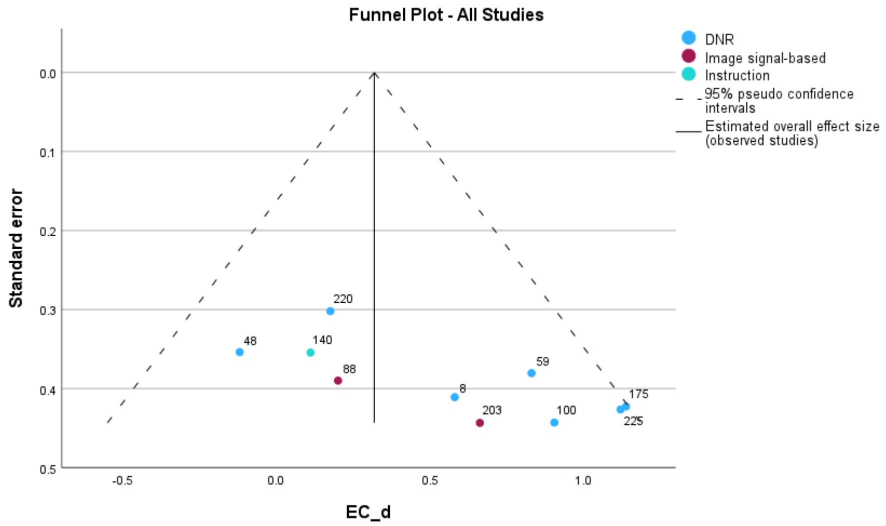

## Included studies

| NO. | ID | author | publication year | title | reference |
|---|---|---|---|---|---|
| 1 | 1 | Ruiz, Sergio; Lee, Sangkyun; Soekadar, Surjo R.; Caria, Andrea; Veit, Ralf; Kircher, Tilo; Birbaumer, Niels; Sitaram, Ranganatha | 2013 | Acquired self-control of insula cortex modulates emotion recognition and brain network connectivity in schizophrenia | (Ruiz et al., 2013) |
| 2 | 3 | Papoutsi, Marina; Magerkurth, Joerg; Josephs, Oliver; Pepes, Sophia E.; Ibitoye, Temi; Reilmann, Ralf; Hunt, Nigel; Payne, Edwin; Weiskopf, Nikolaus; Langbehn, Douglas; Rees, Geraint; Tabrizi, Sarah J. | 2020 | Activity or connectivity? A randomized controlled feasibility study evaluating neurofeedback training in Huntington's disease | (Papoutsi et al., 2020) |
| 3 | 4 | Paret, Christian; Kluetsch, Rosemarie; Zaehringer, Jenny; Ruf, Matthias; Demirakca, Traute; Bohus, Martin; Ende, Gabriele; Schmahl, Christian | 2016 | Alterations of amygdala-prefrontal connectivity with real-time fMRI neurofeedback in BPD patients | (Paret et al., 2016) |
| 4 | 5 | Gao, Hui; Zhang, Huan; Wang, Linyuan; Zhang, Chi; Feng, Zhiyuan; Li, Zhonglin; Tong, Li; Yan, Bin; Hu, Guoen | 2023 | Altered amygdala functional connectivity after real-time functional MRI emotion self-regulation training | (Gao et al., 2023) |
| 5 | 6 | Li, Zhonglin; Tong, Li; Guan, Min; He, Wenjie; Wang, Linyuan; Bu, Haibin; Shi, Dapeng; Yan, Bin | 2016 | Altered Resting-State Amygdala Functional Connectivity after Real-Time fMRI Emotion Self-Regulation Training | (Z. Li, Tong, Guan, et al., 2016) |
| 6 | 7 | Quevedo, Karina; Teoh, Jia Yuan; Engstrom, Maggie; Wedan, Riley; Santana-Gonzalez, Carmen; Zewde, Betanya; Porter, | 2020 | Amygdala Circuitry During Neurofeedback Training and Symptoms' Change in Adolescents With Varying Depression | (Quevedo et al., 2020) |

| | | | | | |
|---|---|---|---|---|---|
| | | David; Kadosh, Kathrin Cohen | | | |
| 7 | 8 | Zhao, Zhiying; Duek, Or; Seidemann, Rebecca; Gordon, Charles; Walsh, Christopher; Romaker, Emma; Koller, William N.; Horvath, Mark; Awasthi, Jitendra; Wang, Yao; O'Brien, Erin; Fichtenholtz, Harlan; Hampson, Michelle; Harpaz-Rotem, Ilan | 2023 | Amygdala downregulation training using fMRI neurofeedback in post-traumatic stress disorder: a randomized, double-blind trial | (Z. Zhao et al., 2023) |
| 8 | 9 | Wang, Yutong; Yao, Li; Zhao, Xiaojie | 2020 | Amygdala network in response to facial expression following neurofeedback training of emotion | (Y. Wang et al., 2020) |
| 9 | 10 | Marxen, Michael; Jacob, Mark J.; Mueller, Dirk K.; Posse, Stefan; Ackley, Elena; Hellrung, Lydia; Riedel, Philipp; Bender, Stephan; Epple, Robert; Smolka, Michael N. | 2016 | Amygdala Regulation Following fMRI-Neurofeedback without Instructed Strategies | (Marxen et al., 2016) |
| 10 | 12 | Takamura, Masahiro; Okamoto, Yasumasa; Shibasaki, Chiyo; Yoshino, Atsuo; Okada, Go; Ichikawa, Naho; Yamawaki, Shigeto | 2020 | Antidepressive effect of left dorsolateral prefrontal cortex neurofeedback in patients with major depressive disorder: A preliminary report | (Takamura et al., 2020) |
| 11 | 13 | Hampson, Michelle; Scheinost, Dustin; Qiu, Maolin; Bhawnani, Jitendra; Lacadie, Cheryl M.; Leckman, James F.; Constable, R. Todd; Papademetris, Xenophon | 2011 | Biofeedback of real-time functional magnetic resonance imaging data from the supplementary motor area reduces functional connectivity to subcortical regions | (Hampson et al., 2011) |
| 12 | 14 | Zahn, Roland; Weingartner, Julie H.; Basilio, Rodrigo; | 2019 | Blame-rebalance fMRI neurofeedback in | (Zahn et al., 2019) |

| | | | | | |
|---|---|---|---|---|---|
| | | Bado, Patricia; Mattos, Paulo; Sato, Joao R.; de Oliveira-Souza, Ricardo; Fontenelle, Leo F.; Young, Allan H.; Moll, Jorge | | major depressive disorder: A randomised proof-of-concept trial | |
| 13 | 15 | Mehler, David M. A.; Williams, Angharad N.; Krause, Florian; Luehrs, Michael; Wise, Richard G.; Turner, Duncan L.; Linden, David E. J.; Whittaker, Joseph R. | 2019 | The BOLD response in primary motor cortex and supplementary motor area during kinesthetic motor imagery based graded fMRI neurofeedback | (Mehler et al., 2019) |
| 14 | 17 | Dewiputri, Wan Ilma; Schweizer, Renate; Auer, Tibor | 2021 | Brain Networks Underlying Strategy Execution and Feedback Processing in an Efficient Functional Magnetic Resonance Imaging Neurofeedback Training Performed in a Parallel or a Serial Paradigm | (Dewiputri et al., 2021) |
| 15 | 20 | Debettencourt, Megan T.; Cohen, Jonathan D.; Lee, Ray F.; Norman, Kenneth A.; Turk-Browne, Nicholas B. | 2015 | Closed-loop training of attention with real-time brain imaging | (Debettencourt et al., 2015) |
| 16 | 21 | Mennen, Anne C.; Turk-Browne, Nicholas B.; Wallace, Grant; Seok, Darsol; Jaganjac, Adna; Stock, Janet; DeBettencourt, Megan T.; Cohen, Jonathan D.; Norman, Kenneth A.; Sheline, Yvette I. | 2021 | Cloud-Based Functional Magnetic Resonance Imaging Neurofeedback to Reduce the Negative Attentional Bias in Depression: A Proof-of-Concept Study | (Mennen et al., 2021) |
| 17 | 22 | Cordes, Julia S.; Mathiak, Krystyna A.; Dyck, Miriam; Alawi, Eliza M.; Gaber, Tilman J.; Zepf, Florian D.; Klasen, Martin; Zvyagintsev, Mikhail; Gur, Ruben C.; Mathiak, Klaus | 2015 | Cognitive and neural strategies during control of the anterior cingulate cortex by fMRI neurofeedback in patients with schizophrenia | (Cordes et al., 2015) |

| | | | | | |
|---|---|---|---|---|---|
| 18 | 23 | Hohenfeld, Christian; Nellessen, Nils; Dogan, Imis; Kuhn, Hanna; Mueller, Christine; Papa, Federica; Ketteler, Simon; Goebel, Rainer; Heinecke, Armin; Shah, N. Jon; Schulz, Joerg B.; Reske, Martina; Reetz, Kathrin | 2017 | Cognitive Improvement and Brain Changes after Real-Time Functional MRI Neurofeedback Training in Healthy Elderly and Prodromal Alzheimer's Disease | (Hohenfeld et al., 2017) |
| 19 | 24 | MacInnes, J. J.; Dickerson, K. C.; Chen, N. K.; Adcock, R. A. | 2016 | Cognitive Neurostimulation: Learning to Volitionally Sustain Ventral Tegmental Area Activation | (MacInnes et al., 2016) |
| 20 | 26 | Emmert, Kirsten; Breimhorst, Markus; Bauermann, Thomas; Birklein, Frank; Van de Ville, Dimitri; Haller, Sven | 2014 | Comparison of anterior cingulate vs. insular cortex as targets for real-time fMRI regulation during pain stimulation | (Emmert et al., 2014) |
| 21 | 27 | Yamashita, Ayumu; Hayasaka, Shunsuke; Kawato, Mitsuo; Imamizu, Hiroshi | 2017 | Connectivity Neurofeedback Training Can Differentially Change Functional Connectivity and Cognitive Performance | (Yamashita et al., 2017) |
| 22 | 28 | Koush, Yury; Rosa, Maria Joao; Robineau, Fabien; Heinen, Klaartje; Rieger, Sebastian W.; Weiskopf, Nikolaus; Vuilleumier, Patrik; Van De Ville, Dimitri; Scharnowski, Frank | 2013 | Connectivity-based neurofeedback: Dynamic causal modeling for real-time fMRI | (Koush et al., 2013) |
| 23 | 29 | Scheinost, Dustin; Hsu, Tiffany W.; Avery, Emily W.; Hampson, Michelle; Constable, R. Todd; Chun, Marvin M.; Rosenberg, Monica D. | 2020 | Connectome-based neurofeedback: A pilot study to improve sustained attention | (Scheinost et al., 2020) |
| 24 | 30 | Emmert, Kirsten; Kopel, Rotem; Koush, Yury; Maire, Raphael; Senn, Pascal; Van De | 2017 | Continuous vs. intermittent neurofeedback to regulate auditory cortex activity of | (Emmert et al., 2017) |

| | | | | | |
|---|---|---|---|---|---|
| | | Ville, Dimitri; Haller, Sven | | tinnitus patients using real-time fMRI - A pilot study | |
| 25 | 31 | Sousa, Teresa; Direito, Bruno; Lima, Joao; Ferreira, Carlos; Nunes, Urbano; Castelo-Branco, Miguel | 2016 | Control of Brain Activity in hMT+/V5 at Three Response Levels Using fMRI-Based Neurofeedback/BCI | (Sousa et al., 2016) |
| 26 | 32 | Greer, Stephanie M.; Trujillo, Andrew J.; Glover, Gary H.; Knutson, Brian | 2014 | Control of nucleus accumbens activity with neurofeedback | (Greer et al., 2014) |
| 27 | 36 | Ramot, Michal; Grossman, Shany; Friedman, Doron; Malach, Rafael | 2016 | Covert neurofeedback without awareness shapes cortical network spontaneous connectivity | (Ramot et al., 2016) |
| 28 | 37 | Harmelech, Tal; Preminger, Son; Wertman, Eliahu; Malach, Rafael | 2013 | The Day-After Effect: Long Term, Hebbian-Like Restructuring of Resting-State fMRI Patterns Induced by a Single Epoch of Cortical Activation | (Harmelech et al., 2013) |
| 29 | 38 | Cortese, Aurelio; Amano, Kaoru; Koizumi, Ai; Lau, Hakwan; Kawato, Mitsuo | 2017 | Decoded fMRI neurofeedback can induce bidirectional confidence changes within single participants | (Cortese et al., 2017) |
| 30 | 39 | Taylor, Jessica Elizabeth; Yamada, Takashi; Kawashima, Takahiko; Kobayashi, Yuko; Yoshihara, Yujiro; Miyata, Jun; Murai, Toshiya; Kawato, Mitsuo; Motegi, Tomokazu | 2022 | Depressive symptoms reduce when dorsolateral prefrontal cortex-precuneus connectivity normalizes after functional connectivity neurofeedback | (Taylor et al., 2022) |
| 31 | 40 | Shibata, Kazuhisa; Watanabe, Takeo; Kawato, Mitsuo; Sasaki, Yuka | 2016 | Differential Activation Patterns in the Same Brain Region Led to Opposite Emotional States | (Shibata et al., 2016) |
| 32 | 41 | Harmelech, Tal; Friedman, Doron; Malach, Rafael | 2015 | Differential Magnetic Resonance | (Harmelech et al., 2015) |

| | | | | | |
|---|---|---|---|---|---|
| | | | | Neurofeedback Modulations across Extrinsic (Visual) and Intrinsic (Default-Mode) Nodes of the Human Cortex | |
| 33 | 42 | Nicholson, Andrew A.; Rabellino, Daniela; Densmore, Maria; Frewen, Paul A.; Steryl, David; Scharnowski, Frank; Theberge, Jean; Neufeld, Richard W. J.; Schmahl, Christian; Jetly, Rakesh; Lanius, Ruth A. | 2022 | Differential mechanisms of posterior cingulate cortex downregulation and symptom decreases in posttraumatic stress disorder and healthy individuals using real-time fMRI neurofeedback | (Nicholson et al., 2022) |
| 34 | 43 | Oblak, Ethan; Lewis-Peacock, Jarrod; Sulzer, James | 2021 | Differential neural plasticity of individual fingers revealed by fMRI neurofeedback | (Oblak et al., 2021) |
| 35 | 44 | Ramot, Michel; Kimmich, Sara; Gonzalez-Castillo, Javier; Roopchansingh, Vinai; Popal, Haroon; White, Emily; Gotts, Stephen J.; Martin, Alex | 2017 | Direct modulation of aberrant brain network connectivity through real-time NeuroFeedback | (Ramot et al., 2017) |
| 36 | 45 | Direito, Bruno; Ramos, Manuel; Pereira, Joao; Sayal, Alexandre; Sousa, Teresa; Castelo-Branco, Miguel | 2021 | Directly Exploring the Neural Correlates of Feedback-Related Reward Saliency and Valence During Real-Time fMRI-Based Neurofeedback | (Direito, Ramos, et al., 2021) |
| 37 | 47 | Lam, Sheut-Ling; Criaud, Marion; Lukito, Steve; Westwood, Samuel J.; Agbedjro, Deborah; Kowalczyk, Olivia S.; Curran, Sarah; Barret, Nadia; Abbott, Chris; Liang, Holan; Simonoff, Emily; Barker, Gareth | 2022 | Double-Blind, Sham-Controlled Randomized Trial Testing the Efficacy of fMRI Neurofeedback on Clinical and Cognitive Measures in Children With ADHD | (Lam et al., 2022) |

| | | | | | |
|---|---|---|---|---|---|
| | | J.; Giampietro, Vincent; Rubia, Katya | | | |
| 38 | 48 | Paret, Christian; Kluetsch, Rosemarie; Ruf, Matthias; Demirakca, Traute; Hoesterey, Steffen; Ende, Gabriele; Schmahl, Christian | 2014 | Down-regulation of amygdala activation with real-time fMRI neurofeedback in a healthy female sample | (Paret et al., 2014) |
| 39 | 51 | Kober, Silvia Erika; Groessinger, Doris; Wood, Guilherme | 2019 | Effects of Motor Imagery and Visual Neurofeedback on Activation in the Swallowing Network: A Real-Time fMRI Study | (Kober et al., 2019) |
| 40 | 52 | Yang, Huixiang; Hu, Zhengfei; Imai, Fumihito; Yang, Yuxiang; Ogawa, Kenji | 2021 | Effects of neurofeedback on the activities of motor-related areas by using motor execution and imagery | (Yang et al., 2021) |
| 41 | 53 | Hamilton, J. Paul; Glover, Gary H.; Bagarinao, Epifanio; Chang, Catie; Mackey, Sean; Sacchet, Matthew D.; Gotlib, Ian H. | 2016 | Effects of salience-network-node neurofeedback training on affective biases in major depressive disorder | (Hamilton et al., 2016) |
| 42 | 54 | Zhu, Yashuo; Gao, Hui; Tong, Li; Li, ZhongLin; Wang, Linyuan; Zhang, Chi; Yang, Qiang; Yan, Bin | 2019 | Emotion Regulation of Hippocampus Using Real-Time fMRI Neurofeedback in Healthy Human | (Zhu et al., 2019) |
| 43 | 55 | Kanel, Dana; Al-Wasity, Salim; Stefanov, Kristian; Pollick, Frank E. | 2019 | Empathy to emotional voices and the use of real-time fMRI to enhance activation of the anterior insula | (Kanel et al., 2019) |
| 44 | 56 | Sherwood, Matthew S.; Kane, Jessica H.; Weisend, Michael P.; Parker, Jason G. | 2016 | Enhanced control of dorsolateral prefrontal cortex neurophysiology with real-time functional magnetic resonance imaging (rt-fMRI) neurofeedback training and | (Sherwood et al., 2016) |

| | | | | | |
|---|---|---|---|---|---|
| | | | | working memory practice | |
| 45 | 58 | Marins, Theo F.; Rodrigues, Erika C.; Engel, Annerose; Hoefle, Sebastian; Basilio, Rodrigo; Lent, Roberto; Moll, Jorge; Tovar-Moll, Fernanda | 2015 | Enhancing Motor Network Activity Using Real-Time Functional MRI Neurofeedback of Left Premotor Cortex | (T. F. Marins et al., 2015) |
| 46 | 59 | Liu, Ning; Yao, Li; Zhao, Xiaojie | 2020 | Evaluating the amygdala network induced by neurofeedback training for emotion regulation using hierarchical clustering | (Liu et al., 2020) |
| 47 | 60 | Bottinger, Boris W.; Aggensteiner, Pascal-M.; Hohmann, Sarah; Heintz, Stefan; Ruf, Matthias; Glennon, Jeffrey; Holz, Nathalie E.; Banaschewski, Tobias; Brandeis, Daniel; Baumeister, Sarah | 2023 | Exploring real-time functional magnetic resonance imaging neurofeedback in adolescents with disruptive behavior disorder and callous unemotional traits | (Bottinger et al., 2023) |
| 48 | 61 | Koizumi, Ai; Amano, Kaoru; Cortese, Aurelio; Shibata, Kazuhisa; Yoshida, Wako; Seymour, Ben; Kawato, Mitsuo; Lau, Hakwan | 2017 | Fear reduction without fear through reinforcement of neural activity that bypasses conscious exposure | (Koizumi et al., 2017) |
| 49 | 62 | Weiss, Franziska; Zhang, Jingying; Aslan, Acelya; Kirsch, Peter; Gerchen, Martin Fungisai | 2022 | Feasibility of training the dorsolateral prefrontal-striatal network by real-time fMRI neurofeedback | (Weiss et al., 2022) |
| 50 | 63 | Sarkheil, Pegah; Zilverstand, Anna; Kilian-Hutten, Niclas; Schneider, Frank; Goebel, Rainer; Mathiak, Klaus | 2015 | fMRI feedback enhances emotion regulation as evidenced by a reduced amygdala response | (Sarkheil et al., 2015) |
| 51 | 64 | Zilverstand, Anna; Sorger, Bettina; Sarkheil, Pegah; Goebel, Rainer | 2015 | fMRI neurofeedback facilitates anxiety regulation in females with spider phobia | (Zilverstand et al., 2015) |

| | | | | | |
|---|---|---|---|---|---|
| 52 | 65 | Sampaio-Baptista, Cassandra; Neyedli, Heather F.; Sanders, Zeena-Britt; Diosi, Kata; Havard, David; Huang, YunYing; Andersson, Jesper L. R.; Luhr, Michael; Goebel, Rainer; Johansen-Berg, Heidi | 2021 | fMRI neurofeedback in the motor system elicits bidirectional changes in activity and in white matter structure in the adult human brain | (Sampaio-Baptista et al., 2021) |
| 53 | 67 | Habes, I.; Rushton, S.; Johnston, S. J.; Sokunbi, M. O.; Barawi, K.; Brosnan, M.; Daly, T.; Ihssen, N.; Linden, D. E. J. | 2016 | fMRI neurofeedback of higher visual areas and perceptual biases | (Habes et al., 2016) |
| 54 | 68 | Zilverstand, Anna; Sorger, Bettina; Slaats-Willemse, Dorine; Kan, Cornelis C.; Goebel, Rainer; Buitelaar, Jan K. | 2017 | fMRI Neurofeedback Training for Increasing Anterior Cingulate Cortex Activation in Adult Attention Deficit Hyperactivity Disorder. An Exploratory Randomized, Single-Blinded Study | (Zilverstand et al., 2017) |
| 55 | 69 | Keller, Micha; Zweerings, Jana; Klasen, Martin; Zvyagintsev, Mikhail; Iglesias, Jorge; Quinones, Raul Mendoza; Mathiak, Klaus | 2021 | fMRI Neurofeedback-Enhanced Cognitive Reappraisal Training in Depression: A Double-Blind Comparison of Left and Right vlPFC Regulation | (Keller et al., 2021) |
| 56 | 70 | Zhang, Gaoyan; Zhang, Hang; Li, Xiaoli; Zhao, Xiaojie; Yao, Li; Long, Zhiying | 2013 | Functional Alteration of the DMN by Learned Regulation of the PCC Using Real-Time fMRI | (G. Zhang, Zhang, et al., 2013) |
| 57 | 71 | Karch, Susanne; Krause, Daniela; Lehnert, Kevin; Konrad, Julia; Haller, Dinah; Rauchmann, Boris-Stephan; Maywald, Maximilian; Engelbregt, Hessel; | 2022 | Functional and clinical outcomes of FMRI-based neurofeedback training in patients with alcohol dependence: a pilot study | (Karch et al., 2022) |

| | | | | | |
|---|---|---|---|---|---|
| | | Adorjan, Kristina; Koller, Gabriele; Reidler, Paul; Karali, Temmuz; Tschentscher, Nadja; Ertl-Wagner, Birgit; Pogarell, Oliver; Paolini, Marco; Keeser, Daniel | | | |
| 58 | 72 | Xie, Fufang; Xu, Lele; Long, Zhiying; Yao, Li; Wu, Xia | 2015 | Functional connectivity alteration after real-time fMRI motor imagery training through self-regulation of activities of the right premotor cortex | (Xie et al., 2015) |
| 59 | 74 | Subramanian, Leena; Morris, Monica Busse; Brosnan, Meadhbh; Turner, Duncan L.; Morris, Huw R.; Linden, David E. J. | 2016 | Functional Magnetic Resonance Imaging Neurofeedback-guided Motor Imagery Training and Motor Training for Parkinson's Disease: Randomized Trial | (Subramanian et al., 2016) |
| 60 | 75 | Yoo, Seung-Schik; Lee, Jong-Hwan; O'Leary, Heather; Lee, Vivian; Choo, Seh-Eun; Jolesz, Ferenc A. | 2007 | Functional magnetic resonance imaging-mediated learning of increased activity in auditory areas | (S.-S. Yoo et al., 2007) |
| 61 | 76 | Yoo, S. S.; Jolesz, F. A. | 2002 | Functional MRI for neurofeedback: feasibility study on a hand motor task | (S. S. Yoo & Jolesz, 2002) |
| 62 | 77 | Megumi, Fukuda; Yamashita, Ayumu; Kawato, Mitsuo; Imamizu, Hiroshi | 2015 | Functional MRI neurofeedback training on connectivity between two regions induces long-lasting changes in intrinsic functional network | (Megumi et al., 2015) |
| 63 | 78 | Mehler, David M. A.; Williams, Angharad N.; Whittaker, Joseph R.; Krause, Florian; Luhrs, Michael; Kunas, Stefanie; | 2020 | Graded fMRI Neurofeedback Training of Motor Imagery in Middle Cerebral Artery Stroke Patients: A | (Mehler et al., 2020) |

| | | | | | |
|---|---|---|---|---|---|
| | | Wise, Richard G.; Shetty, Hamsaraj G. M.; Turner, Duncan L.; Linden, David E. J. | | Preregistered Proof-of-Concept Study | |
| 64 | 81 | Sepulveda, Pradyumna; Sitaram, Ranganatha; Rana, Mohit; Montalba, Cristian; Tejos, Cristian; Ruiz, Sergio | 2016 | How Feedback, Motor Imagery, and Reward Influence Brain Self-Regulation Using Real-Time fMRI | (Sepulveda et al., 2016) |
| 65 | 83 | Zweerings, Jana; Pflieger, Eliza M.; Mathiak, Krystyna A.; Zvyagintsev, Mikhail; Kacela, Anastasia; Flatten, Guido; Mathiak, Klaus | 2018 | Impaired Voluntary Control in PTSD: Probing Self-Regulation of the ACC With Real-Time fMRI | (Zweerings et al., 2018) |
| 66 | 84 | Zaehringer, Jenny; Ende, Gabriele; Santangelo, Philip; Kleindienst, Nikolaus; Ruf, Matthias; Bertsch, Katja; Bohus, Martin; Schmahl, Christian; Paret, Christian | 2019 | Improved emotion regulation after neurofeedback: A single-arm trial in patients with borderline personality disorder | (Zaehringer et al., 2019) |
| 67 | 85 | Li, Zhonglin; Liu, Jiao; Chen, Bairu; Wu, Xiaoling; Zou, Zhi; Gao, Hui; Wang, Caiyun; Zhou, Jing; Qi, Fei; Zhang, Miao; He, Junya; Qi, Xin; Yan, Fengshan; Dou, Shewei; Tong, Li; Zhang, Hongju; Han, Xingmin; Li, Yongli | 2022 | Improved Regional Homogeneity in Chronic Insomnia Disorder After Amygdala-Based Real-Time fMRI Neurofeedback Training | (Z. Li et al., 2022) |
| 68 | 86 | Bagarinao, Epifanio; Yoshida, Akihiro; Ueno, Mika; Terabe, Kazunori; Kato, Shohei; Isoda, Haruo; Nakai, Toshiharu | 2018 | Improved Volitional Recall of Motor-Imagery-Related Brain Activation Patterns Using Real-Time Functional MRI-Based Neurofeedback | (Bagarinao et al., 2018) |
| 69 | 87 | Blefari, Maria L.; Sulzer, James; Hepp-Reymond, Marie-Claude; Kollias, Spyros; Gassert, Roger | 2015 | Improvement in precision grip force control with self-modulation of primary motor cortex during motor imagery | (Blefari et al., 2015) |

| | | | | | |
|---|---|---|---|---|---|
| 70 | 88 | Li, Zhi; Zhang, Chen-yuan; Huang, Jia; Wang, Yi; Yan, Chao; Li, Ke; Zeng, Ya-wei; Jin, Zhen; Cheung, Eric F. C.; Su, Li; Chan, Raymond C. K. | 2018 | Improving Motivation Through Real-Time fMRI-Based Self-Regulation of the Nucleus Accumbens | (Z. Li et al., 2018) |
| 71 | 89 | Liew, Sook-Lei; Rana, Mohit; Cornelsen, Sonja; de Barros Filho, Marcos Fortunato; Birbaumer, Niels; Sitaram, Ranganatha; Cohen, Leonardo G.; Soekadar, Surjo R. | 2016 | Improving Motor Corticothalamic Communication After Stroke Using Real-Time fMRI Connectivity-Based Neurofeedback | (Liew et al., 2016) |
| 72 | 90 | Bagarinao, Epifanio; Yoshida, Akihiro; Terabe, Kazunori; Kato, Shohei; Nakai, Toshiharu | 2020 | Improving Real-Time Brain State Classification of Motor Imagery Tasks During Neurofeedback Training | (Bagarinao et al., 2020) |
| 73 | 91 | Scharnowski, Frank; Hutton, Chloe; Josephs, Oliver; Weiskopf, Nikolaus; Rees, Geraint | 2012 | Improving Visual Perception through Neurofeedback | (Scharnowski et al., 2012) |
| 74 | 92 | Kim, Dong-Youl; Yoo, Seung-Schik; Tegethoff, Marion; Meinlschmidt, Gunther; Lee, Jong-Hwan | 2015 | The Inclusion of Functional Connectivity Information into fMRI-based Neurofeedback Improves Its Efficacy in the Reduction of Cigarette Cravings | (D. Y. Kim et al., 2015) |
| 75 | 93 | Yoo, Seung-Schik; O'Leary, Heather M.; Fairneny, Ty; Chen, Nan-Kuei; Panych, Lawrence P.; Park, HyunWook; Jolesz, Ferenc A. | 2006 | Increasing cortical activity in auditory areas through neurofeedback functional magnetic resonance imaging | (S.-S. Yoo et al., 2006) |
| 76 | 94 | Neyedli, Heather F.; Sampaio-Baptista, Cassandra; Kirkman, Matthew A.; Havard, David; Luhrs, Michael; Ramsden, Katie; Flitney, David D.; Clare, Stuart; Goebel, Rainer; Johansen-Berg, Heidi | 2018 | Increasing Lateralized Motor Activity in Younger and Older Adults using Real-time fMRI during Executed Movements | (Neyedli et al., 2018) |

| | | | | | |
|---|---|---|---|---|---|
| 77 | 95 | Maywald, M.; Paolini, M.; Rauchmann, B. S.; Gerz, C.; Heppe, J. L.; Wolf, A.; Lerchenberger, L.; Tominschek, I.; Stöcklein, S.; Reidler, P.; Tschentscher, N.; Ertl-Wagner, B.; Pogarell, O.; Keeser, D.; Karch, S. | 2022 | Individual- and Connectivity-Based Real-Time fMRI Neurofeedback to Modulate Emotion-Related Brain Responses in Patients with Depression: A Pilot Study | (Maywald et al., 2022) |
| 78 | 96 | Hartwell, Karen J.; Hanlon, Colleen A.; Li, Xingbao; Borckardt, Jeffrey J.; Canterberry, Melanie; Prisciandaro, James J.; Moran-Santa Maria, Megan M.; LeMatty, Todd; George, Mark S.; Brady, Kathleen T. | 2016 | Individualized real-time fMRI neurofeedback to attenuate craving in nicotine-dependent smokers | (Hartwell et al., 2016) |
| 79 | 97 | Collin, Silvy H. P.; van den Broek, Philip L. C.; van Mourik, Tim; Desain, Peter; Doeller, Christian F. | 2022 | Inducing a mental context for associative memory formation with real-time fMRI neurofeedback | (Collin et al., 2022) |
| 80 | 98 | Tinaz, Sule; Para, Kiran; Vives-Rodriguez, Ana; Martinez-Kaigi, Valeria; Nalamada, Keerthana; Sezgin, Mine; Scheinost, Dustin; Hampson, Michelle; Louis, Elan D.; Constable, R. Todd | 2018 | Insula as the Interface Between Body Awareness and Movement: A Neurofeedback-Guided Kinesthetic Motor Imagery Study in Parkinson's Disease | (Tinaz et al., 2018) |
| 81 | 99 | Johnson, Kevin A.; Hartwell, Karen; LeMatty, Todd; Borckardt, Jeffrey; Morgan, Paul S.; Govindarajan, Koushik; Brady, Kathleen; George, Mark S. | 2012 | Intermittent "Real-time" fMRI Feedback Is Superior to Continuous Presentation for a Motor Imagery Task: A Pilot Study | (Johnson et al., 2012) |
| 82 | 100 | Hellrung, Lydia; Dietrich, Anja; Hollmann, Maurice; Pleger, Burkhard; Kalberlah, Christian; | 2018 | Intermittent compared to continuous real-time fMRI neurofeedback | (Hellrung et al., 2018) |

| | | | | | |
|---|---|---|---|---|---|
| | | Roggenhofer, Elisabeth; Villringer, Arno; Horstmann, Annette | | boosts control over amygdala activation | |
| 83 | 102 | Chiew, Mark; LaConte, Stephen M.; Graham, Simon J. | 2012 | Investigation of fMRI neurofeedback of differential primary motor cortex activity using kinesthetic motor imagery | (Chiew et al., 2012) |
| 84 | 103 | Weiss, Franziska; Zamoscik, Vera; Schmidt, Stephanie N. L.; Halli, Patrick; Kirsch, Peter; Gerchen, Martin Fungisai | 2020 | Just a very expensive breathing training? Risk of respiratory artefacts in functional connectivity-based real-time fMRI neurofeedback | Just a very expensive breathing training? Risk of respiratory artefacts in functional connectivity-based real-time fMRI neurofeedback |
| 85 | 104 | Koush, Yury; Meskaldji, Djalel- E.; Pichon, Swann; Rey, Gwladys; Rieger, Sebastian W.; Linden, David E. J.; Van de Ville, Dimitri; Vuilleumier, Patrik; Scharnowski, Frank | 2017 | Learning Control Over Emotion Networks Through Connectivity-Based Neurofeedback | (Koush et al., 2017) |
| 86 | 105 | Amano, Kaoru; Shibata, Kazuhisa; Kawato, Mitsuo; Sasaki, Yuka; Watanabe, Takeo | 2016 | Learning to Associate Orientation with Color in Early Visual Areas by Associative Decoded fMRI Neurofeedback | (Amano et al., 2016) |
| 87 | 106 | Zhang, Yuan; Zhang, Qiong; Wang, Jiayuan; Zhou, Menghan; Qing, Yanan; Zou, Haochen; Li, Jianfu; Yang, Chenghui; Becker, Benjamin; Kendrick, Keith M.; Yao, Shuxia | 2023 | "Listen to your heart": A novel interoceptive strategy for real-time fMRI neurofeedback training of anterior insula activity | (Y. Zhang et al., 2023) |
| 88 | 107 | Young, Kymberly D.; Prause, Nicole; Lazzaro, Sarah; Siegle, Greg J. | 2020 | Low Cost MR Compatible Haptic Stimulation with Application to fMRI Neurofeedback | (Young et al., 2020) |
| 89 | 108 | Robineau, Fabien; Meskaldji, Djalel E.; | 2017 | Maintenance of Voluntary Self- | (Robineau et al., 2017) |

| | | | | | |
|---|---|---|---|---|---|
| | | Koush, Yury; Rieger, Sebastian W.; Mermoud, Christophe; Morgenthaler, Stephan; Van De Ville, Dimitri; Vuilleumier, Patrik; Scharnowski, Frank | | regulation Learned through Real-Time fMRI Neurofeedback | |
| 90 | 109 | Scharnowski, Frank; Veite, Ralf; Zopf, Regine; Studer, Petra; Bock, Simon; Diedrichsen, Jorn; Goebel, Rainer; Mathiak, Klaus; Birbaumer, Niels; Weiskopf, Nikolaus | 2015 | Manipulating motor performance and memory through real-time fMRI neurofeedback | (Scharnowski et al., 2015) |
| 91 | 110 | Liu, Ning; Yu, Xueli; Yao, Li; Zhao, Xiaojie | 2018 | Mapping the Cortical Network Arising From Up-Regulated Amygdaloidal Activation Using λ-Louvain Algorithm | (Liu et al., 2018) |
| 92 | 111 | Guler, Seyhmus; Cohen, Alexander L.; Afacan, Onur; Warfield, Simon K. | 2021 | Matched neurofeedback during fMRI differentially activates reward-related circuits in active and sham groups | (Guler et al., 2021) |
| 93 | 112 | Kim, Hyun-Chul; Tegethoff, Marion; Meinlschmidt, Gunther; Stalujanis, Esther; Belardi, Angelo; Jo, Sungman; Lee, Juhyeon; Kim, Dong-Youl; Yoo, Seung-Schik; Lee, Jong-Hwan | 2019 | Mediation analysis of triple networks revealed functional feature of mindfulness from real-time fMRI neurofeedback | (H. C. Kim et al., 2019) |
| 94 | 114 | Garrison, J. R.; Saviola, F.; Morgenroth, E.; Barker, H.; Luhrs, M.; Simons, J. S.; Fernyhough, C.; Allen, P. | 2021 | Modulating medial prefrontal cortex activity using real-time fMRI neurofeedback: Effects on reality monitoring performance and associated functional connectivity | (J. R. Garrison et al., 2021) |

| | | | | | |
|---|---|---|---|---|---|
| 95 | 115 | Wang, Tianlu; Peeters, Ronald; Mantini, Dante; Gillebert, Celine R. | 2020 | Modulating the interhemispheric activity balance in the intraparietal sulcus using real-time fMRI neurofeedback: Development and proof-of-concept | (T. Wang et al., 2020) |
| 96 | 116 | Karch, Susanne; Keeser, Daniel; Huemmer, Sebastian; Paolini, Marco; Kirsch, Valerie; Karali, Temmuz; Kupka, Michael; Rauchmann, Boris-Stephan; Chrobok, Agnieszka; Blautzik, Janusch; Koller, Gabi; Ertl-Wagner, Birgit; Pogarell, Oliver | 2015 | Modulation of Craving Related Brain Responses Using Real-Time fMRI in Patients with Alcohol Use Disorder | (Karch et al., 2015) |
| 97 | 117 | Hui, Mingqi; Zhang, Hang; Ge, Ruiyang; Yao, Li; Long, Zhiying | 2014 | Modulation of functional network with real-time fMRI feedback training of right premotor cortex activity | (Hui et al., 2014) |
| 98 | 118 | Berman, Brian D.; Horovitz, Silvina G.; Hallett, Mark | 2013 | Modulation of functionally localized right insular cortex activity using real-time fMRI-based neurofeedback | (Berman et al., 2013) |
| 99 | 119 | Hamilton, J. Paul; Glover, Gary H.; Hsu, Jung-Jiin; Johnson, Rebecca F.; Gotlib, Ian H. | 2011 | Modulation of Subgenual Anterior Cingulate Cortex Activity With Real-Time Neurofeedback | (Hamilton et al., 2011) |
| 100 | 120 | Zich, Catharina; Johnstone, Nicola; Luhrs, Michael D.; Lisk, Stephen T.; Haller, Simone P. W.; Lipp, Annalisa; Lau, Jennifer Y. F.; Kadosh, Kathrin Cohen | 2020 | Modulatory effects of dynamic fMRI-based neurofeedback on emotion regulation networks in adolescent females | (Zich et al., 2020) |
| 101 | 121 | Paret, Christian; Zaehringer, Jenny; Ruf, Matthias; Gerchen, Martin Fungisai; Mall, | 2018 | Monitoring and control of amygdala neurofeedback involves | (Paret et al., 2018) |

| | | | | | |
|---|---|---|---|---|---|
| | | Stephanie; Hendler, Talma; Schmahl, Christian; Ende, Gabriele | | distributed information processing in the human brain | |
| 102 | 122 | Pamplona, Gustavo S. P.; Heldner, Jennifer; Langner, Robert; Koush, Yury; Michels, Lars; Ionta, Silvio; Scharnowski, Frank; Salmon, Carlos E. G. | 2020 | Network-based fMRI-neurofeedback training of sustained attention | (Pamplona et al., 2020) |
| 103 | 126 | Nicholson, Andrew A.; Rabellino, Daniela; Densmore, Maria; Frewen, Paul A.; Paret, Christian; Kluetsch, Rosemarie; Schmahl, Christian; Theberge, Jean; Neufeld, Richard W. J.; McKinnon, Margaret C.; Reiss, Jim; Jetly, Rakesh; Lanius, Ruth A. | 2017 | The neurobiology of emotion regulation in posttraumatic stress disorder: Amygdala downregulation via real-time fMRI neurofeedback | (Nicholson et al., 2017) |
| 104 | 127 | Quevedo, Karina; Liu, Guanmin; Teoh, Jia Yuan; Ghosh, Satrajit; Zeffiro, Thomas; Ahrweiler, Natasha; Zhang, Na; Wedan, Riley; Oh, Sewon; Guercio, Guerson; Paret, Christian | 2019 | Neurofeedback and neuroplasticity of visual self-processing in depressed and healthy adolescents: A preliminary study | (Quevedo et al., 2019) |
| 105 | 128 | Yoo, Seung-Schik; Lee, Jong-Hwan; O'Leary, Heather; Panych, Lawrence P.; Jolesz, Ferenc A. | 2008 | Neurofeedback fMRI-mediated learning and consolidation of regional brain activation during motor imagery | (S.-S. Yoo et al., 2008) |
| 106 | 129 | Rance, M.; Zhao, Z.; Zaboski, B.; Kichuk, S. A.; Romaker, E.; Koller, W. N.; Walsh, C.; Harris-Starling, C.; Wasylink, S.; Adams, T., Jr.; Gruner, P.; Pittenger, C.; Hampson, M. | 2023 | Neurofeedback for obsessive compulsive disorder: A randomized, double-blind trial | (Rance et al., 2023) |
| 107 | 130 | Debettencourt, Megan T.; Turk-Browne, Nicholas B.; Norman, Kenneth A. | 2019 | Neurofeedback helps to reveal a relationship between context | (Debettencourt et al., 2019) |

| | | | | | |
|---|---|---|---|---|---|
| | | | | reinstatement and memory retrieval | |
| 108 | 131 | Lee, Dongha; Jang, Changwon; Park, Hae-Jeong | 2019 | Neurofeedback learning for mental practice rather than repetitive practice improves neural pattern consistency and functional network efficiency in the subsequent mental motor execution | (Lee et al., 2019) |
| 109 | 132 | Zweerings, Jana; Hummel, Bastian; Keller, Micha; Zvyagintsev, Mikhail; Schneider, Frank; Klasen, Martin; Mathiak, Klaus | 2019 | Neurofeedback of core language network nodes modulates connectivity with the default-mode network: A double-blind fMRI neurofeedback study on auditory verbal hallucinations | (Zweerings et al., 2019) |
| 110 | 133 | Rance, Mariela; Ruttorf, Michaele; Nees, Frauke; Schad, Lothar R.; Flor, Herta | 2014 | Neurofeedback of the difference in activation of the anterior cingulate cortex and posterior insular cortex: two functionally connected areas in the processing of pain | (Rance et al., 2014a) |
| 111 | 135 | Ihssen, Niklas; Sokunbi, Moses O.; Lawrence, Andrew D.; Lawrence, Natalia S.; Linden, David E. J. | 2017 | Neurofeedback of visual food cue reactivity: a potential avenue to alter incentive sensitization and craving | (Ihssen et al., 2017) |
| 112 | 136 | Subramanian, Leena; Skottnik, Leon; Cox, W. Miles; Luhrs, Michael; McNamara, Rachel; Hood, Kerry; Watson, Gareth; Whittaker, Joseph R.; Williams, Angharad N.; Sakhuja, Raman; Ihssen, Niklas; Goebel, Rainer; | 2021 | Neurofeedback Training versus Treatment-as-Usual for Alcohol Dependence: Results of an Early-Phase Randomized Controlled Trial and Neuroimaging Correlates | (Subramanian et al., 2021) |

| | | | | | |
|---|---|---|---|---|---|
| | | Playle, Rebecca; Linden, David E. J. | | | |
| 113 | 137 | Yu, Xiaoqian; Cohen, Zsofia; Tsuchiyagaito, Aki; Cochran, Gabriella; Aupperle, Robin; Stewart, Jennifer; Singh, Manpreet; Misaki, Masaya; Bodurka, Jerzy; Paulus, Martin; Kirlic, Namik | 2022 | Neurofeedback-Augmented Mindfulness Training Elicits Distinct Responses in the Subregions of the Insular Cortex in Healthy Adolescents | (Yu et al., 2022) |
| 114 | 138 | Pereira, Daniela Jardim; Sayal, Alexandre; Pereira, Joao; Morais, Sofia; Macedo, Antonio; Direito, Bruno; Castelo-Branco, Miguel | 2023 | Neurofeedback-dependent influence of the ventral striatum using a working memory paradigm targeting the dorsolateral prefrontal cortex | (D. J. Pereira et al., 2023) |
| 115 | 139 | Tinaz, Sule; Kamel, Serageldin; Aravala, Sai S.; Elfil, Mohamed; Bayoumi, Ahmed; Patel, Amar; Scheinost, Dustin; Sinha, Rajita; Hampson, Michelle | 2022 | Neurofeedback-guided kinesthetic motor imagery training in Parkinson's disease: Randomized trial | (Tinaz et al., 2022) |
| 116 | 140 | Sulzer, James; Sitaram, Ranganatha; Blefari, Maria Laura; Kollias, Spyros; Birbaumer, Niels; Stephan, Klaas Enno; Luft, Andreas; Gassert, Roger | 2013 | Neurofeedback-mediated self-regulation of the dopaminergic midbrain | (Sulzer et al., 2013) |
| 117 | 141 | Johnston, S. J.; Boehm, S. G.; Healy, D.; Goebel, R.; Linden, D. E. J. | 2010 | Neurofeedback: A promising tool for the self-regulation of emotion networks | (S. J. Johnston et al., 2010) |
| 118 | 142 | Bressler, Ruben Andreas; Raible, Sophie; Luhrs, Michael; Tier, Ralph; Goebel, Rainer; Linden, David E. | 2023 | No threat: Emotion regulation neurofeedback for police special forces recruits | (Bressler et al., 2023) |
| 119 | 144 | Scheinost, D.; Stoica, T.; Saksa, J.; Papademetris, X.; Constable, R. T.; Pittenger, C.; Hampson, M. | 2013 | Orbitofrontal cortex neurofeedback produces lasting changes in contamination anxiety and | (Scheinost et al., 2013) |

| | | | | | |
|---|---|---|---|---|---|
| | | | | resting-state connectivity | |
| 120 | 145 | Zhang, Suyi; Yoshida, Wako; Mano, Hiroaki; Yanagisawa, Takufumi; Mancini, Flavia; Shibata, Kazuhisa; Kawato, Mitsuo; Seymour, Ben | 2020 | Pain Control by Co-adaptive Learning in a Brain-Machine Interface | (S. Zhang et al., 2020) |
| 121 | 146 | Shibata, Kazuhisa; Watanabe, Takeo; Sasaki, Yuka; Kawato, Mitsuo | 2011 | Perceptual Learning Incepted by Decoded fMRI Neurofeedback Without Stimulus Presentation | (Shibata et al., 2011) |
| 122 | 147 | Weaver, Shelby S.; Birn, Rasmus M.; Cisler, Josh M. | 2020 | A Pilot Adaptive Neurofeedback Investigation of the Neural Mechanisms of Implicit Emotion Regulation Among Women With PTSD | (Weaver et al., 2020) |
| 123 | 149 | Tsuchiyagaito, Aki; Misaki, Masaya; Zoubi, Obada Al; Paulus, Martin; Bodurka, Jerzy; Tulsa, Investigators | 2021 | Prevent breaking bad: A proof of concept study of rebalancing the brain's rumination circuit with real-time fMRI functional connectivity neurofeedback | (Tsuchiyagaito et al., 2021) |
| 124 | 151 | Young, Kymberly D.; Siegle, Greg J.; Zotev, Vadim; Phillips, Raquel; Misaki, Masaya; Yuan, Han; Drevets, Wayne C.; Bodurka, Jerzy | 2017 | Randomized Clinical Trial of Real-Time fMRI Amygdala Neurofeedback for Major Depressive Disorder: Effectson Symptoms and Autobiographical Memory Recall | (Young et al., 2017) |
| 125 | 152 | Sukhodolsky, Denis G.; Walsh, Christopher; Koller, William N.; Eilbott, Jeffrey; Rance, Mariela; Fulbright, Robert K.; Zhao, Zhiying; Bloch, Michael H.; King, Robert; Leckman, James F.; Scheinost, | 2020 | Randomized, Sham-Controlled Trial of Real-Time Functional Magnetic Resonance Imaging Neurofeedback for Tics in Adolescents With Tourette Syndrome | (Sukhodolsky et al., 2020) |

| | | | | | |
|---|---|---|---|---|---|
| | | Dustin; Pittman, Brian; Hampson, Michelle | | | |
| 126 | 153 | Van den Boom, Max Alexander; Jansma, Johan Martijn; Ramsey, Nick Franciscus | 2018 | Rapid acquisition of dynamic control over DLPFC using real-time fMRI feedback | (Van den Boom et al., 2018) |
| 127 | 154 | Horovitz, S. G.; Berman, B. D.; Hallett, M. | 2010 | Real time BOLD functional MRI neuro-feedback affects functional connectivity | (Horovitz et al., 2010) |
| 128 | 155 | Rance, Mariela; Ruttorf, Michaela; Nees, Frauke; Schad, Lothar Rudi; Flor, Herta | 2014 | Real Time fMRI Feedback of the Anterior Cingulate and Posterior Insular Cortex in the Processing of Pain | (Rance et al., 2014b) |
| 129 | 156 | Hampson, Michelle; Stoica, Teodora; Saksa, John; Scheinost, Dustin; Qiu, Maolin; Bhawnani, Jitendra; Pittenger, Christopher; Papademetris, Xenophon; Constable, Todd | 2012 | Real-time fMRI Biofeedback Targeting the Orbitofrontal Cortex for Contamination Anxiety | (Hampson et al., 2012) |
| 130 | 157 | Sokunbi, Moses O.; Linden, David E. J.; Habes, Isabelle; Johnston, Stephen; Ihssen, Niklas | 2014 | Real-time fMRI brain-computer interface: development of a "motivational feedback" subsystem for the regulation of visual cue reactivity | (Sokunbi et al., 2014) |
| 131 | 158 | Okano, Kana; Bauer, Clemens C. C.; Ghosh, Satrajit S.; Lee, Yoon Ji; Melero, Helena; de los Angeles, Carlo; Nestor, Paul G.; del Re, Elisabetta C.; Northoff, Georg; Whitfield-Gabrieli, Susan; Niznikiewicz, Margaret A. | 2020 | Real-time fMRI feedback impacts brain activation, results in auditory hallucinations reduction: Part 1: Superior temporal gyrus -Preliminary evidence | (Okano et al., 2020) |
| 132 | 159 | Haller, Sven; Birbaumer, Niels; Veit, Ralf | 2010 | Real-time fMRI feedback training | (Haller et al., 2010) |

| | | | | | |
|---|---|---|---|---|---|
| | | | | may improve chronic tinnitus | |
| 133 | 160 | Tsuchiyagaito, Aki; Misaki, Masaya; Kirlic, Namik; Yu, Xiaoqian; Sanchez, Stella M.; Cochran, Gabe; Stewart, Jennifer L.; Smith, Ryan; Fitzgerald, Kate D.; Rohan, Michael L.; Paulus, Martin P.; Guinjoan, Salvador M. | 2023 | Real-Time fMRI Functional Connectivity Neurofeedback Reducing Repetitive Negative Thinking in Depression: A Double-Blind, Randomized, Sham-Controlled Proof-of-Concept Trial | (Tsuchiyagaito et al., 2023) |
| 134 | 161 | Garrison, Kathleen A.; Scheinost, Dustin; Worhunsky, Patrick D.; Elwafi, Hani M.; Thornhill, Thomas A.; Thompson, Evan; Saron, Clifford; Desbordes, Gaelle; Kober, Hedy; Hampson, Michelle; Gray, Jeremy R.; Constable, R. Todd; Papademetris, Xenophon; Brewer, Judson A. | 2013 | Real-time fMRI links subjective experience with brain activity during focused attention | (K. A. Garrison et al., 2013) |
| 135 | 162 | Mel'nikov, Mikhail Ye; Bezmaternykh, Dmitriy D.; Savelov, Andrey A.; Petrovskiy, Evgeniy D.; Kozlova, Lyudmila I.; Natarova, Kira A.; Larina, Tatiana D.; Andamova, Tatiana M.; Zvyagintsev, Mikhail; Shtark, Mark B.; Mathiak, Klaus | 2023 | Real-time fMRI neurofeedback compared to cognitive behavioral therapy in a pilot study for the treatment of mild and moderate depression | (Mel’nikov et al., 2023) |
| 136 | 163 | Alegria, Analucia A.; Wulff, Melanie; Brinson, Helen; Barker, Gareth J.; Norman, Luke J.; Brandeis, Daniel; Stahl, Daniel; David, Anthony S.; Taylor, Eric; Giampietro, Vincent; Rubia, Katya | 2017 | Real-Time fMRI Neurofeedback in Adolescents with Attention Deficit Hyperactivity Disorder | (Alegria et al., 2017) |
| 137 | 164 | Karch, S.; Paolini, M.; Gschwendtner, S.; Jeanty, H.; | 2019 | Real-Time fMRI Neurofeedback in Patients With | (Karch et al., 2019) |

| | | | | | |
|---|---|---|---|---|---|
| | | Reckenfelderbäumer, A.; Yaseen, O.; Maywald, M.; Fuchs, C.; Rauchmann, B. S.; Chrobok, A.; Rabenstein, A.; Ertl-Wagner, B.; Pogarell, O.; Keeser, D.; Rüther, T. | | Tobacco Use Disorder During Smoking Cessation: Functional Differences and Implications of the First Training Session in Regard to Future Abstinence or Relapse | |
| 138 | 165 | Bauer, Clemens C. C.; Okano, Kana; Gosh, Satrajit S.; Lee, Yoon Ji; Melero, Helena; de los Angeles, Carlo; Nestor, Paul G.; del Re, Elisabetta C.; Northoff, Georg; Niznikiewicz, Margaret A.; Whitfield-Gabrieli, Susan | 2020 | Real-time fMRI neurofeedback reduces auditory hallucinations and modulates resting state connectivity of involved brain regions: Part 2: Default mode network - preliminary evidence | (Bauer et al., 2020) |
| 139 | 166 | Orlov, N. D.; Giampietro, V.; O'Daly, O.; Lam, S. L.; Barker, G. J.; Rubia, K.; McGuire, P.; Shergill, S. S.; Allen, P. | 2018 | Real-time fMRI neurofeedback to down-regulate superior temporal gyrus activity in patients with schizophrenia and auditory hallucinations: a proof-of-concept study | (Orlov et al., 2018) |
| 140 | 167 | Li, Xiaodong; Li, Zhonglin; Zou, Zhi; Wu, Xiaolin; Gao, Hui; Wang, Caiyun; Zhou, Jing; Qi, Fei; Zhang, Miao; He, Junya; Qi, Xin; Yan, Fengshan; Dou, Shewei; Zhang, Hongju; Tong, Li; Li, Yongli | 2022 | Real-Time fMRI Neurofeedback Training Changes Brain Degree Centrality and Improves Sleep in Chronic Insomnia Disorder: A Resting-State fMRI Study | (X. Li et al., 2022) |
| 141 | 168 | Young, Kymberly D.; Zotev, Vadim; Phillips, Raquel; Misaki, Masaya; Yuan, Han; Drevets, Wayne C.; Bodurka, Jerzy | 2014 | Real-Time fMRI Neurofeedback Training of Amygdala Activity in Patients with Major Depressive Disorder | (Young et al., 2014) |
| 142 | 169 | Kohl, Simon H.; Veit, Ralf; Spetter, Maartje S.; Guenther, Astrid; | 2019 | Real-time fMRI neurofeedback training to improve | (Kohl et al., 2019) |

| | | | | | |
|---|---|---|---|---|---|
| | | Rina, Andriani; Luehrs, Michael; Birbaumer, Niels; Preissl, Hubert; Hallschmid, Manfred | | eating behavior by self-regulation of the dorsolateral prefrontal cortex: A randomized controlled trial in overweight and obese subjects | |
| 143 | 170 | Gerin, Mattia I.; Fichtenholtz, Harlan; Roy, Alicia; Walsh, Christopher J.; Krystal, John H.; Southwick, Steven; Hampson, Michelle | 2016 | Real-Time fMRI Neurofeedback with War Veterans with Chronic PTSD: A Feasibility Study | (Gerin et al., 2016) |
| 144 | 172 | Krause, Florian; Benjamins, Caroline; Luhrs, Michael; Eck, Judith; Noirhomme, Quentin; Rosenke, Mona; Brunheim, Sascha; Sorger, Bettina; Goebel, Rainer | 2017 | Real-time fMRI-based self-regulation of brain activation across different visual feedback presentations | (Krause et al., 2017) |
| 145 | 173 | Zhao, Zhiying; Yao, Shuxia; Li, Keshuang; Sindermann, Cornelia; Zhou, Feng; Zhao, Weihua; Li, Jianfu; Luhrs, Michael; Goebel, Rainer; Kendrick, Keith M.; Becker, Benjamin | 2019 | Real-Time Functional Connectivity-Informed Neurofeedback of Amygdala-Frontal Pathways Reduces Anxiety | (Z. Zhao et al., 2019) |
| 146 | 175 | Kirsch, Martina; Gruber, Isabella; Ruf, Matthias; Kiefer, Falk; Kirsch, Peter | 2016 | Real-time functional magnetic resonance imaging neurofeedback can reduce striatal cue-reactivity to alcohol stimuli | (Kirsch et al., 2016) |
| 147 | 176 | Subramanian, Leena; Hindle, John V.; Johnston, Stephen; Roberts, Mark V.; Husain, Masud; Goebel, Rainer; Linden, David | 2011 | Real-Time Functional Magnetic Resonance Imaging Neurofeedback for Treatment of Parkinson's Disease | (Subramanian et al., 2011) |
| 148 | 177 | Compere, Laurie; Siegle, Greg J.; Lazzaro, Sair; Strege, Marlene; Canovali, Gia; Barb, Scott; | 2023 | Real-time functional magnetic resonance imaging neurofeedback training of | (Compere et al., 2023) |

| | | | | | |
|---|---|---|---|---|---|
| | | Huppert, Theodore; Young, Kymberly | | amygdala upregulation increases affective flexibility in depression | |
| 149 | 178 | Mennen, Anne C.; Nastase, Samuel A.; Yeshurun, Yaara; Hasson, Uri; Norman, Kenneth A. | 2022 | Real-time neurofeedback to alter interpretations of a naturalistic narrative | (Mennen et al., 2022) |
| 150 | 179 | Bruehl, Annette Beatrix; Scherpiet, Sigrid; Sulzer, James; Staempfli, Philipp; Seifritz, Erich; Herwig, Uwe | 2014 | Real-time Neurofeedback Using Functional MRI Could Improve Down-Regulation of Amygdala Activity During Emotional Stimulation: A Proof-of-Concept Study | (Bruehl et al., 2014) |
| 151 | 180 | Linden, David E. J.; Habes, Isabelle; Johnston, Stephen J.; Linden, Stefanie; Tatineni, Ranjit; Subramanian, Leena; Sorger, Bettina; Healy, David; Goebel, Rainer | 2012 | Real-Time Self-Regulation of Emotion Networks in Patients with Depression | (Linden et al., 2012) |
| 152 | 181 | Van De Ville, Dimitri; Jhooti, Permi; Haas, Tanja; Kopel, Rotem; Lovblad, Karl-Olof; Scheffler, Klaus; Haller, Sven | 2012 | Recovery of the default mode network after demanding neurofeedback training occurs in spatio-temporally segregated subnetworks | (Van De Ville et al., 2012) |
| 153 | 182 | Zhang, Jiahe; Raya, Jovicarole; Morfini, Francesca; Urban, Zoi; Pagliaccio, David; Yendiki, Anastasia; Auerbach, Randy P.; Bauer, Clemens C. C.; Whitfield-Gabrieli, Susan | 2023 | Reducing default mode network connectivity with mindfulness-based fMRI neurofeedback: a pilot study among adolescents with affective disorder history | (J. Zhang et al., 2023) |
| 154 | 184 | Caria, Andrea; Veit, Ralf; Sitaram, Ranganatha; Lotze, Martin; Weiskopf, Nikolaus; Grodd, | 2007 | Regulation of anterior insular cortex activity using real-time fMRI | (Caria et al., 2007) |

| | | | | | |
|---|---|---|---|---|---|
| | | Wolfgang; Birbaumer, Niels | | | |
| 155 | 185 | Klöbl, M.; Michenthaler, P.; Godbersen, G. M.; Robinson, S.; Hahn, A.; Lanzenberger, R. | 2020 | Reinforcement and Punishment Shape the Learning Dynamics in fMRI Neurofeedback | (Klöbl et al., 2020) |
| 156 | 186 | Yuan, Han; Young, Kymberly D.; Phillips, Raquel; Zotev, Vadim; Misaki, Masaya; Bodurka, Jerzy | 2014 | Resting-state functional connectivity modulation and sustained changes after real-time functional magnetic resonance imaging neurofeedback training in depression | (Yuan et al., 2014) |
| 157 | 187 | Zweerings, Jana; Sarkheil, Pegah; Keller, Micha; Dyck, Miriam; Klasen, Martin; Becker, Benjamin; Gaebler, Arnim J.; Ibrahim, Camellia N.; Turetsky, Bruce I.; Zvyagintsev, Mikhail; Flatten, Guido; Mathiak, Klaus | 2020 | Rt-fMRI neurofeedback-guided cognitive reappraisal training modulates amygdala responsivity in posttraumatic stress disorder | (Zweerings et al., 2020) |
| 158 | 188 | Fede, Samantha J.; Kisner, Mallory A.; Dean, Sarah F.; Kerich, Mike; Roopchansingh, Vinai; Diazgranados, Nancy; Momenan, Reza | 2023 | Selecting an optimal real-time fMRI neurofeedback method for alcohol craving control training | (Fede et al., 2023) |
| 159 | 189 | Jaeckle, Tanja; Williams, Steven C. R.; Barker, Gareth J.; Basilio, Rodrigo; Carr, Ewan; Goldsmith, Kimberley; Colasanti, Alessandro; Giampietro, Vincent; Cleare, Anthony; Young, Allan H.; Moll, Jorge; Zahn, Roland | 2023 | Self-blame in major depression: a randomised pilot trial comparing fMRI neurofeedback with self-guided psychological strategies | (Jaeckle et al., 2023) |
| 160 | 190 | Sherwood, Matthew S.; Parker, Jason G.; Diller, Emily E.; Ganapathy, Subhashini; Bennett, | 2019 | Self-directed down-regulation of auditory cortex activity mediated by real-time fMRI | (Sherwood et al., 2019) |

| | | | | | |
|---|---|---|---|---|---|
| | | Kevin B.; Esquivel, Carlos R.; Nelson, Jeremy T. | | neurofeedback augments attentional processes, resting cerebral perfusion, and auditory activation | |
| 161 | 191 | Sanders, Zeena Britt; Fleming, Melanie K.; Smejka, Tom; Marzolla, Marilien C.; Zich, Catharina; Rieger, Sebastian W.; Luhrs, Michael; Goebel, Rainer; Sampaio-Baptista, Cassandra; Johansen-Berg, Heidi | 2022 | Self-modulation of motor cortex activity after stroke: a randomized controlled trial | (Sanders et al., 2022) |
| 162 | 192 | Pereira, Joao; Direito, Bruno; Sayal, Alexandre; Ferreira, Carlos; Castelo-Branco, Miguel | 2019 | Self-Modulation of Premotor Cortex Interhemispheric Connectivity in a Real-Time Functional Magnetic Resonance Imaging Neurofeedback Study Using an Adaptive Approach | (J. Pereira et al., 2019) |
| 163 | 193 | Berman, Brian D.; Horovitz, Silvina G.; Venkataraman, Gaurav; Hallett, Mark | 2012 | Self-modulation of primary motor cortex activity with motor and motor imagery tasks using real-time fMRI-based neurofeedback | (Berman et al., 2012) |
| 164 | 194 | Li, Zhonglin; Tong, Li; Wang, Linyuan; Li, Yongli; He, Wenjie; Guan, Min; Yan, Bin | 2016 | Self-regulating positive emotion networks by feedback of multiple emotional brain states using real-time fMRI | (Z. Li, Tong, Wang, et al., 2016) |
| 165 | 195 | Zotev, Vadim; Krueger, Frank; Phillips, Raquel; Alvarez, Ruben P.; Simmons, W. Kyle; Bellgowan, Patrick; Drevets, Wayne C.; Bodurka, Jerzy | 2011 | Self-Regulation of Amygdala Activation Using Real-Time fMRI Neurofeedback | (Zotev et al., 2011) |
| 166 | 196 | Buyukturkoglu, Korhan; Roettgers, Hans; Sommer, Jens; | 2015 | Self-Regulation of Anterior Insula with Real-Time | (Buyukturkoglu et al., 2015) |

| | | | | | |
|---|---|---|---|---|---|
| | | Rana, Mohit; Dietzsch, Leonie; Arikan, Ezgi Belkis; Veit, Ralf; Malekshahi, Rahim; Kircher, Tilo; Birbaumer, Niels; Sitaram, Ranganatha; Ruiz, Sergio | | fMRI and Its Behavioral Effects in Obsessive-Compulsive Disorder: A Feasibility Study | |
| 167 | 197 | Guan, Min; Ma, Lijia; Li, Li; Yan, Bin; Zhao, Lu; Tong, Li; Dou, Shewei; Xia, Linjie; Wang, Meiyun; Shi, Dapeng | 2015 | Self-Regulation of Brain Activity in Patients with Postherpetic Neuralgia: A Double-Blind Randomized Study Using Real-Time fMRI Neurofeedback | (Guan et al., 2015) |
| 168 | 198 | Robineau, F.; Rieger, S. W.; Mermoud, C.; Pichon, S.; Koush, Y.; Van De Ville, D.; Vuilleumier, P.; Scharnowski, F. | 2014 | Self-regulation of inter-hemispheric visual cortex balance through real-time fMRI neurofeedback training | (Robineau et al., 2014) |
| 169 | 199 | Sreedharan, Sujesh; Chandran, Anuvitha; Yanamala, Vijay Raj; Sylaja, P. N.; Kesavadas, Chandrasekharan; Sitaram, Ranganatha | 2020 | Self-regulation of language areas using real-time functional MRI in stroke patients with expressive aphasia | (Sreedharan et al., 2020) |
| 170 | 201 | Rota, Giuseppina; Sitaram, Ranganatha; Veit, Ralf; Erb, Michael; Weiskopf, Nikolaus; Dogil, Grzegorz; Birbaumer, Niels | 2009 | Self-Regulation of Regional Cortical Activity Using Real-Time fMRI: The Right Inferior Frontal Gyrus and Linguistic Processing | (Rota et al., 2009) |
| 171 | 202 | Krause, Florian; Kogias, Nikos; Krentz, Martin; Luhrs, Michael; Goebel, Rainer; Hermans, Erno J. | 2021 | Self-regulation of stress-related large-scale brain network balance using real-time fMRI neurofeedback | (Krause et al., 2021) |
| 172 | 203 | Lawrence, Emma J.; Su, Li; Barker, Gareth J.; Medford, Nick; Dalton, Jeffrey; Williams, Steve C. R.; Birbaumer, Niels; Veit, Ralf; | 2014 | Self-regulation of the anterior insula: Reinforcement learning using real-time fMRI neurofeedback | (Lawrence et al., 2014) |

| | | | | | |
|---|---|---|---|---|---|
| | | Ranganatha, Sitaram; Bodurka, Jerzy; Brammer, Michael; Giampietro, Vincent; David, Anthony S. | | | |
| 173 | 204 | Kirschner, Matthias; Sladky, Ronald; Haugg, Amelie; Stampfli, Philipp; Jehli, Elisabeth; Hodel, Martina; Engeli, Etna; Hosli, Sarah; Baumgartner, Markus R.; Sulzer, James; Huys, Quentin J. M.; Seifritz, Erich; Quednow, Boris B.; Scharnowski, Frank; Herdener, Marcus | 2018 | Self-regulation of the dopaminergic reward circuit in cocaine users with mental imagery and neurofeedback | (Kirschner et al., 2018) |
| 174 | 205 | Pereira, Jaime A.; Sepulveda, Pradyumna; Rana, Mohit; Montalba, Cristian; Tejos, Cristian; Torres, Rafael; Sitaram, Ranganatha; Ruiz, Sergio | 2019 | Self-Regulation of the Fusiform Face Area in Autism Spectrum: A Feasibility Study With Real-Time fMRI Neurofeedback | (J. A. Pereira et al., 2019) |
| 175 | 206 | Kirlic, Namik; Cohen, Zsofia P.; Tsuchiyagaito, Aki; Misaki, Masaya; McDermott, Timothy J.; Aupperle, Robin L.; Stewart, Jennifer L.; Singh, Manpreet K.; Paulus, Martin P.; Bodurka, Jerzy | 2022 | Self-regulation of the posterior cingulate cortex with real-time fMRI neurofeedback augmented mindfulness training in healthy adolescents: A nonrandomized feasibility study | (Kirlic et al., 2022) |
| 176 | 207 | Mayeli, Ahmad; Misaki, Masaya; Zotev, Vadim; Tsuchiyagaito, Aki; Al Zoubi, Obada; Phillips, Raquel; Smith, Jared; Stewart, Jennifer L.; Refai, Hazem; Paulus, Martin P.; Bodurka, Jerzy | 2020 | Self-regulation of ventromedial prefrontal cortex activation using real-time fMRI neurofeedback-Influence of default mode network | (Mayeli et al., 2020) |
| 177 | 208 | Haugg, Amelie; Frei, Nada; Menghini, Milena; Stutz, Felizia; Steinegger, Sara; | 2023 | Self-regulation of visual word form area activation with real-time fMRI neurofeedback | (Haugg et al., 2023) |

| | | | | | |
|---|---|---|---|---|---|
| | | Rothlisberger, Martina; Brem, Silvia | | | |
| 178 | 209 | Ciarlo, Assunta; Russo, Andrea G.; Ponticorvo, Sara; di Salle, Francesco; Luhrs, Michael; Goebel, Rainer; Esposito, Fabrizio | 2022 | Semantic fMRI neurofeedback: a multi-subject study at 3 tesla | (Ciarlo et al., 2022) |
| 179 | 210 | MacDuffie, Katherine E.; MacInnes, Jeff; Dickerson, Kathryn C.; Eddington, Kari M.; Strauman, Timothy J.; Adcock, R. Alison | 2018 | Single session real-time fMRI neurofeedback has a lasting impact on cognitive behavioral therapy strategies | (MacDuffie et al., 2018) |
| 180 | 212 | Mathiak, Krystyna A.; Alawi, Eliza M.; Koush, Yury; Dyck, Miriam; Cordes, Julia S.; Gaber, Tilman J.; Zepf, Florian D.; Palomero-Gallagher, Nicola; Sarkheil, Pegah; Bergert, Susanne; Zvyagintsev, Mikhail; Mathiak, Klaus | 2015 | Social reward improves the voluntary control over localized brain activity in fMRI-based neurofeedback training | (Mathiak et al., 2015) |
| 181 | 213 | Papoutsi, Marina; Weiskopf, Nikolaus; Langbehn, Douglas; Reilmann, Ralf; Rees, Geraint; Tabrizi, Sarah J. | 2018 | Stimulating neural plasticity with real-time fMRI neurofeedback in Huntington's disease: A proof of concept study | (Papoutsi et al., 2018) |
| 182 | 214 | Marins, T.; Rodrigues, E. C.; Bortolini, T.; Melo, Bruno; Moll, J.; Tovar-Moll, F. | 2019 | Structural and functional connectivity changes in response to short-term neurofeedback training with motor imagery | (T. Marins et al., 2019) |
| 183 | 216 | Canterberry, Melanie; Hanlon, Colleen A.; Hartwell, Karen J.; Li, Xingbao; Owens, Max; LeMatty, Todd; Prisciandaro, James J.; Borckardt, Jeffrey; Saladin, Michael E.; Brady, Kathleen T.; George, Mark S. | 2013 | Sustained Reduction of Nicotine Craving With Real-Time Neurofeedback: Exploring the Role of Severity of Dependence | (Canterberry et al., 2013) |

| | | | | | |
|---|---|---|---|---|---|
| 184 | 217 | Direito, Bruno; Lima, Joao; Simoes, Marco; Sayal, Alexandre; Sousa, Teresa; Luhrs, Michael; Ferreira, Carlos; Castelo-Branco, Miguel | 2019 | Targeting dynamic facial processing mechanisms in superior temporal sulcus using a novel fMRI neurofeedback target | (Direito et al., 2019) |
| 185 | 218 | Mehler, David M. A.; Sokunbi, Moses O.; Habes, Isabelle; Barawi, Kali; Subramanian, Leena; Range, Maxence; Evans, John; Hoods, Kerenza; Luhrs, Michael; Keedwell, Paul; Goebel, Rainer; Linden, David E. J. | 2018 | Targeting the affective brain-a randomized controlled trial of real-time fMRI neurofeedback in patients with depression | (Mehler et al., 2018) |
| 186 | 219 | Dyck, Miriam S.; Mathiak, Krystyna A.; Bergert, Susanne; Sarkheil, Pegah; Koush, Yury; Alawi, Eliza M.; Zvyagintsev, Mikhail; Gaebler, Arnim J.; Shergill, Sukhi S.; Mathiak, Klaus | 2016 | Targeting Treatment-Resistant Auditory Verbal Hallucinations in Schizophrenia with fMRI-Based Neurofeedback - Exploring Different Cases of Schizophrenia | (Dyck et al., 2016) |
| 187 | 220 | Chung, Young In; White, Roisin; Geier, Charles F. F.; Johnston, Stephen J. J.; Smyth, Joshua M. M.; Delgado, Mauricio R. R.; McKee, Sherry A. A.; Wilson, Stephen J. J. | 2023 | Testing the efficacy of real-time fMRI neurofeedback for training people who smoke daily to upregulate neural responses to nondrug rewards | (Chung et al., 2023) |
| 188 | 221 | Rance, Mariela; Walsh, Christopher; Sukhodolsky, Denis G.; Pittman, Brian; Qiu, Maolin; Kichuk, Stephen A.; Wasylink, Suzanne; Koller, William N.; Bloch, Michael; Gruner, Patricia; Scheinost, Dustin; Pittenger, Christopher; Hampson, Michelle | 2018 | Time course of clinical change following neurofeedback | Time course of clinical change following neurofeedback |
| 189 | 222 | Kaas, Amanda; Goebel, Rainer; | 2019 | Topographic Somatosensory | (Kaas et al., 2019) |

| | | | | | |
|---|---|---|---|---|---|
| | | Valente, Giancarlo; Sorger, Bettina | | Imagery for Real-Time fMRI Brain-Computer Interfacing | |
| 190 | 223 | Russo, Andrea G.; Luhrs, Michael; Di Salle, Francesco; Esposito, Fabrizio; Goebel, Rainer | 2021 | Towards semantic fMRI neurofeedback: navigating among mental states using real-time representational similarity analysis | (Russo et al., 2021) |
| 191 | 224 | Auer, Tibor; Schweizer, Renate; Frahm, Jens | 2015 | Training efficiency and transfer success in an extended real-time functional MRI neurofeedback training of the somatomotor cortex of healthy subjects | (Auer et al., 2015) |
| 192 | 225 | Herwig, U.; Lutz, J.; Scherpiet, S.; Scheerer, H.; Kohlberg, J.; Opialla, S.; Preuss, A.; Steiger, V. R.; Sulzer, J.; Weidt, S.; Staempfli, P.; Rufer, M.; Seifritz, E.; Jancke, L.; Bruehl, A. B. | 2019 | Training emotion regulation through real-time fMRI neurofeedback of amygdala activity | (Herwig et al., 2019) |
| 193 | 226 | Lisk, Stephen; Kadosh, Kathrin Cohen; Zich, Catharina; Haller, Simone P. W.; Lau, Jennifer Y. F. | 2020 | Training negative connectivity patterns between the dorsolateral prefrontal cortex and amygdala through fMRI-based neurofeedback to target adolescent socially-avoidant behaviour | (Lisk et al., 2020) |
| 194 | 227 | Direito, Bruno; Mouga, Susana; Sayal, Alexandre; Simoes, Marco; Quental, Hugo; Bernardino, Ines; Playle, Rebecca; McNamara, Rachel; Linden, David E. J.; Oliveira, Guiomar; | 2021 | Training the social brain: Clinical and neural effects of an 8-week real-time functional magnetic resonance imaging neurofeedback Phase IIa Clinical Trial in Autism | (Direito, Mouga, et al., 2021) |

| | | | | | |
|---|---|---|---|---|---|
| | | Branco, Miguel Castelo | | | |
| 195 | 228 | Saxena, Abhishek; Shovestul, Bridget J.; Dudek, Emily M.; Reda, Stephanie; Venkataraman, Arun; Lamberti, J. Steven; Dodell-Feder, David | 2023 | Training volitional control of the theory of mind network with real-time fMRI neurofeedback | (Saxena et al., 2023) |
| 196 | 229 | Margolles, Pedro; Elosegi, Patxi; Mei, Ning; Soto, David | 2023 | Unconscious manipulation of conceptual representations with decoded neurofeedback impacts search behaviour | (Margolles et al., 2023) |
| 197 | 230 | Johnston, Stephen; Linden, D. E. J.; Healy, D.; Goebel, R.; Habes, I.; Boehm, S. G. | 2011 | Upregulation of emotion areas through neurofeedback with a focus on positive mood | (S. Johnston et al., 2011) |
| 198 | 231 | Al-Wasity, S.; Vogt, S.; Vuckovic, A.; Pollick, F. E. | 2021 | Upregulation of Supplementary Motor Area Activation with fMRI Neurofeedback during Motor Imagery | (Al-Wasity et al., 2021) |
| 199 | 232 | Groene, M.; Dyck, M.; Koush, Y.; Bergert, S.; Mathiak, K. A.; Alawi, E. M.; Elliott, M.; Mathiak, K. | 2015 | Upregulation of the Rostral Anterior Cingulate Cortex can Alter the Perception of Emotions: fMRI-Based Neurofeedback at 3 and 7 T | (Groene et al., 2015) |
| 200 | 233 | Rana, Mohit; Ruiz, Sergio; Sanchez Corzo, Andrea; Muehleck, Axel; Eck, Sandra; Salinas, Cesar; Zamorano, Francisco; Silva, Claudio; Rea, Massimiliano; Batra, Anil; Birbaumer, Niels; Sitaram, Ranganatha | 2020 | Use of Real-Time Functional Magnetic Resonance Imaging-Based Neurofeedback to Downregulate Insular Cortex in Nicotine-Addicted Smokers | (Rana et al., 2020) |
| 201 | 234 | Morgenroth, Elenor; Saviola, Francesca; Gilleen, James; Allen, | 2020 | Using connectivity-based real-time fMRI | (Morgenroth et al., 2020) |

| | | | | | |
|---|---|---|---|---|---|
| | | Beth; Luhrs, Michael; Eysenck, Michael W.; Allen, Paul | | neurofeedback to modulate attentional and resting state networks in people with high trait anxiety | |
| 202 | 235 | Madkhali, Yahia; Al-Wasity, Salim; Aldehmi, Norah; Pollick, Frank | 2022 | Using Real-Time fMRI Neurofeedback to Modulate M1-Cerebellum Connectivity | (Madkhali et al., 2022) |
| 203 | 236 | Robineau, Fabien; Saj, Arnaud; Neveu, Remi; Van De Ville, Dimitri; Scharnowski, Frank; Vuilleumier, Patrik | 2019 | Using real-time fMRI neurofeedback to restore right occipital cortex activity in patients with left visuo-spatial neglect: proof-of-principle and preliminary results | (Robineau et al., 2019) |
| 204 | 237 | Kadosh, Kathrin Cohen; Luo, Qiang; de Burca, Calem; Sokunbi, Moses O.; Feng, Jianfeng; Linden, David E. J.; Lau, Jennifer Y. F. | 2016 | Using real-time fMRI to influence effective connectivity in the developing emotion regulation network | (Kadosh et al., 2016) |
| 205 | 238 | Andersson, Patrik; Ragni, Flavio; Lingnau, Angelika | 2019 | Visual imagery during real-time fMRI neurofeedback from occipital and superior parietal cortex | (Andersson et al., 2019) |
| 206 | 239 | Banca, Paula; Sousa, Teresa; Duarte, Isabel Catarina; Castelo-Branco, Miguel | 2015 | Visual motion imagery neurofeedback based on the hMT+/V5 complex: evidence for a feedback-specific neural circuit involving neocortical and cerebellar regions | (Banca et al., 2015) |
| 207 | 240 | Wang, Zhiyan; Tamaki, Masako; Frank, Sebastian M.; Shibata, Kazuhisa; Worden, Michael S.; Yamada, Takashi; | 2021 | Visual perceptual learning of a primitive feature in human V1/V2 as a result of unconscious | (Z. Wang et al., 2021) |

| | | | | | |
|---|---|---|---|---|---|
| | | Kawato, Mitsuo; Sasaki, Yuka; Watanabe, Takeo | | processing, revealed by decoded functional MRI neurofeedback (DecNef) | |
| 208 | 241 | Sitaram, Ranganatha; Caria, Andrea; Veit, Ralf; Gaber, Tilman; Ruiz, Sergio; Birbaumer, Niels | 2014 | Volitional control of the anterior insula in criminal psychopaths using real-time fMRI neurofeedback: a pilot study | (Sitaram et al., 2014) |
| 209 | 242 | Sherwood, Matthew S.; Parker, Jason G.; Diller, Emily E.; Ganapathy, Subhashini; Bennett, Kevin; Nelson, Jeremy T. | 2018 | Volitional down-regulation of the primary auditory cortex via directed attention mediated by real-time fMRI neurofeedback | (Sherwood et al., 2018) |
| 210 | 243 | Ekanayake, Jinendra; Ridgway, Gerard R.; Winston, Joel S.; Feredoes, Eva; Razi, Adeel; Koush, Yury; Scharnowski, Frank; Weiskopf, Nikolaus; Rees, Geraint | 2019 | Volitional modulation of higher-order visual cortex alters human perception | (Ekanayake et al., 2019) |
| 211 | 244 | Travassos, Carolina; Sayal, Alexandre; Direito, Bruno; Castelhano, Joao; Castelo-Branco, Miguel | 2020 | Volitional Modulation of the Left DLPFC Neural Activity Based on a Pain Empathy Paradigm-A Potential Novel Therapeutic Target for Pain | (Travassos et al., 2020) |
| 212 | 245 | Li, Xingbao; Hartwell, Karen J.; Borckardt, Jeffery; Prisciandaro, James J.; Saladin, Michael E.; Morgan, Paul S.; Johnson, Kevin A.; LeMatty, Todd; Brady, Kathleen T.; George, Mark S. | 2013 | Volitional reduction of anterior cingulate cortex activity produces decreased cue craving in smoking cessation: a preliminary real-time fMRI study | (X. Li et al., 2013) |
| 213 | 246 | Spetter, Maartje S.; Malekshahi, Rahim; Birbaumer, Niels; Luhrs, Michael; van der Veer, Albert H.; Scheffler, Klaus; Spuckti, Sophia; Preissl, Hubert; Veit, | 2017 | Volitional regulation of brain responses to food stimuli in overweight and obese subjects: A real-time fMRI feedback study | (Spetter et al., 2017) |

| | | | | | |
|---|---|---|---|---|---|
| | | Ralf; Hallschmid, Manfred | | | |
| 214 | 247 | Yao, Shuxia; Becker, Benjamin; Geng, Yayuan; Zhao, Zhiying; Xu, Xiaolei; Zhao, Weihua; Ren, Peng; Kendrick, Keith M. | 2016 | Voluntary control of anterior insula and its functional connections is feedback-independent and increases pain empathy | (Yao et al., 2016) |
| 215 | 248 | Moll, Jorge; Weingartner, Julie H.; Bado, Patricia; Basilio, Rodrigo; Sato, Joao R.; Melo, Bruno R.; Bramati, Ivanei E.; de Oliveira-Souza, Ricardo; Zahn, Roland | 2014 | Voluntary Enhancement of Neural Signatures of Affiliative Emotion Using fMRI Neurofeedback | (Moll et al., 2014) |
| 216 | 249 | Vargas, Patricia; Sitaram, Ranganatha; Sepulveda, Pradyumna; Montalba, Cristian; Rana, Mohit; Torres, Rafael; Tejos, Cristian; Ruiz, Sergio | 2021 | Weighted neurofeedback facilitates greater self-regulation of functional connectivity between the primary motor area and cerebellum | (Vargas et al., 2021) |
| 217 | 250 | Sorger, Bettina; Kamp, Tabea; Weiskopf, Nikolaus; Peters, Judith Caroline; Goebel, Rainer | 2018 | When the Brain Takes 'BOLD' Steps: Real-Time fMRI Neurofeedback Can Further Enhance the Ability to Gradually Self-regulate Regional Brain Activation | (Sorger et al., 2018) |
| 218 | 251 | Zhang G., Yao, L., Zhang, H., Long, Z., Zhao, X. | 2013 | Improved working memory performance through self-regulation of dorsal lateral prefrontal cortex activation using real-time fMRI | (G. Zhang, Yao, et al., 2013) |
| 219 | 252 | Misaki, M., Phillips, R., Zotev, V., Wong, C-K., Wurfel, B.E., Krueger, F., Feldner, M., Bodurka, J. | 2018 | Real-time fMRI amygdala neurofeedback positive emotional training normalized resting-state functional connectivity in | (Misaki et al., 2018) |

| | | | | | |
|---|---|---|---|---|---|
| | | | | combat veterans with and without PTSD: a connectome-wide investigation | |
| 220 | 253 | Bray S, Shimojo S, O'Doherty JP | 2007 | Direct instrumental conditioning of neural activity using functional magnetic resonance imaging-derived reward feedback | (Bray et al., 2007) |
| 221 | 254 | Caria, A., Sitaram, R., Veit, R., Begliomini, C., & Birbaumer, N | 2010 | Volitional control of anterior insula activity modulates the response to aversive stimuli. A real-time functional magnetic resonance imaging study | (Caria et al., 2010) |
| 222 | 255 | deCharms, R. C., Christoff, K., Glover, G. H., Pauly, J. M., Whitfield, S., & Gabrieli, J. D | 2004 | Learned regulation of spatially localized brain activation using real-time fMRI | (deCharms et al., 2004) |
| 223 | 256 | deCharms, R. C., Maeda, F., Glover, G. H., Ludlow, D., Pauly, J. M., Soneji, D., Gabrieli, J. D., & Mackey, S. C | 2005 | Control over brain activation and pain learned by using real-time functional MRI | (deCharms et al., 2005) |
| 224 | 257 | Frank, S., Lee, S., Preissl, H., Schultes, B., Birbaumer, N., & Veit, R | 2012 | The obese brain athlete: self-regulation of the anterior insula in adiposity | (Frank et al., 2012) |
| 225 | 258 | McCaig, R. G., Dixon, M., Keramatian, K., Liu, I., & Christoff, K | 2011 | Improved modulation of rostrolateral prefrontal cortex using real-time fMRI training and meta-cognitive awareness | (McCaig et al., 2011) |
| 226 | 260 | Veit, R., Singh, V., Sitaram, R., Caria, A., Rauss, K., & Birbaumer, N | 2012 | Using real-time fMRI to learn voluntary regulation of the anterior insula in the presence of threatrelated stimuli | (Veit et al., 2012) |

| | | | | | |
|---|---|---|---|---|---|
| 227 | 261 | Zhao X, Zhang H, Song S, Ye Q, Guo J, Yao L | 2013 | Causal interaction following the alteration of target region activation during motor imagery training using | (X. Zhao et al., 2013) |


Al-Wasity, S., Vogt, S., Vuckovic, A., & Pollick, F. E. (2021). Upregulation of Supplementary Motor Area Activation with fMRI Neurofeedback during Motor Imagery. *ENeuro*, *8*(1). https://doi.org/doi:10.1523/eneuro.0377-18.2020

Alegria, A. A., Wulff, M., Brinson, H., Barker, G. J., Norman, L. J., Brandeis, D., Stahl, D., David, A. S., Taylor, E., Giampietro, V., & Rubia, K. (2017). Real-time fMRI neurofeedback in adolescents with attention deficit hyperactivity disorder. *Human Brain Mapping*, *38*(6), 3190–3209. https://doi.org/10.1002/hbm.23584

Amano, K., Shibata, K., Kawato, M., Sasaki, Y., & Watanabe, T. (2016). Learning to Associate Orientation with Color in Early Visual Areas by Associative Decoded fMRI Neurofeedback. *CURRENT BIOLOGY*, *26*(14), 1861–1866. https://doi.org/doi:10.1016/j.cub.2016.05.014

Andersson, P., Ragni, F., & Lingnau, A. (2019). Visual imagery during real-time fMRI neurofeedback from occipital and superior parietal cortex. *Neuroimage*, *200*, 332–343. https://doi.org/doi:10.1016/j.neuroimage.2019.06.057

Auer, T., Schweizer, R., & Frahm, J. (2015). Training efficiency and transfer success in an extended real-time functional MRI neurofeedback training of the somatomotor cortex of healthy subjects. *FRONTIERS IN HUMAN NEUROSCIENCE*, *9*. https://doi.org/doi:10.3389/fnhum.2015.00547

Bagarinao, E., Yoshida, A., Terabe, K., Kato, S., & Nakai, T. (2020). Improving Real-Time Brain State Classification of Motor Imagery Tasks During Neurofeedback Training. *FRONTIERS IN NEUROSCIENCE*, *14*. https://doi.org/doi:10.3389/fnins.2020.00623

Bagarinao, E., Yoshida, A., Ueno, M., Terabe, K., Kato, S., Isoda, H., & Nakai, T. (2018). Improved Volitional Recall of Motor-Imagery-Related Brain Activation Patterns Using Real-Time Functional MRI-Based Neurofeedback. *FRONTIERS IN HUMAN NEUROSCIENCE*, *12*. https://doi.org/doi:10.3389/fnhum.2018.00158

Banca, P., Sousa, T., Duarte, I. C., & Castelo-Branco, M. (2015). Visual motion imagery neurofeedback based on the hMT+/V5 complex: evidence for a feedback-specific neural circuit involving neocortical and cerebellar regions. *JOURNAL OF NEURAL ENGINEERING*, *12*(6). https://doi.org/doi:10.1088/1741-2560/12/6/066003

Bauer, C. C. C., Okano, K., Gosh, S. S., Lee, Y. J., Melero, H., Angeles, C. de los, Nestor, P. G., del Re, E. C., Northoff, G., Niznikiewicz, M. A., & Whitfield-Gabrieli, S. (2020). Real-time fMRI neurofeedback reduces auditory hallucinations and modulates resting state connectivity of involved brain regions: Part 2: Default Mode Network -Preliminary evidence-. *Psychiatry Research*, *284*, 112770. https://doi.org/10.1016/J.PSYCHRES.2020.112770

Berman, B. D., Horovitz, S. G., & Hallett, M. (2013). Modulation of functionally localized right insular cortex activity using real-time fMRI-based neurofeedback. *FRONTIERS IN HUMAN NEUROSCIENCE*, *7*. https://doi.org/doi:10.3389/fnhum.2013.00638

Berman, B. D., Horovitz, S. G., Venkataraman, G., & Hallett, M. (2012). Self-modulation of primary motor cortex activity with motor and motor imagery tasks using real-time fMRI-based neurofeedback. *Neuroimage*, *59*(2), 917–925. https://doi.org/doi:10.1016/j.neuroimage.2011.07.035

Blefari, M. L., Sulzer, J., Hepp-Reymond, M.-C., Kollias, S., & Gassert, R. (2015). Improvement in precision grip force control with self-modulation of primary motor cortex during motor imagery. *FRONTIERS IN BEHAVIORAL NEUROSCIENCE*, *9*. https://doi.org/doi:10.3389/fnbeh.2015.00018

Bottinger, B. W., Aggensteiner, P.-M., Hohmann, S., Heintz, S., Ruf, M., Glennon, J., Holz, N. E., Banaschewski, T., Brandeis, D., & Baumeister, S. (2023). Exploring real-time functional magnetic resonance imaging

neurofeedback in adolescents with disruptive behavior disorder and callous unemotional traits. *Journal of Affective Disorders*, *345*, 32–42. https://doi.org/doi:10.1016/j.jad.2023.10.036

Bray, S., Shimojo, S., & O'Doherty, J. P. (2007). Direct instrumental conditioning of neural activity using functional magnetic resonance imaging-derived reward feedback. *J Neurosci*, *27*(28), 7498–7507. https://doi.org/10.1523/JNEUROSCI.2118-07.2007

Bressler, R. A., Raible, S., Luhrs, M., Tier, R., Goebel, R., & Linden, D. E. (2023). No threat: Emotion regulation neurofeedback for police special forces recruits. *Neuropsychologia*, *190*, 108699. https://doi.org/doi:10.1016/j.neuropsychologia.2023.108699

Bruehl, A. B., Scherpiet, S., Sulzer, J., Staempfli, P., Seifritz, E., & Herwig, U. (2014). Real-time Neurofeedback Using Functional MRI Could Improve Down-Regulation of Amygdala Activity During Emotional Stimulation: A Proof-of-Concept Study. *BRAIN TOPOGRAPHY*, *27*(1), 138–148. https://doi.org/doi:10.1007/s10548-013-0331-9

Buyukturkoglu, K., Roettgers, H., Sommer, J., Rana, M., Dietzsch, L., Arikan, E. B., Veit, R., Malekshahi, R., Kircher, T., Birbaumer, N., Sitaram, R., & Ruiz, S. (2015). Self-Regulation of Anterior Insula with Real-Time fMRI and Its Behavioral Effects in Obsessive-Compulsive Disorder: A Feasibility Study. *PLoS One*, *10*(8). https://doi.org/doi:10.1371/journal.pone.0135872

Canterberry, M., Hanlon, C. A., Hartwell, K. J., Li, X., Owens, M., LeMatty, T., Prisciandaro, J. J., Borckardt, J., Saladin, M. E., Brady, K. T., & George, M. S. (2013). Sustained reduction of nicotine craving with real-time neurofeedback: Exploring the role of severity of dependence. *Nicotine and Tobacco Research*, *15*(12), 2120–2124. https://doi.org/10.1093/ntr/ntt122

Caria, A., Sitaram, R., Veit, R., Begliomini, C., & Birbaumer, N. (2010). Volitional control of anterior insula activity modulates the response to aversive stimuli. A real-time functional magnetic resonance imaging study. *Biol Psychiatry*, *68*(5), 425–432. https://doi.org/10.1016/j.biopsych.2010.04.020

Caria, A., Veit, R., Sitaram, R., Lotze, M., Weiskopf, N., Grodd, W., & Birbaumer, N. (2007). Regulation of anterior insular cortex activity using real-time fMRI. *Neuroimage*, *35*(3), 1238–1246. https://doi.org/doi:10.1016/j.neuroimage.2007.01.018

Chiew, M., LaConte, S. M., & Graham, S. J. (2012). Investigation of fMRI neurofeedback of differential primary motor cortex activity using kinesthetic motor imagery. *Neuroimage*, *61*(1), 21–31. https://doi.org/doi:10.1016/j.neuroimage.2012.02.053

Chung, Y. I., White, R., Geier, C. F. F., Johnston, S. J. J., Smyth, J. M. M., Delgado, M. R. R., McKee, S. A. A., & Wilson, S. J. J. (2023). Testing the efficacy of real-time fMRI neurofeedback for training people who smoke daily to upregulate neural responses to nondrug rewards. *COGNITIVE AFFECTIVE & BEHAVIORAL NEUROSCIENCE*, *23*(2), 440–456. https://doi.org/doi:10.3758/s13415-023-01070-y

Ciarlo, A., Russo, A. G., Ponticorvo, S., di Salle, F., Luhrs, M., Goebel, R., & Esposito, F. (2022). Semantic fMRI neurofeedback: a multi-subject study at 3 tesla. *JOURNAL OF NEURAL ENGINEERING*, *19*(3). https://doi.org/doi:10.1088/1741-2552/ac6f81

Collin, S. H. P., van den Broek, P. L. C., van Mourik, T., Desain, P., & Doeller, C. F. (2022). Inducing a mental context for associative memory formation with real-time fMRI neurofeedback. *SCIENTIFIC REPORTS*, *12*(1). https://doi.org/doi:10.1038/s41598-022-25799-7

Compere, L., Siegle, G. J., Lazzaro, S., Strege, M., Canovali, G., Barb, S., Huppert, T., & Young, K. (2023). Real-time functional magnetic resonance imaging neurofeedback training of amygdala upregulation increases affective flexibility in depression. *JOURNAL OF PSYCHIATRY & NEUROSCIENCE*, *48*(3), E232–E239. https://doi.org/doi:10.1503/jpn.220208

Cordes, J. S., Mathiak, K. A., Dyck, M., Alawi, E. M., Gaber, T. J., Zepf, F. D., Klasen, M., Zvyagintsev, M., Gur, R. C., & Mathiak, K. (2015). Cognitive and neural strategies during control of the anterior cingulate cortex by fMRI neurofeedback in patients with schizophrenia. *FRONTIERS IN BEHAVIORAL NEUROSCIENCE*, *9*. https://doi.org/doi:10.3389/fnbeh.2015.00169

Cortese, A., Amano, K., Koizumi, A., Lau, H., & Kawato, M. (2017). Decoded fMRI neurofeedback can induce

bidirectional confidence changes within single participants. *Neuroimage*, *149*, 323–337. https://doi.org/doi:10.1016/j.neuroimage.2017.01.069

Debettencourt, M. T., Cohen, J. D., Lee, R. F., Norman, K. A., & Turk-Browne, N. B. (2015). Closed-loop training of attention with real-time brain imaging. *NATURE NEUROSCIENCE*, *18*(3), 165–470. https://doi.org/doi:10.1038/nn.3940

Debettencourt, M. T., Turk-Browne, N. B., & Norman, K. A. (2019). Neurofeedback helps to reveal a relationship between context reinstatement and memory retrieval. *Neuroimage*, *200*, 292–301. https://doi.org/doi:10.1016/j.neuroimage.2019.06.001

deCharms, R. C., Christoff, K., Glover, G. H., Pauly, J. M., Whitfield, S., & Gabrieli, J. D. (2004). Learned regulation of spatially localized brain activation using real-time fMRI. *Neuroimage*, *21*(1), 436–443. https://doi.org/10.1016/j.neuroimage.2003.08.041

deCharms, R. C., Maeda, F., Glover, G. H., Ludlow, D., Pauly, J. M., Soneji, D., Gabrieli, J. D., & Mackey, S. C. (2005). Control over brain activation and pain learned by using real-time functional MRI. *Proc Natl Acad Sci U S A*, *102*(51), 18626–18631. https://doi.org/10.1073/pnas.0505210102

Dewiputri, W. I., Schweizer, R., & Auer, T. (2021). Brain Networks Underlying Strategy Execution and Feedback Processing in an Efficient Functional Magnetic Resonance Imaging Neurofeedback Training Performed in a Parallel or a Serial Paradigm. *FRONTIERS IN HUMAN NEUROSCIENCE*, *15*. https://doi.org/doi:10.3389/fnhum.2021.645048

Direito, B., Lima, J., Simoes, M., Sayal, A., Sousa, T., Luhrs, M., Ferreira, C., & Castelo-Branco, M. (2019). Targeting dynamic facial processing mechanisms in superior temporal sulcus using a novel fMRI neurofeedback target. *NEUROSCIENCE*, *406*, 97–108. https://doi.org/doi:10.1016/j.neuroscience.2019.02.024

Direito, B., Mouga, S., Sayal, A., Simoes, M., Quental, H., Bernardino, I., Playle, R., McNamara, R., Linden, D. E. J., Oliveira, G., & Branco, M. C. (2021). Training the social brain: Clinical and neural effects of an 8-week real-time functional magnetic resonance imaging neurofeedback Phase IIa Clinical Trial in Autism. *AUTISM*, *25*(6), 1746–1760. https://doi.org/doi:10.1177/13623613211002052

Direito, B., Ramos, M., Pereira, J., Sayal, A., Sousa, T., & Castelo-Branco, M. (2021). Directly Exploring the Neural Correlates of Feedback-Related Reward Saliency and Valence During Real-Time fMRI-Based Neurofeedback. *FRONTIERS IN HUMAN NEUROSCIENCE*, *14*. https://doi.org/doi:10.3389/fnhum.2020.578119

Dyck, M. S., Mathiak, K. A., Bergert, S., Sarkheil, P., Koush, Y., Alawi, E. M., Zvyagintsev, M., Gaebler, A. J., Shergill, S. S., & Mathiak, K. (2016). Targeting Treatment-Resistant Auditory Verbal Hallucinations in Schizophrenia with fMRI-Based Neurofeedback - Exploring Different Cases of Schizophrenia. *FRONTIERS IN PSYCHIATRY*, *7*. https://doi.org/doi:10.3389/fpsyt.2016.00037

Ekanayake, J., Ridgway, G. R., Winston, J. S., Feredoes, E., Razi, A., Koush, Y., Scharnowski, F., Weiskopf, N., & Rees, G. (2019). Volitional modulation of higher-order visual cortex alters human perception. *Neuroimage*, *188*, 291–301. https://doi.org/doi:10.1016/j.neuroimage.2018.11.054

Emmert, K., Breimhorst, M., Bauermann, T., Birklein, F., Van de Ville, D., & Haller, S. (2014). Comparison of anterior cingulate vs. insular cortex as targets for real-time fMRI regulation during pain stimulation. *FRONTIERS IN BEHAVIORAL NEUROSCIENCE*, *8*. https://doi.org/doi:10.3389/fnbeh.2014.00350

Emmert, K., Kopel, R., Koush, Y., Maire, R., Senn, P., Van De Ville, D., & Haller, S. (2017). Continuous vs. intermittent neurofeedback to regulate auditory cortex activity of tinnitus patients using real-time fMRI - A pilot study. *NEUROIMAGE-CLINICAL*, *14*, 97–104. https://doi.org/doi:10.1016/j.nicl.2016.12.023

Fede, S. J., Kisner, M. A., Dean, S. F., Kerich, M., Roopchansingh, V., Diazgranados, N., & Momenan, R. (2023). Selecting an optimal real-time fMRI neurofeedback method for alcohol craving control training. *PSYCHOPHYSIOLOGY*, *60*(11). https://doi.org/doi:10.1111/psyp.14367

Frank, S., Lee, S., Preissl, H., Schultes, B., Birbaumer, N., & Veit, R. (2012). The obese brain athlete: self-regulation of the anterior insula in adiposity. *PLoS One*, *7*(8), e42570. https://doi.org/10.1371/journal.pone.0042570

Gao, H., Zhang, H., Wang, L., Zhang, C., Feng, Z., Li, Z., Tong, L., Yan, B., & Hu, G. (2023). Altered amygdala functional connectivity after real-time functional MRI emotion self-regulation training. *NEUROREPORT*, *34*(11), 537–545. https://doi.org/doi:10.1097/WNR.0000000000001921

Garrison, J. R., Saviola, F., Morgenroth, E., Barker, H., Luhrs, M., Simons, J. S., Fernyhough, C., & Allen, P. (2021). Modulating medial prefrontal cortex activity using real-time fMRI neurofeedback: Effects on reality monitoring performance and associated functional connectivity. *Neuroimage*, *245*. https://doi.org/doi:10.1016/j.neuroimage.2021.118640

Garrison, K. A., Scheinost, D., Worhunsky, P. D., Elwafi, H. M., Thornhill, T. A., Thompson, E., Saron, C., Desbordes, G., Kober, H., Hampson, M., Gray, J. R., Constable, R. T., Papademetris, X., & Brewer, J. A. (2013). Real-time fMRI links subjective experience with brain activity during focused attention. *Neuroimage*, *81*, 110–118. https://doi.org/doi:10.1016/j.neuroimage.2013.05.030

Gerin, M. I., Fichtenholtz, H., Roy, A., Walsh, C. J., Krystal, J. H., Southwick, S., & Hampson, M. (2016). Real-Time fMRI Neurofeedback with War Veterans with Chronic PTSD: A Feasibility Study. *FRONTIERS IN PSYCHIATRY*, *7*. https://doi.org/doi:10.3389/fpsyt.2016.00111

Greer, S. M., Trujillo, A. J., Glover, G. H., & Knutson, B. (2014). Control of nucleus accumbens activity with neurofeedback. *Neuroimage*, *96*, 237–244. https://doi.org/doi:10.1016/j.neuroimage.2014.03.073

Groene, M., Dyck, M., Koush, Y., Bergert, S., Mathiak, K. A., Alawi, E. M., Elliott, M., & Mathiak, K. (2015). Upregulation of the Rostral Anterior Cingulate Cortex can Alter the Perception of Emotions: fMRI-Based Neurofeedback at 3 and 7 T. *BRAIN TOPOGRAPHY*, *28*(2), 197–207. https://doi.org/doi:10.1007/s10548-014-0384-4

Guan, M., Ma, L., Li, L., Yan, B., Zhao, L., Tong, L., Dou, S., Xia, L., Wang, M., & Shi, D. (2015). Self-Regulation of Brain Activity in Patients with Postherpetic Neuralgia: A Double-Blind Randomized Study Using Real-Time fMRI Neurofeedback. *PLoS One*, *10*(4). https://doi.org/doi:10.1371/journal.pone.0123675

Guler, S., Cohen, A. L., Afacan, O., & Warfield, S. K. (2021). Matched neurofeedback during fMRI differentially activates reward-related circuits in active and sham groups. *JOURNAL OF NEUROIMAGING*, *31*(5), 947–955. https://doi.org/doi:10.1111/jon.12899

Habes, I., Rushton, S., Johnston, S. J., Sokunbi, M. O., Barawi, K., Brosnan, M., Daly, T., Ihssen, N., & Linden, D. E. J. (2016). fMRI neurofeedback of higher visual areas and perceptual biases. *NEUROPSYCHOLOGIA*, *85*, 208–215. https://doi.org/doi:10.1016/j.neuropsychologia.2016.03.031

Haller, S., Birbaumer, N., & Veit, R. (2010). Real-time fMRI feedback training may improve chronic tinnitus. *EUROPEAN RADIOLOGY*, *20*(3), 696–703. https://doi.org/doi:10.1007/s00330-009-1595-z

Hamilton, J. P., Glover, G. H., Bagarinao, E., Chang, C., Mackey, S., Sacchet, M. D., & Gotlib, I. H. (2016). Effects of salience-network-node neurofeedback training on affective biases in major depressive disorder. *PSYCHIATRY RESEARCH-NEUROIMAGING*, *249*, 91–96. https://doi.org/doi:10.1016/j.pscychresns.2016.01.016

Hamilton, J. P., Glover, G. H., Hsu, J.-J., Johnson, R. F., & Gotlib, I. H. (2011). Modulation of Subgenual Anterior Cingulate Cortex Activity With Real-Time Neurofeedback. *HUMAN BRAIN MAPPING*, *32*(1), 22–31. https://doi.org/doi:10.1002/hbm.20997

Hampson, M., Scheinost, D., Qiu, M., Bhawnani, J., Lacadie, C. M., Leckman, J. F., Constable, R. T., & Papademetris, X. (2011). Biofeedback of real-time functional magnetic resonance imaging data from the supplementary motor area reduces functional connectivity to subcortical regions. *Brain Connectivity*, *1*(1), 91–98. https://doi.org/doi:10.1089/brain.2011.0002

Hampson, M., Stoica, T., Saksa, J., Scheinost, D., Qiu, M., Bhawnani, J., Pittenger, C., Papademetris, X., & Constable, T. (2012). Real-time fMRI Biofeedback Targeting the Orbitofrontal Cortex for Contamination Anxiety. *JOVE-JOURNAL OF VISUALIZED EXPERIMENTS*, *59*. https://doi.org/doi:10.3791/3535

Harmelech, T., Friedman, D., & Malach, R. (2015). Differential Magnetic Resonance Neurofeedback Modulations across Extrinsic (Visual) and Intrinsic (Default-Mode) Nodes of the Human Cortex. *JOURNAL OF NEUROSCIENCE*, *35*(6), 2588–2595. https://doi.org/doi:10.1523/JNEUROSCI.3098-14.2015

Harmelech, T., Preminger, S., Wertman, E., & Malach, R. (2013). The Day-After Effect: Long Term, Hebbian-Like Restructuring of Resting-State fMRI Patterns Induced by a Single Epoch of Cortical Activation. *JOURNAL OF NEUROSCIENCE*, *33*(22), 9488–9497. https://doi.org/doi:10.1523/JNEUROSCI.5911-12.2013

Hartwell, K. J., Hanlon, C. A., Li, X., Borckardt, J. J., Canterberry, M., Prisciandaro, J. J., Moran-Santa Maria, M. M., LeMatty, T., George, M. S., & Brady, K. T. (2016). Individualized real-time fMRI neurofeedback to attenuate craving in nicotine-dependent smokers. *JOURNAL OF PSYCHIATRY & NEUROSCIENCE*, *41*(1), 48–55. https://doi.org/doi:10.1503/jpn.140200

Haugg, A., Frei, N., Menghini, M., Stutz, F., Steinegger, S., Rothlisberger, M., & Brem, S. (2023). Self-regulation of visual word form area activation with real-time fMRI neurofeedback. *SCIENTIFIC REPORTS*, *13*(1). https://doi.org/doi:10.1038/s41598-023-35932-9

Hellrung, L., Dietrich, A., Hollmann, M., Pleger, B., Kalberlah, C., Roggenhofer, E., Villringer, A., & Horstmann, A. (2018). Intermittent compared to continuous real-time fMRI neurofeedback boosts control over amygdala activation. *Neuroimage*, *166*, 198–208. https://doi.org/doi:10.1016/j.neuroimage.2017.10.031

Herwig, U., Lutz, J., Scherpiet, S., Scheerer, H., Kohlberg, J., Opialla, S., Preuss, A., Steiger, V. R., Sulzer, J., Weidt, S., Staempfli, P., Rufer, M., Seifritz, E., Jancke, L., & Bruehl, A. B. (2019). Training emotion regulation through real-time fMRI neurofeedback of amygdala activity. *Neuroimage*, *184*, 687–696. https://doi.org/doi:10.1016/j.neuroimage.2018.09.068

Hohenfeld, C., Nellessen, N., Dogan, I., Kuhn, H., Mueller, C., Papa, F., Ketteler, S., Goebel, R., Heinecke, A., Shah, N. J., Schulz, J. B., Reske, M., & Reetz, K. (2017). Cognitive Improvement and Brain Changes after Real-Time Functional MRI Neurofeedback Training in Healthy Elderly and Prodromal Alzheimer's Disease. *FRONTIERS IN NEUROLOGY*, *8*. https://doi.org/doi:10.3389/fneur.2017.00384

Horovitz, S. G., Berman, B. D., & Hallett, M. (2010). Real time BOLD functional MRI neuro-feedback affects functional connectivity. *Annu Int Conf IEEE Eng Med Biol Soc*, *2010*, 4270–4273. https://doi.org/doi:10.1109/iembs.2010.5627170

Hui, M., Zhang, H., Ge, R., Yao, L., & Long, Z. (2014). Modulation of functional network with real-time fMRI feedback training of right premotor cortex activity. *NEUROPSYCHOLOGIA*, *62*, 111–123. https://doi.org/doi:10.1016/j.neuropsychologia.2014.07.012

Ihssen, N., Sokunbi, M. O., Lawrence, A. D., Lawrence, N. S., & Linden, D. E. J. (2017). Neurofeedback of visual food cue reactivity: a potential avenue to alter incentive sensitization and craving. *BRAIN IMAGING AND BEHAVIOR*, *11*(3), 915–924. https://doi.org/doi:10.1007/s11682-016-9558-x

Jaeckle, T., Williams, S. C. R., Barker, G. J., Basilio, R., Carr, E., Goldsmith, K., Colasanti, A., Giampietro, V., Cleare, A., Young, A. H., Moll, J., & Zahn, R. (2023). Self-blame in major depression: a randomised pilot trial comparing fMRI neurofeedback with self-guided psychological strategies. *PSYCHOLOGICAL MEDICINE*, *53*(7), 2831–2841. https://doi.org/doi:10.1017/S0033291721004797

Johnson, K. A., Hartwell, K., LeMatty, T., Borckardt, J., Morgan, P. S., Govindarajan, K., Brady, K., & George, M. S. (2012). Intermittent "Real-time" fMRI Feedback Is Superior to Continuous Presentation for a Motor Imagery Task: A Pilot Study. *JOURNAL OF NEUROIMAGING*, *22*(1), 58–66. https://doi.org/doi:10.1111/j.1552-6569.2010.00529.x

Johnston, S. J., Boehm, S. G., Healy, D., Goebel, R., & Linden, D. E. J. (2010). Neurofeedback: A promising tool for the self-regulation of emotion networks. *Neuroimage*, *49*(1), 1066–1072. https://doi.org/doi:10.1016/j.neuroimage.2009.07.056

Johnston, S., Linden, D. E. J., Healy, D., Goebel, R., Habes, I., & Boehm, S. G. (2011). Upregulation of emotion areas through neurofeedback with a focus on positive mood. *COGNITIVE AFFECTIVE & BEHAVIORAL NEUROSCIENCE*, *11*(1), 44–51. https://doi.org/doi:10.3758/s13415-010-0010-1

Kaas, A., Goebel, R., Valente, G., & Sorger, B. (2019). Topographic Somatosensory Imagery for Real-Time fMRI Brain-Computer Interfacing. *FRONTIERS IN HUMAN NEUROSCIENCE*, *13*. https://doi.org/doi:10.3389/fnhum.2019.00427

Kadosh, K. C., Luo, Q., de Burca, C., Sokunbi, M. O., Feng, J., Linden, D. E. J., & Lau, J. Y. F. (2016). Using real-time fMRI to influence effective connectivity in the developing emotion regulation network. *Neuroimage*, *125*, 616–626. https://doi.org/doi:10.1016/j.neuroimage.2015.09.070

Kanel, D., Al-Wasity, S., Stefanov, K., & Pollick, F. E. (2019). Empathy to emotional voices and the use of real-time fMRI to enhance activation of the anterior insula. *Neuroimage*, *198*, 53–62. https://doi.org/doi:10.1016/j.neuroimage.2019.05.021

Karch, S., Keeser, D., Huemmer, S., Paolini, M., Kirsch, V., Karali, T., Kupka, M., Rauchmann, B.-S., Chrobok, A., Blautzik, J., Koller, G., Ertl-Wagner, B., & Pogarell, O. (2015). Modulation of Craving Related Brain Responses Using Real-Time fMRI in Patients with Alcohol Use Disorder. *PLoS One*, *10*(7). https://doi.org/doi:10.1371/journal.pone.0133034

Karch, S., Krause, D., Lehnert, K., Konrad, J., Haller, D., Rauchmann, B.-S., Maywald, M., Engelbregt, H., Adorjan, K., Koller, G., Reidler, P., Karali, T., Tschentscher, N., Ertl-Wagner, B., Pogarell, O., Paolini, M., & Keeser, D. (2022). Functional and clinical outcomes of FMRI-based neurofeedback training in patients with alcohol dependence: a pilot study. *EUROPEAN ARCHIVES OF PSYCHIATRY AND CLINICAL NEUROSCIENCE*, *272*(4), 557–569. https://doi.org/doi:10.1007/s00406-021-01336-x

Karch, S., Paolini, M., Gschwendtner, S., Jeanty, H., Reckenfelderbäumer, A., Yaseen, O., Maywald, M., Fuchs, C., Rauchmann, B. S., Chrobok, A., Rabenstein, A., Ertl-Wagner, B., Pogarell, O., Keeser, D., & Rüther, T. (2019). Real-Time fMRI Neurofeedback in Patients With Tobacco Use Disorder During Smoking Cessation: Functional Differences and Implications of the First Training Session in Regard to Future Abstinence or Relapse. *Front Hum Neurosci*, *13*, 65. https://doi.org/doi:10.3389/fnhum.2019.00065

Keller, M., Zweerings, J., Klasen, M., Zvyagintsev, M., Iglesias, J., Quinones, R. M., & Mathiak, K. (2021). fMRI Neurofeedback-Enhanced Cognitive Reappraisal Training in Depression: A Double-Blind Comparison of Left and Right vlPFC Regulation. *FRONTIERS IN PSYCHIATRY*, *12*. https://doi.org/doi:10.3389/fpsyt.2021.715898

Kim, D. Y., Yoo, S. S., Tegethoff, M., Meinlschmidt, G., & Lee, J. H. (2015). The Inclusion of Functional Connectivity Information into fMRI-based Neurofeedback Improves Its Efficacy in the Reduction of Cigarette Cravings. *Journal of Cognitive Neuroscience*, *27*(8), 1552–1572. https://doi.org/Journal of Cognitive Neuroscience

Kim, H. C., Tegethoff, M., Meinlschmidt, G., Stalujanis, E., Belardi, A., Jo, S., Lee, J., Kim, D. Y., Yoo, S. S., & Lee, J. H. (2019). Mediation analysis of triple networks revealed functional feature of mindfulness from real-time fMRI neurofeedback. *NeuroImage*, *195*(March), 409–432. https://doi.org/10.1016/j.neuroimage.2019.03.066

Kirlic, N., Cohen, Z. P., Tsuchiyagaito, A., Misaki, M., McDermott, T. J., Aupperle, R. L., Stewart, J. L., Singh, M. K., Paulus, M. P., & Bodurka, J. (2022). Self-regulation of the posterior cingulate cortex with real-time fMRI neurofeedback augmented mindfulness training in healthy adolescents: A nonrandomized feasibility study. *COGNITIVE AFFECTIVE & BEHAVIORAL NEUROSCIENCE*, *22*(4), 849–867. https://doi.org/doi:10.3758/s13415-022-00991-4

Kirsch, M., Gruber, I., Ruf, M., Kiefer, F., & Kirsch, P. (2016). Real-time functional magnetic resonance imaging neurofeedback can reduce striatal cue-reactivity to alcohol stimuli. *Addiction Biology*, *21*(4), 982–992. https://doi.org/10.1111/adb.12278

Kirschner, M., Sladky, R., Haugg, A., Stampfli, P., Jehli, E., Hodel, M., Engeli, E., Hosli, S., Baumgartner, M. R., Sulzer, J., Huys, Q. J. M., Seifritz, E., Quednow, B. B., Scharnowski, F., & Herdener, M. (2018). Self-regulation of the dopaminergic reward circuit in cocaine users with mental imagery and neurofeedback. *EBIOMEDICINE*, *37*, 489–498. https://doi.org/doi:10.1016/j.ebiom.2018.10.052

Klöbl, M., Michenthaler, P., Godbersen, G. M., Robinson, S., Hahn, A., & Lanzenberger, R. (2020). Reinforcement and Punishment Shape the Learning Dynamics in fMRI Neurofeedback. *Front Hum Neurosci*, *14*, 304. https://doi.org/doi:10.3389/fnhum.2020.00304

Kober, S. E., Groessinger, D., & Wood, G. (2019). Effects of Motor Imagery and Visual Neurofeedback on Activation in the Swallowing Network: A Real-Time fMRI Study. *DYSPHAGIA*, *34*(6), 879–895. https://doi.org/doi:10.1007/s00455-019-09985-w

Kohl, S. H., Veit, R., Spetter, M. S., Guenther, A., Rina, A., Luehrs, M., Birbaumer, N., Preissl, H., & Hallschmid, M. (2019). Real-time fMRI neurofeedback training to improve eating behavior by self-regulation of the dorsolateral prefrontal cortex: A randomized controlled trial in overweight and obese subjects. *Neuroimage*, *191*, 596–609. https://doi.org/doi:10.1016/j.neuroimage.2019.02.033

Koizumi, A., Amano, K., Cortese, A., Shibata, K., Yoshida, W., Seymour, B., Kawato, M., & Lau, H. (2017). Fear reduction without fear through reinforcement of neural activity that bypasses conscious exposure. *NATURE HUMAN BEHAVIOUR*, *1*(1). https://doi.org/doi:10.1038/s41562-016-0006

Koush, Y., Meskaldji, D.-E., Pichon, S., Rey, G., Rieger, S. W., Linden, D. E. J., Van de Ville, D., Vuilleumier, P., & Scharnowski, F. (2017). Learning Control Over Emotion Networks Through Connectivity-Based Neurofeedback. *CEREBRAL CORTEX*, *27*(2), 1193–1202. https://doi.org/doi:10.1093/cercor/bhv311

Koush, Y., Rosa, M. J., Robineau, F., Heinen, K., Rieger, S. W., Weiskopf, N., Vuilleumier, P., Van De Ville, D., & Scharnowski, F. (2013). Connectivity-based neurofeedback: Dynamic causal modeling for real-time fMRI. *Neuroimage*, *81*, 422–430. https://doi.org/doi:10.1016/j.neuroimage.2013.05.010

Krause, F., Benjamins, C., Luhrs, M., Eck, J., Noirhomme, Q., Rosenke, M., Brunheim, S., Sorger, B., & Goebel, R. (2017). Real-time fMRI-based self-regulation of brain activation across different visual feedback presentations. *BRAIN-COMPUTER INTERFACES*, *4*(1), 87–101. https://doi.org/doi:10.1080/2326263X.2017.1307096

Krause, F., Kogias, N., Krentz, M., Luhrs, M., Goebel, R., & Hermans, E. J. (2021). Self-regulation of stress-related large-scale brain network balance using real-time fMRI neurofeedback. *Neuroimage*, *243*. https://doi.org/doi:10.1016/j.neuroimage.2021.118527

Lam, S.-L., Criaud, M., Lukito, S., Westwood, S. J., Agbedjro, D., Kowalczyk, O. S., Curran, S., Barret, N., Abbott, C., Liang, H., Simonoff, E., Barker, G. J., Giampietro, V., & Rubia, K. (2022). Double-Blind, Sham-Controlled Randomized Trial Testing the Efficacy of fMRI Neurofeedback on Clinical and Cognitive Measures in Children With ADHD. *The American Journal of Psychiatry*, *179*(12), 947–958. https://doi.org/doi:10.1176/appi.ajp.21100999

Lawrence, E. J., Su, L., Barker, G. J., Medford, N., Dalton, J., Williams, S. C. R., Birbaumer, N., Veit, R., Ranganatha, S., Bodurka, J., Brammer, M., Giampietro, V., & David, A. S. (2014). Self-regulation of the anterior insula: Reinforcement learning using real-time fMRI neurofeedback. *Neuroimage*, *88*, 113–124. https://doi.org/doi:10.1016/j.neuroimage.2013.10.069

Lee, D., Jang, C., & Park, H.-J. (2019). Neurofeedback learning for mental practice rather than repetitive practice improves neural pattern consistency and functional network efficiency in the subsequent mental motor execution. *Neuroimage*, *188*, 680–693. https://doi.org/doi:10.1016/j.neuroimage.2018.12.055

Li, X., Hartwell, K. J., Borckardt, J., Prisciandaro, J. J., Saladin, M. E., Morgan, P. S., Johnson, K. A., Lematty, T., Brady, K. T., & George, M. S. (2013). Volitional reduction of anterior cingulate cortex activity produces decreased cue craving in smoking cessation: A preliminary real-time fMRI study. *Addiction Biology*, *18*(4), 739–748. https://doi.org/10.1111/j.1369-1600.2012.00449.x

Li, X., Li, Z., Zou, Z., Wu, X., Gao, H., Wang, C., Zhou, J., Qi, F., Zhang, M., He, J., Qi, X., Yan, F., Dou, S., Zhang, H., Tong, L., & Li, Y. (2022). Real-Time fMRI Neurofeedback Training Changes Brain Degree Centrality and Improves Sleep in Chronic Insomnia Disorder: A Resting-State fMRI Study. *FRONTIERS IN MOLECULAR NEUROSCIENCE*, *15*. https://doi.org/doi:10.3389/fnmol.2022.825286

Li, Z., Liu, J., Chen, B., Wu, X., Zou, Z., Gao, H., Wang, C., Zhou, J., Qi, F., Zhang, M., He, J., Qi, X., Yan, F., Dou, S., Tong, L., Zhang, H., Han, X., & Li, Y. (2022). Improved Regional Homogeneity in Chronic Insomnia Disorder After Amygdala-Based Real-Time fMRI Neurofeedback Training. *FRONTIERS IN PSYCHIATRY*, *13*. https://doi.org/doi:10.3389/fpsyt.2022.863056

Li, Z., Tong, L., Guan, M., He, W., Wang, L., Bu, H., Shi, D., & Yan, B. (2016). Altered Resting-State Amygdala Functional Connectivity after Real-Time fMRI Emotion Self-Regulation Training. *BIOMED RESEARCH INTERNATIONAL*, *2016*. https://doi.org/doi:10.1155/2016/2719895

Li, Z., Tong, L., Wang, L., Li, Y., He, W., Guan, M., & Yan, B. (2016). Self-regulating positive emotion networks by

feedback of multiple emotional brain states using real-time fMRI. *EXPERIMENTAL BRAIN RESEARCH*, *234*(12), 3575–3586. https://doi.org/doi:10.1007/s00221-016-4744-z

Li, Z., Zhang, C., Huang, J., Wang, Y., Yan, C., Li, K., Zeng, Y., Jin, Z., Cheung, E. F. C., Su, L., & Chan, R. C. K. (2018). Improving Motivation Through Real-Time fMRI-Based Self-Regulation of the Nucleus Accumbens. *NEUROPSYCHOLOGY*, *32*(6), 764–776. https://doi.org/doi:10.1037/neu0000425

Liew, S.-L., Rana, M., Cornelsen, S., de Barros Filho, M. F., Birbaumer, N., Sitaram, R., Cohen, L. G., & Soekadar, S. R. (2016). Improving Motor Corticothalamic Communication After Stroke Using Real-Time fMRI Connectivity-Based Neurofeedback. *NEUROREHABILITATION AND NEURAL REPAIR*, *30*(7), 671–675. https://doi.org/doi:10.1177/1545968315619699

Linden, D. E. J., Habes, I., Johnston, S. J., Linden, S., Tatineni, R., Subramanian, L., Sorger, B., Healy, D., & Goebel, R. (2012). Real-Time Self-Regulation of Emotion Networks in Patients with Depression. *PLoS One*, *7*(6). https://doi.org/doi:10.1371/journal.pone.0038115

Lisk, S., Kadosh, K. C., Zich, C., Haller, S. P. W., & Lau, J. Y. F. (2020). Training negative connectivity patterns between the dorsolateral prefrontal cortex and amygdala through fMRI-based neurofeedback to target adolescent socially-avoidant behaviour. *BEHAVIOUR RESEARCH AND THERAPY*, *135*. https://doi.org/doi:10.1016/j.brat.2020.103760

Liu, N., Yao, L., & Zhao, X. (2020). Evaluating the amygdala network induced by neurofeedback training for emotion regulation using hierarchical clustering. *BRAIN RESEARCH*, *1740*. https://doi.org/doi:10.1016/j.brainres.2020.146853

Liu, N., Yu, X., Yao, L., & Zhao, X. (2018). Mapping the Cortical Network Arising From Up-Regulated Amygdaloidal Activation Using λ-Louvain Algorithm. *IEEE TRANSACTIONS ON NEURAL SYSTEMS AND REHABILITATION ENGINEERING*, *26*(6), 1169–1177. https://doi.org/doi:10.1109/TNSRE.2018.2838075

MacDuffie, K. E., MacInnes, J., Dickerson, K. C., Eddington, K. M., Strauman, T. J., & Adcock, R. A. (2018). Single session real-time fMRI neurofeedback has a lasting impact on cognitive behavioral therapy strategies. *NEUROIMAGE-CLINICAL*, *19*, 868–875. https://doi.org/doi:10.1016/j.nicl.2018.06.009

MacInnes, J. J., Dickerson, K. C., Chen, N. K., & Adcock, R. A. (2016). Cognitive Neurostimulation: Learning to Volitionally Sustain Ventral Tegmental Area Activation. *Neuron*, *89*(6), 1331–1342. https://doi.org/doi:10.1016/j.neuron.2016.02.002

Madkhali, Y., Al-Wasity, S., Aldehmi, N., & Pollick, F. (2022). Using Real-Time fMRI Neurofeedback to Modulate M1-Cerebellum Connectivity. *COMPUTATIONAL INTELLIGENCE AND NEUROSCIENCE*, *2022*. https://doi.org/doi:10.1155/2022/8744982

Margolles, P., Elosegi, P., Mei, N., & Soto, D. (2023). Unconscious manipulation of conceptual representations with decoded neurofeedback impacts search behaviour. *The Journal of Neuroscience : The Official Journal of the Society for Neuroscience*. https://doi.org/doi:10.1523/JNEUROSCI.1235-23.2023

Marins, T. F., Rodrigues, E. C., Engel, A., Hoefle, S., Basilio, R., Lent, R., Moll, J., & Tovar-Moll, F. (2015). Enhancing Motor Network Activity Using Real-Time Functional MRI Neurofeedback of Left Premotor Cortex. *FRONTIERS IN BEHAVIORAL NEUROSCIENCE*, *9*. https://doi.org/doi:10.3389/fnbeh.2015.00341

Marins, T., Rodrigues, E. C., Bortolini, T., Melo, B., Moll, J., & Tovar-Moll, F. (2019). Structural and functional connectivity changes in response to short-term neurofeedback training with motor imagery. *Neuroimage*, *194*, 283–290. https://doi.org/doi:10.1016/j.neuroimage.2019.03.027

Marxen, M., Jacob, M. J., Mueller, D. K., Posse, S., Ackley, E., Hellrung, L., Riedel, P., Bender, S., Epple, R., & Smolka, M. N. (2016). Amygdala Regulation Following fMRI-Neurofeedback without Instructed Strategies. *FRONTIERS IN HUMAN NEUROSCIENCE*, *10*. https://doi.org/doi:10.3389/fnhum.2016.00183

Mathiak, K. A., Alawi, E. M., Koush, Y., Dyck, M., Cordes, J. S., Gaber, T. J., Zepf, F. D., Palomero-Gallagher, N., Sarkheil, P., Bergert, S., Zvyagintsev, M., & Mathiak, K. (2015). Social reward improves the voluntary control over localized brain activity in fMRI-based neurofeedback training. *FRONTIERS IN BEHAVIORAL NEUROSCIENCE*, *9*. https://doi.org/doi:10.3389/fnbeh.2015.00136

Mayeli, A., Misaki, M., Zotev, V., Tsuchiyagaito, A., Al Zoubi, O., Phillips, R., Smith, J., Stewart, J. L., Refai, H., Paulus, M. P., & Bodurka, J. (2020). Self-regulation of ventromedial prefrontal cortex activation using real-time fMRI neurofeedback-Influence of default mode network. *HUMAN BRAIN MAPPING*, *41*(2), 342–352. https://doi.org/doi:10.1002/hbm.24805

Maywald, M., Paolini, M., Rauchmann, B. S., Gerz, C., Heppe, J. L., Wolf, A., Lerchenberger, L., Tominschek, I., Stöcklein, S., Reidler, P., Tschentscher, N., Ertl-Wagner, B., Pogarell, O., Keeser, D., & Karch, S. (2022). Individual- and Connectivity-Based Real-Time fMRI Neurofeedback to Modulate Emotion-Related Brain Responses in Patients with Depression: A Pilot Study. *Brain Sci*, *12*(12). https://doi.org/doi:10.3390/brainsci12121714

McCaig, R. G., Dixon, M., Keramatian, K., Liu, I., & Christoff, K. (2011). Improved modulation of rostrolateral prefrontal cortex using real-time fMRI training and meta-cognitive awareness. *Neuroimage*, *55*(3), 1298–1305. https://doi.org/10.1016/j.neuroimage.2010.12.016

Megumi, F., Yamashita, A., Kawato, M., & Imamizu, H. (2015). Functional MRI neurofeedback training on connectivity between two regions induces long-lasting changes in intrinsic functional network. *FRONTIERS IN HUMAN NEUROSCIENCE*, *9*. https://doi.org/doi:10.3389/fnhum.2015.00160

Mehler, D. M. A., Sokunbi, M. O., Habes, I., Barawi, K., Subramanian, L., Range, M., Evans, J., Hoods, K., Luhrs, M., Keedwell, P., Goebel, R., & Linden, D. E. J. (2018). Targeting the affective brain-a randomized controlled trial of real-time fMRI neurofeedback in patients with depression. *NEUROPSYCHOPHARMACOLOGY*, *43*(13), 2578–2585. https://doi.org/doi:10.1038/s41386-018-0126-5

Mehler, D. M. A., Williams, A. N., Krause, F., Luehrs, M., Wise, R. G., Turner, D. L., Linden, D. E. J., & Whittaker, J. R. (2019). The BOLD response in primary motor cortex and supplementary motor area during kinesthetic motor imagery based graded fMRI neurofeedback. *Neuroimage*, *184*, 36–44. https://doi.org/doi:10.1016/j.neuroimage.2018.09.007

Mehler, D. M. A., Williams, A. N., Whittaker, J. R., Krause, F., Luhrs, M., Kunas, S., Wise, R. G., Shetty, H. G. M., Turner, D. L., & Linden, D. E. J. (2020). Graded fMRI Neurofeedback Training of Motor Imagery in Middle Cerebral Artery Stroke Patients: A Preregistered Proof-of-Concept Study. *FRONTIERS IN HUMAN NEUROSCIENCE*, *14*. https://doi.org/doi:10.3389/fnhum.2020.00226

Mel'nikov, M. Y., Bezmaternykh, D. D., Savelov, A. A., Petrovskiy, E. D., Kozlova, L. I., Natarova, K. A., Larina, T. D., Andamova, T. M., Zvyagintsev, M., Shtark, M. B., & Mathiak, K. (2023). Real-time fMRI neurofeedback compared to cognitive behavioral therapy in a pilot study for the treatment of mild and moderate depression. *EUROPEAN ARCHIVES OF PSYCHIATRY AND CLINICAL NEUROSCIENCE*, *273*(5), 1139–1149. https://doi.org/doi:10.1007/s00406-022-01462-0

Mennen, A. C., Nastase, S. A., Yeshurun, Y., Hasson, U., & Norman, K. A. (2022). Real-time neurofeedback to alter interpretations of a naturalistic narrative. *Neuroimage. Reports*, *2*(3). https://doi.org/doi:10.1016/j.ynirp.2022.100111

Mennen, A. C., Turk-Browne, N. B., Wallace, G., Seok, D., Jaganjac, A., Stock, J., DeBettencourt, M. T., Cohen, J. D., Norman, K. A., & Sheline, Y. I. (2021). Cloud-Based Functional Magnetic Resonance Imaging Neurofeedback to Reduce the Negative Attentional Bias in Depression: A Proof-of-Concept Study. *BIOLOGICAL PSYCHIATRY-COGNITIVE NEUROSCIENCE AND NEUROIMAGING*, *6*(4), 490–497. https://doi.org/doi:10.1016/j.bpsc.2020.10.006

Misaki, M., Phillips, R., Zotev, V., Wong, C. K., Wurfel, B. E., Krueger, F., Feldner, M., & Bodurka, J. (2018). Real-time fMRI amygdala neurofeedback positive emotional training normalized resting-state functional connectivity in combat veterans with and without PTSD: a connectome-wide investigation. *NeuroImage: Clinical*, *20*(August), 543–555. https://doi.org/10.1016/j.nicl.2018.08.025

Moll, J., Weingartner, J. H., Bado, P., Basilio, R., Sato, J. R., Melo, B. R., Bramati, I. E., de Oliveira-Souza, R., & Zahn, R. (2014). Voluntary Enhancement of Neural Signatures of Affiliative Emotion Using fMRI Neurofeedback. *PLoS One*, *9*(5). https://doi.org/doi:10.1371/journal.pone.0097343

Morgenroth, E., Saviola, F., Gilleen, J., Allen, B., Luhrs, M., Eysenck, M. W., & Allen, P. (2020). Using connectivity-

based real-time fMRI neurofeedback to modulate attentional and resting state networks in people with high trait anxiety. *NEUROIMAGE-CLINICAL*, *25*. https://doi.org/doi:10.1016/j.nicl.2020.102191

Neyedli, H. F., Sampaio-Baptista, C., Kirkman, M. A., Havard, D., Luhrs, M., Ramsden, K., Flitney, D. D., Clare, S., Goebel, R., & Johansen-Berg, H. (2018). Increasing Lateralized Motor Activity in Younger and Older Adults using Real-time fMRI during Executed Movements. *NEUROSCIENCE*, *378*, 165–174. https://doi.org/doi:10.1016/j.neuroscience.2017.02.010

Nicholson, A. A., Rabellino, D., Densmore, M., Frewen, P. A., Paret, C., Kluetsch, R., Schmahl, C., Theberge, J., Neufeld, R. W. J., McKinnon, M. C., Reiss, J., Jetly, R., & Lanius, R. A. (2017). The neurobiology of emotion regulation in posttraumatic stress disorder: Amygdala downregulation via real-time fMRI neurofeedback. *HUMAN BRAIN MAPPING*, *38*(1), 541–560. https://doi.org/doi:10.1002/hbm.23402

Nicholson, A. A., Rabellino, D., Densmore, M., Frewen, P. A., Steryl, D., Scharnowski, F., Theberge, J., Neufeld, R. W. J., Schmahl, C., Jetly, R., & Lanius, R. A. (2022). Differential mechanisms of posterior cingulate cortex downregulation and symptom decreases in posttraumatic stress disorder and healthy individuals using real-time fMRI neurofeedback. *BRAIN AND BEHAVIOR*, *12*(1). https://doi.org/doi:10.1002/brb3.2441

Oblak, E., Lewis-Peacock, J., & Sulzer, J. (2021). Differential neural plasticity of individual fingers revealed by fMRI neurofeedback. *JOURNAL OF NEUROPHYSIOLOGY*, *125*(5), 1720–1734. https://doi.org/doi:10.1152/jn.00509.2020

Okano, K., Bauer, C. C. C., Ghosh, S. S., Lee, Y. J., Melero, H., de los Angeles, C., Nestor, P. G., del Re, E. C., Northoff, G., Whitfield-Gabrieli, S., & Niznikiewicz, M. A. (2020). Real-time fMRI feedback impacts brain activation, results in auditory hallucinations reduction: Part 1: Superior temporal gyrus -Preliminary evidence. *PSYCHIATRY RESEARCH*, *286*. https://doi.org/doi:10.1016/j.psychres.2020.112862

Orlov, N. D., Giampietro, V., O'Daly, O., Lam, S. L., Barker, G. J., Rubia, K., McGuire, P., Shergill, S. S., & Allen, P. (2018). Real-time fMRI neurofeedback to down-regulate superior temporal gyrus activity in patients with schizophrenia and auditory hallucinations: a proof-of-concept study. *Transl Psychiatry*, *8*(1), 46. https://doi.org/doi:10.1038/s41398-017-0067-5

Pamplona, G. S. P., Heldner, J., Langner, R., Koush, Y., Michels, L., Ionta, S., Scharnowski, F., & Salmon, C. E. G. (2020). Network-based fMRI-neurofeedback training of sustained attention. *Neuroimage*, *221*. https://doi.org/doi:10.1016/j.neuroimage.2020.117194

Papoutsi, M., Magerkurth, J., Josephs, O., Pepes, S. E., Ibitoye, T., Reilmann, R., Hunt, N., Payne, E., Weiskopf, N., Langbehn, D., Rees, G., & Tabrizi, S. J. (2020). Activity or connectivity? A randomized controlled feasibility study evaluating neurofeedback training in Huntington's disease. *Brain Communications*, *2*(1), fcaa049–fcaa049. https://doi.org/doi:10.1093/braincomms/fcaa049

Papoutsi, M., Weiskopf, N., Langbehn, D., Reilmann, R., Rees, G., & Tabrizi, S. J. (2018). Stimulating neural plasticity with real-time fMRI neurofeedback in Huntington's disease: A proof of concept study. *HUMAN BRAIN MAPPING*, *39*(3), 1339–1353. https://doi.org/doi:10.1002/hbm.23921

Paret, C., Kluetsch, R., Ruf, M., Demirakca, T., Hoesterey, S., Ende, G., & Schmahl, C. (2014). Down-regulation of amygdala activation with real-time fMRI neurofeedback in a healthy female sample. *FRONTIERS IN BEHAVIORAL NEUROSCIENCE*, *8*. https://doi.org/doi:10.3389/fnbeh.2014.00299

Paret, C., Kluetsch, R., Zaehringer, J., Ruf, M., Demirakca, T., Bohus, M., Ende, G., & Schmahl, C. (2016). Alterations of amygdala-prefrontal connectivity with real-time fMRI neurofeedback in BPD patients. *SOCIAL COGNITIVE AND AFFECTIVE NEUROSCIENCE*, *11*(6), 952–960. https://doi.org/doi:10.1093/scan/nsw016

Paret, C., Zaehringer, J., Ruf, M., Gerchen, M. F., Mall, S., Hendler, T., Schmahl, C., & Ende, G. (2018). Monitoring and control of amygdala neurofeedback involves distributed information processing in the human brain. *HUMAN BRAIN MAPPING*, *39*(7), 3018–3031. https://doi.org/doi:10.1002/hbm.24057

Pereira, D. J., Sayal, A., Pereira, J., Morais, S., Macedo, A., Direito, B., & Castelo-Branco, M. (2023). Neurofeedback-dependent influence of the ventral striatum using a working memory paradigm targeting the dorsolateral prefrontal cortex. *FRONTIERS IN BEHAVIORAL NEUROSCIENCE*, *17*.

https://doi.org/doi:10.3389/fnbeh.2023.1014223

Pereira, J. A., Sepulveda, P., Rana, M., Montalba, C., Tejos, C., Torres, R., Sitaram, R., & Ruiz, S. (2019). Self-Regulation of the Fusiform Face Area in Autism Spectrum: A Feasibility Study With Real-Time fMRI Neurofeedback. *FRONTIERS IN HUMAN NEUROSCIENCE*, *13*. https://doi.org/doi:10.3389/fnhum.2019.00446

Pereira, J., Direito, B., Sayal, A., Ferreira, C., & Castelo-Branco, M. (2019). Self-Modulation of Premotor Cortex Interhemispheric Connectivity in a Real-Time Functional Magnetic Resonance Imaging Neurofeedback Study Using an Adaptive Approach. *BRAIN CONNECTIVITY*, *9*(9), 662–672. https://doi.org/doi:10.1089/brain.2019.0697

Quevedo, K., Liu, G., Teoh, J. Y., Ghosh, S., Zeffiro, T., Ahrweiler, N., Zhang, N., Wedan, R., Oh, S., Guercio, G., & Paret, C. (2019). Neurofeedback and neuroplasticity of visual self-processing in depressed and healthy adolescents: A preliminary study. *DEVELOPMENTAL COGNITIVE NEUROSCIENCE*, *40*. https://doi.org/doi:10.1016/j.dcn.2019.100707

Quevedo, K., Teoh, J. Y., Engstrom, M., Wedan, R., Santana-Gonzalez, C., Zewde, B., Porter, D., & Kadosh, K. C. (2020). Amygdala Circuitry During Neurofeedback Training and Symptoms' Change in Adolescents With Varying Depression. *FRONTIERS IN BEHAVIORAL NEUROSCIENCE*, *14*. https://doi.org/doi:10.3389/fnbeh.2020.00110

Ramot, M., Grossman, S., Friedman, D., & Malach, R. (2016). Covert neurofeedback without awareness shapes cortical network spontaneous connectivity. *PROCEEDINGS OF THE NATIONAL ACADEMY OF SCIENCES OF THE UNITED STATES OF AMERICA*, *113*(17), E2413–E2420. https://doi.org/doi:10.1073/pnas.1516857113

Ramot, M., Kimmich, S., Gonzalez-Castillo, J., Roopchansingh, V., Popal, H., White, E., Gotts, S. J., & Martin, A. (2017). Direct modulation of aberrant brain network connectivity through real-time NeuroFeedback. *ELIFE*, *6*. https://doi.org/doi:10.7554/eLife.28974

Rana, M., Ruiz, S., Sanchez Corzo, A., Muehleck, A., Eck, S., Salinas, C., Zamorano, F., Silva, C., Rea, M., Batra, A., Birbaumer, N., & Sitaram, R. (2020). Use of Real-Time Functional Magnetic Resonance Imaging-Based Neurofeedback to Downregulate Insular Cortex in Nicotine-Addicted Smokers. *JOVE-JOURNAL OF VISUALIZED EXPERIMENTS*, *160*. https://doi.org/doi:10.3791/59441

Rance, M., Ruttorf, M., Nees, F., Schad, L. R., & Flor, H. (2014a). Neurofeedback of the difference in activation of the anterior cingulate cortex and posterior insular cortex: two functionally connected areas in the processing of pain. *FRONTIERS IN BEHAVIORAL NEUROSCIENCE*, *8*. https://doi.org/doi:10.3389/fnbeh.2014.00357

Rance, M., Ruttorf, M., Nees, F., Schad, L. R., & Flor, H. (2014b). Real Time fMRI Feedback of the Anterior Cingulate and Posterior Insular Cortex in the Processing of Pain. *HUMAN BRAIN MAPPING*, *35*(12), 5784–5798. https://doi.org/doi:10.1002/hbm.22585

Rance, M., Zhao, Z., Zaboski, B., Kichuk, S. A., Romaker, E., Koller, W. N., Walsh, C., Harris-Starling, C., Wasylink, S., Adams, T., Gruner, P., Pittenger, C., & Hampson, M. (2023). Neurofeedback for obsessive compulsive disorder: A randomized, double-blind trial. *Psychiatry Research*, *328*. https://doi.org/10.1016/J.PSYCHRES.2023.115458

Robineau, F., Meskaldji, D. E., Koush, Y., Rieger, S. W., Mermoud, C., Morgenthaler, S., Van De Ville, D., Vuilleumier, P., & Scharnowski, F. (2017). Maintenance of Voluntary Self-regulation Learned through Real-Time fMRI Neurofeedback. *FRONTIERS IN HUMAN NEUROSCIENCE*, *11*. https://doi.org/doi:10.3389/fnhum.2017.00131

Robineau, F., Rieger, S. W., Mermoud, C., Pichon, S., Koush, Y., Van De Ville, D., Vuilleumier, P., & Scharnowski, F. (2014). Self-regulation of inter-hemispheric visual cortex balance through real-time fMRI neurofeedback training. *Neuroimage*, *100*, 1–14. https://doi.org/doi:10.1016/j.neuroimage.2014.05.072

Robineau, F., Saj, A., Neveu, R., Van De Ville, D., Scharnowski, F., & Vuilleumier, P. (2019). Using real-time fMRI neurofeedback to restore right occipital cortex activity in patients with left visuo-spatial neglect: proof-of-principle and preliminary results. *NEUROPSYCHOLOGICAL REHABILITATION*, *29*(3), 339–360. https://doi.org/doi:10.1080/09602011.2017.1301262

Rota, G., Sitaram, R., Veit, R., Erb, M., Weiskopf, N., Dogil, G., & Birbaumer, N. (2009). Self-Regulation of Regional Cortical Activity Using Real-Time fMRI: The Right Inferior Frontal Gyrus and Linguistic Processing. *HUMAN BRAIN MAPPING*, *30*(5), 1605–1614. https://doi.org/doi:10.1002/hbm.20621

Ruiz, S., Lee, S., Soekadar, S. R., Caria, A., Veit, R., Kircher, T., Birbaumer, N., & Sitaram, R. (2013). Acquired self-control of insula cortex modulates emotion recognition and brain network connectivity in schizophrenia. *HUMAN BRAIN MAPPING*, *34*(1), 200–212. https://doi.org/doi:10.1002/hbm.21427

Russo, A. G., Luhrs, M., Di Salle, F., Esposito, F., & Goebel, R. (2021). Towards semantic fMRI neurofeedback: navigating among mental states using real-time representational similarity analysis. *JOURNAL OF NEURAL ENGINEERING*, *18*(4). https://doi.org/doi:10.1088/1741-2552/abecc3

Sampaio-Baptista, C., Neyedli, H. F., Sanders, Z.-B., Diosi, K., Havard, D., Huang, Y., Andersson, J. L. R., Luhr, M., Goebel, R., & Johansen-Berg, H. (2021). fMRI neurofeedback in the motor system elicits bidirectional changes in activity and in white matter structure in the adult human brain. *CELL REPORTS*, *37*(4). https://doi.org/doi:10.1016/j.celrep.2021.109890

Sanders, Z. B., Fleming, M. K., Smejka, T., Marzolla, M. C., Zich, C., Rieger, S. W., Luhrs, M., Goebel, R., Sampaio-Baptista, C., & Johansen-Berg, H. (2022). Self-modulation of motor cortex activity after stroke: a randomized controlled trial. *BRAIN*, *145*(10), 3391–3404. https://doi.org/doi:10.1093/brain/awac239

Sarkheil, P., Zilverstand, A., Kilian-Hutten, N., Schneider, F., Goebel, R., & Mathiak, K. (2015). fMRI feedback enhances emotion regulation as evidenced by a reduced amygdala response. *BEHAVIOURAL BRAIN RESEARCH*, *281*, 326–332. https://doi.org/doi:10.1016/j.bbr.2014.11.027

Saxena, A., Shovestul, B. J., Dudek, E. M., Reda, S., Venkataraman, A., Lamberti, J. S., & Dodell-Feder, D. (2023). Training volitional control of the theory of mind network with real-time fMRI neurofeedback. *NeuroImage*, *279*(March), 120334. https://doi.org/10.1016/j.neuroimage.2023.120334

Scharnowski, F., Hutton, C., Josephs, O., Weiskopf, N., & Rees, G. (2012). Improving Visual Perception through Neurofeedback. *JOURNAL OF NEUROSCIENCE*, *32*(49), 17830–17841. https://doi.org/doi:10.1523/JNEUROSCI.6334-11.2012

Scharnowski, F., Veite, R., Zopf, R., Studer, P., Bock, S., Diedrichsen, J., Goebel, R., Mathiak, K., Birbaumer, N., & Weiskopf, N. (2015). Manipulating motor performance and memory through real-time fMRI neurofeedback. *BIOLOGICAL PSYCHOLOGY*, *108*, 85–97. https://doi.org/doi:10.1016/j.biopsycho.2015.03.009

Scheinost, D., Hsu, T. W., Avery, E. W., Hampson, M., Constable, R. T., Chun, M. M., & Rosenberg, M. D. (2020). Connectome-based neurofeedback: A pilot study to improve sustained attention. *Neuroimage*, *212*. https://doi.org/doi:10.1016/j.neuroimage.2020.116684

Scheinost, D., Stoica, T., Saksa, J., Papademetris, X., Constable, R. T., Pittenger, C., & Hampson, M. (2013). Orbitofrontal cortex neurofeedback produces lasting changes in contamination anxiety and resting-state connectivity. *TRANSLATIONAL PSYCHIATRY*, *3*. https://doi.org/doi:10.1038/tp.2013.24

Sepulveda, P., Sitaram, R., Rana, M., Montalba, C., Tejos, C., & Ruiz, S. (2016). How Feedback, Motor Imagery, and Reward Influence Brain Self-Regulation Using Real-Time fMRI. *HUMAN BRAIN MAPPING*, *37*(9), 3153–3171. https://doi.org/doi:10.1002/hbm.23228

Sherwood, M. S., Kane, J. H., Weisend, M. P., & Parker, J. G. (2016). Enhanced control of dorsolateral prefrontal cortex neurophysiology with real-time functional magnetic resonance imaging (rt-fMRI) neurofeedback training and working memory practice. *Neuroimage*, *124*, 214–223. https://doi.org/doi:10.1016/j.neuroimage.2015.08.074

Sherwood, M. S., Parker, J. G., Diller, E. E., Ganapathy, S., Bennett, K. B., Esquivel, C. R., & Nelson, J. T. (2019). Self-directed down-regulation of auditory cortex activity mediated by real-time fMRI neurofeedback augments attentional processes, resting cerebral perfusion, and auditory activation. *Neuroimage*, *195*, 475–489. https://doi.org/doi:10.1016/j.neuroimage.2019.03.078

Sherwood, M. S., Parker, J. G., Diller, E. E., Ganapathy, S., Bennett, K., & Nelson, J. T. (2018). Volitional down-regulation of the primary auditory cortex via directed attention mediated by real-time fMRI neurofeedback. *AIMS Neuroscience*, *5*(3), 179–199. https://doi.org/doi:10.3934/Neuroscience.2018.3.179

Shibata, K., Watanabe, T., Kawato, M., & Sasaki, Y. (2016). Differential Activation Patterns in the Same Brain Region Led to Opposite Emotional States. *PLOS BIOLOGY*, *14*(9). https://doi.org/doi:10.1371/journal.pbio.1002546

Shibata, K., Watanabe, T., Sasaki, Y., & Kawato, M. (2011). Perceptual Learning Incepted by Decoded fMRI Neurofeedback Without Stimulus Presentation. *SCIENCE*, *334*(6061), 1413–1415. https://doi.org/doi:10.1126/science.1212003

Sitaram, R., Caria, A., Veit, R., Gaber, T., Ruiz, S., & Birbaumer, N. (2014). Volitional control of the anterior insula in criminal psychopaths using real-time fMRI neurofeedback: a pilot study. *FRONTIERS IN BEHAVIORAL NEUROSCIENCE*, *8*. https://doi.org/doi:10.3389/fnbeh.2014.00344

Sokunbi, M. O., Linden, D. E. J., Habes, I., Johnston, S., & Ihssen, N. (2014). Real-time fMRI brain-computer interface: development of a "motivational feedback" subsystem for the regulation of visual cue reactivity. *FRONTIERS IN BEHAVIORAL NEUROSCIENCE*, *8*. https://doi.org/doi:10.3389/fnbeh.2014.00392

Sorger, B., Kamp, T., Weiskopf, N., Peters, J. C., & Goebel, R. (2018). When the Brain Takes "BOLD" Steps: Real-Time fMRI Neurofeedback Can Further Enhance the Ability to Gradually Self-regulate Regional Brain Activation. *NEUROSCIENCE*, *378*, 71–88. https://doi.org/doi:10.1016/j.neuroscience.2016.09.026

Sousa, T., Direito, B., Lima, J., Ferreira, C., Nunes, U., & Castelo-Branco, M. (2016). Control of Brain Activity in hMT+/V5 at Three Response Levels Using fMRI-Based Neurofeedback/BCI. *PLoS One*, *11*(5). https://doi.org/doi:10.1371/journal.pone.0155961

Spetter, M. S., Malekshahi, R., Birbaumer, N., Luhrs, M., van der Veer, A. H., Scheffler, K., Spuckti, S., Preissl, H., Veit, R., & Hallschmid, M. (2017). Volitional regulation of brain responses to food stimuli in overweight and obese subjects: A real-time fMRI feedback study. *APPETITE*, *112*, 188–195. https://doi.org/doi:10.1016/j.appet.2017.01.032

Sreedharan, S., Chandran, A., Yanamala, V. R., Sylaja, P. N., Kesavadas, C., & Sitaram, R. (2020). Self-regulation of language areas using real-time functional MRI in stroke patients with expressive aphasia. *BRAIN IMAGING AND BEHAVIOR*, *14*(5), 1714–1730. https://doi.org/doi:10.1007/s11682-019-00106-7

Subramanian, L., Hindle, J. V, Johnston, S., Roberts, M. V, Husain, M., Goebel, R., & Linden, D. (2011). Real-Time Functional Magnetic Resonance Imaging Neurofeedback for Treatment of Parkinson's Disease. *JOURNAL OF NEUROSCIENCE*, *31*(45), 16309–16317. https://doi.org/doi:10.1523/JNEUROSCI.3498-11.2011

Subramanian, L., Morris, M. B., Brosnan, M., Turner, D. L., Morris, H. R., & Linden, D. E. J. (2016). Functional Magnetic Resonance Imaging Neurofeedback-guided Motor Imagery Training and Motor Training for Parkinson's Disease: Randomized Trial. *FRONTIERS IN BEHAVIORAL NEUROSCIENCE*, *10*. https://doi.org/doi:10.3389/fnbeh.2016.00111

Subramanian, L., Skottnik, L., Cox, W. M., Luhrs, M., McNamara, R., Hood, K., Watson, G., Whittaker, J. R., Williams, A. N., Sakhuja, R., Ihssen, N., Goebel, R., Playle, R., & Linden, D. E. J. (2021). Neurofeedback Training versus Treatment-as-Usual for Alcohol Dependence: Results of an Early-Phase Randomized Controlled Trial and Neuroimaging Correlates. *EUROPEAN ADDICTION RESEARCH*, *27*(5), 381–394. https://doi.org/doi:10.1159/000513448

Sukhodolsky, D. G., Walsh, C., Koller, W. N., Eilbott, J., Rance, M., Fulbright, R. K., Zhao, Z., Bloch, M. H., King, R., Leckman, J. F., Scheinost, D., Pittman, B., & Hampson, M. (2020). Randomized, Sham-Controlled Trial of Real-Time Functional Magnetic Resonance Imaging Neurofeedback for Tics in Adolescents With Tourette Syndrome. *BIOLOGICAL PSYCHIATRY*, *87*(12), 1063–1070. https://doi.org/doi:10.1016/j.biopsych.2019.07.035

Sulzer, J., Sitaram, R., Blefari, M. L., Kollias, S., Birbaumer, N., Stephan, K. E., Luft, A., & Gassert, R. (2013). Neurofeedback-mediated self-regulation of the dopaminergic midbrain. *Neuroimage*, *83*, 817–825. https://doi.org/doi:10.1016/j.neuroimage.2013.05.115

Takamura, M., Okamoto, Y., Shibasaki, C., Yoshino, A., Okada, G., Ichikawa, N., & Yamawaki, S. (2020). Antidepressive effect of left dorsolateral prefrontal cortex neurofeedback in patients with major depressive disorder: A preliminary report. *JOURNAL OF AFFECTIVE DISORDERS*, *271*, 224–227. https://doi.org/doi:10.1016/j.jad.2020.03.080

Taylor, J. E., Yamada, T., Kawashima, T., Kobayashi, Y., Yoshihara, Y., Miyata, J., Murai, T., Kawato, M., & Motegi, T. (2022). Depressive symptoms reduce when dorsolateral prefrontal cortex-precuneus connectivity normalizes

after functional connectivity neurofeedback. *SCIENTIFIC REPORTS*, *12*(1). https://doi.org/doi:10.1038/s41598-022-05860-1

Tinaz, S., Kamel, S., Aravala, S. S., Elfil, M., Bayoumi, A., Patel, A., Scheinost, D., Sinha, R., & Hampson, M. (2022). Neurofeedback-guided kinesthetic motor imagery training in Parkinson's disease: Randomized trial. *NEUROIMAGE-CLINICAL*, *34*. https://doi.org/doi:10.1016/j.nicl.2022.102980

Tinaz, S., Para, K., Vives-Rodriguez, A., Martinez-Kaigi, V., Nalamada, K., Sezgin, M., Scheinost, D., Hampson, M., Louis, E. D., & Constable, R. T. (2018). Insula as the Interface Between Body Awareness and Movement: A Neurofeedback-Guided Kinesthetic Motor Imagery Study in Parkinson's Disease. *FRONTIERS IN HUMAN NEUROSCIENCE*, *12*. https://doi.org/doi:10.3389/fnhum.2018.00496

Travassos, C., Sayal, A., Direito, B., Castelhano, J., & Castelo-Branco, M. (2020). Volitional Modulation of the Left DLPFC Neural Activity Based on a Pain Empathy Paradigm-A Potential Novel Therapeutic Target for Pain. *FRONTIERS IN NEUROLOGY*, *11*. https://doi.org/doi:10.3389/fneur.2020.00714

Tsuchiyagaito, A., Misaki, M., Kirlic, N., Yu, X., Sanchez, S. M., Cochran, G., Stewart, J. L., Smith, R., Fitzgerald, K. D., Rohan, M. L., Paulus, M. P., & Guinjoan, S. M. (2023). Real-Time fMRI Functional Connectivity Neurofeedback Reducing Repetitive Negative Thinking in Depression: A Double-Blind, Randomized, Sham-Controlled Proof-of-Concept Trial. *PSYCHOTHERAPY AND PSYCHOSOMATICS*, *92*(2), 87–100. https://doi.org/doi:10.1159/000528377

Tsuchiyagaito, A., Misaki, M., Zoubi, O. Al, Paulus, M., Bodurka, J., & Tulsa, I. (2021). Prevent breaking bad: A proof of concept study of rebalancing the brain's rumination circuit with real-time fMRI functional connectivity neurofeedback. *HUMAN BRAIN MAPPING*, *42*(4), 922–940. https://doi.org/doi:10.1002/hbm.25268

Van De Ville, D., Jhooti, P., Haas, T., Kopel, R., Lovblad, K.-O., Scheffler, K., & Haller, S. (2012). Recovery of the default mode network after demanding neurofeedback training occurs in spatio-temporally segregated subnetworks. *Neuroimage*, *63*(4), 1775–1781. https://doi.org/doi:10.1016/j.neuroimage.2012.08.061

Van den Boom, M. A., Jansma, J. M., & Ramsey, N. F. (2018). Rapid acquisition of dynamic control over DLPFC using real-time fMRI feedback. *EUROPEAN NEUROPSYCHOPHARMACOLOGY*, *28*(11), 1194–1205. https://doi.org/doi:10.1016/j.euroneuro.2018.08.508

Vargas, P., Sitaram, R., Sepulveda, P., Montalba, C., Rana, M., Torres, R., Tejos, C., & Ruiz, S. (2021). Weighted neurofeedback facilitates greater self-regulation of functional connectivity between the primary motor area and cerebellum. *JOURNAL OF NEURAL ENGINEERING*, *18*(5). https://doi.org/doi:10.1088/1741-2552/ac2b7e

Veit, R., Singh, V., Sitaram, R., Caria, A., Rauss, K., & Birbaumer, N. (2012). Using real-time fMRI to learn voluntary regulation of the anterior insula in the presence of threat-related stimuli. *Soc Cogn Affect Neurosci*, *7*(6), 623–634. https://doi.org/10.1093/scan/nsr061

Wang, T., Peeters, R., Mantini, D., & Gillebert, C. R. (2020). Modulating the interhemispheric activity balance in the intraparietal sulcus using real-time fMRI neurofeedback: Development and proof-of-concept. *NEUROIMAGE-CLINICAL*, *28*. https://doi.org/doi:10.1016/j.nicl.2020.102513

Wang, Y., Yao, L., & Zhao, X. (2020). Amygdala network in response to facial expression following neurofeedback training of emotion. *BRAIN IMAGING AND BEHAVIOR*, *14*(3), 897–906. https://doi.org/doi:10.1007/s11682-019-00052-4

Wang, Z., Tamaki, M., Frank, S. M., Shibata, K., Worden, M. S., Yamada, T., Kawato, M., Sasaki, Y., & Watanabe, T. (2021). Visual perceptual learning of a primitive feature in human V1/V2 as a result of unconscious processing, revealed by decoded functional MRI neurofeedback (DecNef). *JOURNAL OF VISION*, *21*(8). https://doi.org/doi:10.1167/jov.21.8.24

Weaver, S. S., Birn, R. M., & Cisler, J. M. (2020). A Pilot Adaptive Neurofeedback Investigation of the Neural Mechanisms of Implicit Emotion Regulation Among Women With PTSD. *FRONTIERS IN SYSTEMS NEUROSCIENCE*, *14*. https://doi.org/doi:10.3389/fnsys.2020.00040

Weiss, F., Zhang, J., Aslan, A., Kirsch, P., & Gerchen, M. F. (2022). Feasibility of training the dorsolateral prefrontal-striatal network by real-time fMRI neurofeedback. In *Scientific Reports* (Vol. 12, Issue 1).

https://doi.org/10.1038/s41598-022-05675-0

Xie, F., Xu, L., Long, Z., Yao, L., & Wu, X. (2015). Functional connectivity alteration after real-time fMRI motor imagery training through self-regulation of activities of the right premotor cortex. *BMC NEUROSCIENCE*, *16*. https://doi.org/doi:10.1186/s12868-015-0167-1

Yamashita, A., Hayasaka, S., Kawato, M., & Imamizu, H. (2017). Connectivity Neurofeedback Training Can Differentially Change Functional Connectivity and Cognitive Performance. *CEREBRAL CORTEX*, *27*(10), 4960–4970. https://doi.org/doi:10.1093/cercor/bhx177

Yang, H., Hu, Z., Imai, F., Yang, Y., & Ogawa, K. (2021). Effects of neurofeedback on the activities of motor-related areas by using motor execution and imagery. *NEUROSCIENCE LETTERS*, *746*. https://doi.org/doi:10.1016/j.neulet.2021.135653

Yao, S., Becker, B., Geng, Y., Zhao, Z., Xu, X., Zhao, W., Ren, P., & Kendrick, K. M. (2016). Voluntary control of anterior insula and its functional connections is feedback-independent and increases pain empathy. *Neuroimage*, *130*, 230–240. https://doi.org/doi:10.1016/j.neuroimage.2016.02.035

Yoo, S.-S., Lee, J.-H., O'Leary, H., Lee, V., Choo, S.-E., & Jolesz, F. A. (2007). Functional magnetic resonance imaging-mediated learning of increased activity in auditory areas. *NEUROREPORT*, *18*(18), 1915–1920. https://doi.org/doi:10.1097/WNR.0b013e3282f202ac

Yoo, S.-S., Lee, J.-H., O'Leary, H., Panych, L. P., & Jolesz, F. A. (2008). Neurofeedback fMRI-mediated learning and consolidation of regional brain activation during motor imagery. *INTERNATIONAL JOURNAL OF IMAGING SYSTEMS AND TECHNOLOGY*, *18*(1), 69–78. https://doi.org/doi:10.1002/ima.20139

Yoo, S.-S., O'Leary, H. M., Fairneny, T., Chen, N.-K., Panych, L. P., Park, H., & Jolesz, F. A. (2006). Increasing cortical activity in auditory areas through neurofeedback functional magnetic resonance imaging. *NEUROREPORT*, *17*(12), 1273–1278. https://doi.org/doi:10.1097/01.wnr.0000227996.53540.22

Yoo, S. S., & Jolesz, F. A. (2002). Functional MRI for neurofeedback: feasibility study on a hand motor task. *NEUROREPORT*, *13*(11), 1377–1381. https://doi.org/doi:10.1097/00001756-200208070-00005

Young, K. D., Prause, N., Lazzaro, S., & Siegle, G. J. (2020). Low Cost MR Compatible Haptic Stimulation with Application to fMRI Neurofeedback. *BRAIN SCIENCES*, *10*(11). https://doi.org/doi:10.3390/brainsci10110790

Young, K. D., Siegle, G. J., Zotev, V., Phillips, R., Misaki, M., Yuan, H., Drevets, W. C., & Bodurka, J. (2017). Randomized Clinical Trial of Real-Time fMRI Amygdala Neurofeedback for Major Depressive Disorder: Effectson Symptoms and Autobiographical Memory Recall. *AMERICAN JOURNAL OF PSYCHIATRY*, *174*(8), 748–755. https://doi.org/doi:10.1176/appi.ajp.2017.16060637

Young, K. D., Zotev, V., Phillips, R., Misaki, M., Yuan, H., Drevets, W. C., & Bodurka, J. (2014). Real-Time fMRI Neurofeedback Training of Amygdala Activity in Patients with Major Depressive Disorder. *PLoS One*, *9*(2). https://doi.org/doi:10.1371/journal.pone.0088785

Yu, X., Cohen, Z., Tsuchiyagaito, A., Cochran, G., Aupperle, R., Stewart, J., Singh, M., Misaki, M., Bodurka, J., Paulus, M., & Kirlic, N. (2022). Neurofeedback-Augmented Mindfulness Training Elicits Distinct Responses in the Subregions of the Insular Cortex in Healthy Adolescents. *BRAIN SCIENCES*, *12*(3). https://doi.org/doi:10.3390/brainsci12030363

Yuan, H., Young, K. D., Phillips, R., Zotev, V., Misaki, M., & Bodurka, J. (2014). Resting-state functional connectivity modulation and sustained changes after real-time functional magnetic resonance imaging neurofeedback training in depression. *Brain Connectivity*, *4*(9), 690–701. https://doi.org/doi:10.1089/brain.2014.0262

Zaehringer, J., Ende, G., Santangelo, P., Kleindienst, N., Ruf, M., Bertsch, K., Bohus, M., Schmahl, C., & Paret, C. (2019). Improved emotion regulation after neurofeedback: A single-arm trial in patients with borderline personality disorder. *NEUROIMAGE-CLINICAL*, *24*. https://doi.org/doi:10.1016/j.nicl.2019.102032

Zahn, R., Weingartner, J. H., Basilio, R., Bado, P., Mattos, P., Sato, J. R., de Oliveira-Souza, R., Fontenelle, L. F., Young, A. H., & Moll, J. (2019). Blame-rebalance fMRI neurofeedback in major depressive disorder: A randomised proof-of-concept trial. *NEUROIMAGE-CLINICAL*, *24*. https://doi.org/doi:10.1016/j.nicl.2019.101992

Zhang, G., Yao, L., Zhang, H., Long, Z., & Zhao, X. (2013). Improved Working Memory Performance through Self-Regulation of Dorsal Lateral Prefrontal Cortex Activation Using Real-Time fMRI. *PLoS ONE*, *8*(8), 1–9. https://doi.org/10.1371/journal.pone.0073735

Zhang, G., Zhang, H., Li, X., Zhao, X., Yao, L., & Long, Z. (2013). Functional Alteration of the DMN by Learned Regulation of the PCC Using Real-Time fMRI. *IEEE TRANSACTIONS ON NEURAL SYSTEMS AND REHABILITATION ENGINEERING*, *21*(4), 595–606. https://doi.org/doi:10.1109/TNSRE.2012.2221480

Zhang, J., Raya, J., Morfini, F., Urban, Z., Pagliaccio, D., Yendiki, A., Auerbach, R. P., Bauer, C. C. C., & Whitfield-Gabrieli, S. (2023). Reducing default mode network connectivity with mindfulness-based fMRI neurofeedback: a pilot study among adolescents with affective disorder history. *MOLECULAR PSYCHIATRY*, *28*(6), 2540–2548. https://doi.org/doi:10.1038/s41380-023-02032-z

Zhang, S., Yoshida, W., Mano, H., Yanagisawa, T., Mancini, F., Shibata, K., Kawato, M., & Seymour, B. (2020). Pain Control by Co-adaptive Learning in a Brain-Machine Interface. *CURRENT BIOLOGY*, *30*(20), 3935-+. https://doi.org/doi:10.1016/j.cub.2020.07.066

Zhang, Y., Zhang, Q., Wang, J., Zhou, M., Qing, Y., Zou, H., Li, J., Yang, C., Becker, B., Kendrick, K. M., & Yao, S. (2023). "Listen to your heart": A novel interoceptive strategy for real-time fMRI neurofeedback training of anterior insula activity. *Neuroimage*, *284*, 120455. https://doi.org/doi:10.1016/j.neuroimage.2023.120455

Zhao, X., Zhang, H., Song, S., Ye, Q., Guo, J., & Yao, L. (2013). Causal interaction following the alteration of target region activation during motor imagery training using real-time fMRI. *Front Hum Neurosci*, *7*, 866. https://doi.org/10.3389/fnhum.2013.00866

Zhao, Z., Duek, O., Seidemann, R., Gordon, C., Walsh, C., Romaker, E., Koller, W. N., Horvath, M., Awasthi, J., Wang, Y., O'Brien, E., Fichtenholtz, H., Hampson, M., & Harpaz-Rotem, I. (2023). Amygdala downregulation training using fMRI neurofeedback in post-traumatic stress disorder: a randomized, double-blind trial. *TRANSLATIONAL PSYCHIATRY*, *13*(1). https://doi.org/doi:10.1038/s41398-023-02467-6

Zhao, Z., Yao, S., Li, K., Sindermann, C., Zhou, F., Zhao, W., Li, J., Luhrs, M., Goebel, R., Kendrick, K. M., & Becker, B. (2019). Real-Time Functional Connectivity-Informed Neurofeedback of Amygdala-Frontal Pathways Reduces Anxiety. *PSYCHOTHERAPY AND PSYCHOSOMATICS*, *88*(1), 5–15. https://doi.org/doi:10.1159/000496057

Zhu, Y., Gao, H., Tong, L., Li, Z., Wang, L., Zhang, C., Yang, Q., & Yan, B. (2019). Emotion Regulation of Hippocampus Using Real-Time fMRI Neurofeedback in Healthy Human. *FRONTIERS IN HUMAN NEUROSCIENCE*, *13*. https://doi.org/doi:10.3389/fnhum.2019.00242

Zich, C., Johnstone, N., Luhrs, M. D., Lisk, S. T., Haller, S. P. W., Lipp, A., Lau, J. Y. F., & Kadosh, K. C. (2020). Modulatory effects of dynamic fMRI-based neurofeedback on emotion regulation networks in adolescent females. *Neuroimage*, *220*. https://doi.org/doi:10.1016/j.neuroimage.2020.117053

Zilverstand, A., Sorger, B., Sarkheil, P., & Goebel, R. (2015). fMRI neurofeedback facilitates anxiety regulation in females with spider phobia. *FRONTIERS IN BEHAVIORAL NEUROSCIENCE*, *9*. https://doi.org/doi:10.3389/fnbeh.2015.00148

Zilverstand, A., Sorger, B., Slaats-Willemse, D., Kan, C. C., Goebel, R., & Buitelaar, J. K. (2017). fMRI neurofeedback training for increasing anterior cingulate cortex activation in adult attention deficit hyperactivity disorder. An exploratory randomized, single-blinded study. *PLoS ONE*, *12*(1), 1–23. https://doi.org/10.1371/journal.pone.0170795

Zotev, V., Krueger, F., Phillips, R., Alvarez, R. P., Simmons, W. K., Bellgowan, P., Drevets, W. C., & Bodurka, J. (2011). Self-Regulation of Amygdala Activation Using Real-Time fMRI Neurofeedback. *PLoS One*, *6*(9). https://doi.org/doi:10.1371/journal.pone.0024522

Zweerings, J., Hummel, B., Keller, M., Zvyagintsev, M., Schneider, F., Klasen, M., & Mathiak, K. (2019). Neurofeedback of core language network nodes modulates connectivity with the default-mode network: A double-blind fMRI neurofeedback study on auditory verbal hallucinations. *Neuroimage*, *189*, 533–542. https://doi.org/doi:10.1016/j.neuroimage.2019.01.058

Zweerings, J., Pflieger, E. M., Mathiak, K. A., Zvyagintsev, M., Kacela, A., Flatten, G., & Mathiak, K. (2018). Impaired

Voluntary Control in PTSD: Probing Self-Regulation of the ACC With Real-Time fMRI. *FRONTIERS IN PSYCHIATRY*, *9*. https://doi.org/doi:10.3389/fpsyt.2018.00219

Zweerings, J., Sarkheil, P., Keller, M., Dyck, M., Klasen, M., Becker, B., Gaebler, A. J., Ibrahim, C. N., Turetsky, B. I., Zvyagintsev, M., Flatten, G., & Mathiak, K. (2020). Rt-fMRI neurofeedback-guided cognitive reappraisal training modulates amygdala responsivity in posttraumatic stress disorder. *NEUROIMAGE-CLINICAL*, *28*. https://doi.org/doi:10.1016/j.nicl.2020.102483
